# Traversing the solution space of neural networks with Hessian Null Space Continuation

**Published:** 2026-09-29

**Authors:** Ann Huang, Mitchell Ostrow, Zhouyang Lu, William T. Redman, Leo Kozachkov, Kanaka Rajan

**Affiliations:** 1 Harvard University; 2 Harvard Medical School; 3 Kempner Institute; 4 Massachusetts Institute of Technology; 5 Brown University; 6 Johns Hopkins University

## Abstract

On a single task, deep neural networks can learn a wide range of solutions, depending on their optimizer, training data, architecture, and hyperparameters. Many of these solutions are surprisingly *mode-connected*: rather than isolated points in the weight space, they are connected by low loss regions. Despite this observation, the diversity of solutions in terms of their internal computation in these regions has not been characterized. A parallel line of work has identified the *degeneracy of neural representations*: many neural network solutions exist with similar training loss yet distinct internal structures. However, it is unclear how these diverse solutions are related in weight space. Here, we unify these sub-fields and demonstrate for the first time that there exist many different internal mechanisms within a local mode connected region in weight space. To do so, we introduce Hessian Null Space Continuation (HNC), a scalable method that uses local curvature information to traverse regions of weight space that preserve network function. HNC can additionally be steered toward solutions with specified properties. In RNNs trained on a memory task, HNC drives the networks to learn drastically different representations and dynamics, even with maintained behavior. In ImageNet-trained Vision Transformers, HNC finds alternative representations which differ more from the original network than any independently trained models of different architectures and training objectives. In reinforcement-learning agents, HNC uncovers a distinct behavioral strategy at comparable return in a navigation task, and exposes a reward-hacking strategy in an AI Safety Gridworld environment. Finally, HNC provides local geometric information about the solution distribution, showing how model size and task complexity shape its dimension and functional sensitivity. Together, our results show that a surprisingly large amount of representational diversity exists near a single trained solution, which is unseen by standard gradient-based optimization techniques. Our domain-agnostic method, HNC, can identify and quantify this diversity, opening new possibilities for mechanistic understanding of solution spaces and providing a principled basis for model merging, editing, and fine-tuning. Project page and code available at ann-huang-0.github.io/Hessian-null-space-continuation.

## 1 Introduction

Overparameterized neural networks can admit many local minima with similarly low loss. A major line of work in machine learning therefore asks how the optimizer, training data, architecture, and task shape which local minimum is reached by training. Investigation into this has shed fundamental light on the implicit bias of stochastic gradient descent towards flatter minima (Kleinberg et al., 2018; Feng & Tu, 2021) and the converging representations between networks as models get larger and tasks get more complex (Huh et al., 2024).

A classic picture of the loss landscape depicts local minima as isolated basins that are well separated in parameter space (Li et al., 2018). This intuition, however, is grounded in low dimensions and convex optimization, and becomes unreliable in the extremely high-dimensional, nonconvex landscapes of modern over-parameterized networks (Sagun et al., 2016; 2017; Gur-Ari et al., 2018). Studies of local loss geometry have revealed many nearly flat directions around trained, low-loss solutions (Sagun et al., 2016; 2017; Chaudhari et al., 2017; Ghorbani et al., 2019). The mode connectivity literature has also shown that independently trained networks are in fact often connected in weight space: nonlinear paths of non-increasing loss provably exist between minima of fully connected neural networks (Freeman & Bruna, 2017; Draxler et al., 2018; Garipov et al., 2018), and some minima are even connected by linear paths with negligible loss barriers (linear mode connectivity, Frankle et al. (2020)). These results suggest that apparently distinct minima may lie within a common connected region of low loss in weight space. However, this literature has largely characterized that region through connectivity and loss alone, rather than the internal computations that networks within it implement. Lubana et al. (2023) took an important step in this direction, showing that linear mode connectivity between two networks can designate whether they share similar input attribution functions, and that nonlinear low-loss paths can connect even dissimilar minima. Yet these analyses studied pairs of networks identified via training, rather than systematically examining the low-loss region surrounding a solution. It remains unknown whether the connected low-loss region around a single trained solution contains genuinely distinct representations and mechanisms, or only minor modifications of the same representation and mechanism.

Meanwhile, an emerging line of work suggests that a single task can admit a family of different solutions (Goldman, 2009; Huang et al., 2025; D’Amour et al., 2022; Turner et al., 2021; Lappalainen et al., 2024; Kurtkaya et al., 2025; Murray, 2025; Clark et al., 2026; Zhong et al., 2023; Saxe et al., 2022; Ostrow et al., 2026), characterized by distinct out-of-distribution (OOD) generalization behavior (Huang et al., 2025; D’Amour et al., 2022; Ostrow et al., 2026) and distinct geometric and dynamical properties of the internal representations (Huang et al., 2025; Turner et al., 2021; Kurtkaya et al., 2025; Murray, 2025; Clark et al., 2026; Ostrow et al., 2026). Such solution degeneracy is further supported by work showing that function and representations can doubly dissociate (Braun et al., 2025), even in nonlinear neural networks (Theiss et al., 2026a;b). Beyond standard solutions reached by gradient descent during training, recent work has deliberately sought to find alternative solutions to the same task by training with regularization that either penalizes similarity to a standard solution (Qian & Pehlevan, 2026; Ostrow et al., 2026) or makes the desired representational similarity score a hard constraint (Braun et al., 2025; Theiss et al., 2026b), or by following eigenvectors of the Hessian from a saddle point toward distinct minima (Parker-Holder et al., 2020). Relatedly, Gan & Isola (2026) showed that the local neighborhood of a single pretrained model can contain diverse experts with improved performance on specific tasks, suggesting that meaningfully different solutions may be accessible nearby in weight space. However, the relationship among these alternative solutions in parameter space remains poorly understood. In particular, **how much representational diversity exists within the connected, function-preserving region surrounding a single trained solution, and how can that region be traversed constructively?**

The set of solutions a given neural network model learns is constrained not only by the task constraints (Cao & Yamins, 2024; Yamins & Nayebi, 2026), but also the biases induced by optimizers. For example, stochastic gradient descent is known to induce low-rank, low-norm solutions (Soudry et al., 2018; Gunasekar et al., 2017; Arora et al., 2019; Woodworth et al., 2020; Huh et al., 2023; Vardi, 2023). Characterizing the broader set of solutions that a model *admits* enables dissociation from optimizer constraints and therefore unbiased analysis of shared structure across all solutions via task demands. On the practical side, if representationally distinct solutions are reachable from a learned solution through local, function-preserving weight updates, this exposes degrees of freedom that could be exploited for fine-tuning (Aghajanyan et al., 2021; Hu et al., 2022; Gan & Isola, 2026; Qiu et al., 2026; Liang et al., 2026), model merging (Wortsman et al., 2022; Ilharco et al., 2023), and post-hoc model editing (Meng et al., 2022; Mitchell et al., 2022).

To address these questions, we developed Hessian Null Space Continuation (HNC), a generic, model-agnostic method for identifying diverse solutions in neural networks. Starting from one base trained network (which we call the anchor), HNC explores a connected set of function-preserving alternatives using local curvature information. It can additionally be steered by the gradient of any differentiable function to find solutions with specific properties. In RNNs trained on a memory task, HNC transforms the canonical fixed-point solution of Sussillo & Barak (2013) into alternatives with qualitatively different geometry and dynamics. In ImageNet-trained Vision Transformers (ViTs), HNC finds nearby representations which differ more from the original network than any independently trained models of different architectures and training objectives, and even untrained models. This reveals substantial representational freedom inside neural networks underexplored by gradient training. We further extend HNC to reinforcement learning (RL), where it identifies policies that achieve comparable return through distinct behavioral strategies, including reward-hacking solutions. Together, our results show that an unexpectedly large amount of representational diversity (both in quantity and quality) exists near a single trained solution, which is unseen by standard optimization techniques. Our domain general method, HNC, can identify and quantify this diversity.

## 2 Method

### 2.1 Loss landscape curvature reveals function-preserving directions

Starting from a trained network $\theta_{0}$, the anchor, we seek networks with different internal representations but matched behavior. We operationalize this through the function-matching loss

(1) $$L(\theta )=\frac{1}{2}E_{x∼D}\left[{\left\|f_{\theta }(x)-f_{\theta_{0}}(x)\right\|}^{2}\right],$$

which measures output drift from the anchor over an input distribution D. In practice, we estimate this expectation using a fixed set X sampled from D, which we call the probe set. L is nonnegative and $L\left(\theta_{0}\right)=0$, so the anchor is a global minimizer and $\nabla L\left(\theta_{0}\right)=0$. A small weight perturbation δ thus changes the loss only at second order:

(2) $$L\left(\theta_{0}+\delta \right)=\frac{1}{2}\delta^{⊤}H\delta +O\left(\|\delta {\|}^{3}\right),\;H=\nabla_{\theta }^{2}L\left(\theta_{0}\right).$$

The eigenvalues of H therefore measure the network’s local sensitivity to parameter change along different parameter directions. Large eigenvalues identify sharp directions along which the network outputs change rapidly, whereas near-zero eigenvalues identify flat directions with little output drift. We define their span, $V_{0}=span\left\{v_{i}:\lambda_{i}\leq \epsilon \right\}$, as the approximate null space. In trained networks, the Hessian typically has a few large eigenvalues and many near-zero eigenvalues, so this approximate null space often spans a substantial fraction of the parameter dimensions (Sagun et al., 2017; Gur-Ari et al., 2018; Liang et al., 2026). Locally, $V_{0}$ consists of directions along which HNC can move the weights while approximately preserving the network function, allowing it to search for solutions with different internal representations. We discuss our choice of ϵ later in Section 2.2.1.

### 2.2 Hessian null space continuation

A single step within $V_{0}$ explores only the immediate neighborhood of the anchor. Exploring the function-preserving parameter set more extensively therefore requires a multi-step procedure. However, because the Hessian provides only a second-order approximation to the function-matching loss in Eq. 1, even a step along an eigenvector with zero eigenvalue can change the loss through higher-order terms neglected by Eq. 2. Moreover, the Hessian is a local approximation and its null space may cease to align with the flat directions away from $\theta_{0}$.

HNC addresses both points by alternating between a null space step and a function restoration step (Fig. 1). At each iteration t, we (1) take a small step within the current null space $V_{0}$, and then (2) restore function matching by taking a few gradient-descent steps on the loss in Eq. 1:

(3) $${\tilde{\theta }}_{t}=\theta_{t}+\eta d_{t},\;\theta_{t+1}={\tilde{\theta }}_{t}-\alpha \nabla L\left({\tilde{\theta }}_{t}\right),$$

where $d_{t}\in V_{0}$ is the step direction sampled uniformly from $V_{0}$, and η,α are the step sizes. For clarity, Eq. 3 shows a single restoration gradient step, and in practice we apply m such steps. The restoration step corrects the higher-order drift introduced by the preceding null space step. When the loss after a function restoration step exceeds a threshold, $L\left(\theta_{t+1}\right)>\tau$, this indicates that our linear approximation by the Hessian has become a poor fit of the local loss landscape. In these instances, we (3) relinearize by recomputing the Hessian null space at the current weights. We repeat this procedure until reaching either a prespecified number of steps or an alternative solution with the desired properties. In this way, HNC can move far from the anchor, while keeping L close to zero throughout. At a high level, HNC shares conceptual similarity with the classical predict-correct structure of the *numerical continuation* method, which traces a curve by taking a small step along its local tangent and then correcting back onto the curve (Keller, 1977; Allgower & Georg, 2003).

#### 2.2.1 Forming the null space

Because the function-matching loss has its exact minimum at $\theta_{0}$, its Hessian there is positive semidefinite, with all eigenvalues nonnegative (Appendix B). The null space $V_{0}$ is therefore the span of the eigenvectors with the numerically smallest eigenvalues. For a network with P parameters, H is a P×P matrix and is too large to store or eigendecompose directly. We compute the k smallest eigenpairs using the locally optimal block preconditioned conjugate gradient method (LOBPCG; Appendix A.2; Knyazev, 2001), which uses Hessian-vector products computed through automatic differentiation without explicitly forming the Hessian (Pearlmutter, 1994). Time and memory then scale as O(kP) rather than $O\left(P^{3}\right)$ and $O\left(P^{2}\right)$. A direction counts as flat when $\lambda_{i}\leq \mu_{\text{rel}}\lambda_{1}$, where $\lambda_{1}$ is the largest Hessian eigenvalue at the current linearization point and $\mu_{\text{rel}}\in \left[10^{-7},10^{-3}\right]$ is a relative threshold chosen per experiment. Because the threshold is relative, the null space is recomputed at every linearization. Sweeps over $\mu_{\text{rel}}$ are presented in Appendix F.2 and Appendix C.

#### 2.2.2 Steering

HNC can be steered to optimize a differentiable objective φ while preserving the network’s outputs, through projecting its gradient ∇φ onto the Hessian null space. To retain ∇φ along flat parameter directions and suppress those along sharp directions, we use a *soft projection*. Specifically, we rescale the gradient component along each Hessian eigenvector $v_{i}$ by $1/\left(1+\lambda_{i}/\mu \right)$, where $\lambda_{i}$ is its eigenvalue and μ>0 is a damping parameter: components along flat directions $\left(\lambda_{i}\ll \mu \right)$ pass through, while those along sharp directions $\left(\lambda_{i}\gg \mu \right)$ are suppressed. Since the gradient component along the eigenvector $v_{i}$ is $\left(v_{i}^{⊤}\nabla \varphi \right)v_{i}$, rescaling each component and summing over parameter directions therefore gives $d=\sum_{i}\;\frac{v_{i}^{⊤}\nabla \varphi }{1+\lambda_{i}/\mu }v_{i}=(I+H/\mu {)}^{-1}\nabla \varphi$. This matrix form allows us to bypass the need to explicitly construct the eigenbasis of the Hessian. We set the damping factor to the flatness threshold of Sec. 2.2.1, $\mu =\mu_{rel}\lambda_{1}$, so that a single hyperparameter $\mu_{rel}$ defines both which directions count as flat and which gradient directions are retained through soft projection.

## 3 HNC drives RNNS to learn drastically different dynamics in a memory task

We first applied HNC to small, interpretable RNNs trained on a memory task. RNN computations can be analyzed as a dynamical system: hidden-state trajectories and stable states reveal how networks store and update information (Sussillo & Barak, 2013; Mante et al., 2013). We use the 3-Bit Flip-Flop (3BFF) task from computational neuroscience (Sussillo & Barak, 2013), in which networks maintain three binary memories, each storing the last nonzero input on one input-output channel (Fig. 2A, left). We trained 64-unit *tanh* RNNs using backpropagation through time (BPTT) and the Adam optimizer to minimize the mean-squared error between target and network outputs. After training, the networks represented the 2^3^ = 8 memory states as eight stable fixed points, which are hidden-states that remain unchanged when the input is turned off. These fixed points formed the vertices of a three-dimensional cube in activation space (Fig. 2A, right). The Hessian spectrum falls from a few sharp directions into a broad near-zero bulk, with a participation ratio of about 23 across the five anchors (Appendix F.2, Fig. 15).

Next, we aim to characterize both alternative 3BFF solutions accessible through undirected null space exploration, and those that differ maximally from the anchor. For the latter, we steer toward maximizing one of two dissimilarity metrics: the CKA distance, defined as 1 − CKA, which measures how much the representational geometry (the kernel of the hidden activations) has changed (Kornblith et al., 2019), or the DSA distance, which measures how much the recurrent dynamics have changed up to invertible transformations (Ostrow et al., 2023; 2026). We applied HNC to five trained networks (the “anchors”) with adaptive step size and periodic relinearization (see Appendix F.3 for details). All HNC walks maintain task loss below 0.01, while steered walks steadily increase representational and dynamical distances from their respective anchors (Fig. 2B). While undirected walks reach representational and dynamical distances comparable to those between independently trained networks, steered walks consistently achieve greater divergence with less weight movement. During undirected exploration, the eight fixed points disappeared, replaced by a continuous manifold with eight distinct regions, one for each memory state (Fig. 2C). Meanwhile, the CKA- and DSA-steered HNC walks altered the number of fixed points within the network and produced visually distinct state space structure as shown in Fig. 2C.

To understand how the alternative networks maintain memory, we examined the network reached during the undirected exploration. During input-free memory periods, its hidden-states form eight compact points in the readout subspace but continue moving within eight distinct clusters in the readout-null subspace (Fig. 2D, left and middle). Further quantifying the speed of hidden-state movement, the alternative solution’s hidden-state continues to drift without input, mainly in the readout null space (Fig. 2D, right). Thus, this network maintains memory within distinct regions of state-space rather than at fixed points, preserving stable outputs despite ongoing internal dynamics. We further assessed the alternative solution’s memory retention and robustness to perturbations, showing that the CKA-steered endpoint is less robust to hidden-state perturbations, whereas the DSA-steered endpoint shows memory drift during prolonged memory periods (Fig. 2E). This highlights that even networks with matching in-distribution input-output mappings can behave differently under extended or perturbed conditions. Finally, to visualize the structure of the solution set reached by HNC, we computed multidimensional scaling (MDS) embeddings of 131 networks, based on pairwise representational distance (1 − CKA) or relative weight distance (Fig. 2F). These networks comprise the anchor, all checkpoints from three HNC walks of each type (undirected, CKA-steered, DSA-steered), and ten independently trained seeds. All networks solve the task with accuracy above 0.999. In representation space, the independently trained and undirected-walk networks sit close to the anchor, while the steered walks extend far beyond them into different parts of the solution space. In weight space, by contrast, the steered walks stay closer to the anchor than both the undirected walks and the spread among independent seeds, demonstrating a clear decoupling of representational from weight-space distance. The solutions do not collapse onto a single tight cluster in representation space, highlighting the diversity of solutions accessible by HNC within the connected low-loss region around one trained network.

## 4 HNC reveals underexplored degrees of freedom in VITS

The Platonic Representation Hypothesis (PRH) (Huh et al., 2024) argues that representations become increasingly similar across models as models scale and tasks get harder. Huh et al. (2024) attribute this to a convergence to the shared underlying “platonic” representation of reality that models approach. Here, we ask how much representational freedom remains within a trained model when its function is held approximately fixed. To test this, we take a ViT-S/16 (22M parameters, Dosovitskiy et al. (2021)) pretrained on ImageNet classification and search for maximally different representations at preserved input-output mapping. Concretely, we applied HNC to the pretrained ViT and steered to minimize the max-over-layers CKA similarity between the alternative representation and the anchor. In Appendix G.3, steering against the max-over-layers mutual k-nearest-neighbor (kNN) score, the metric used in Huh et al. (2024), gives the same qualitative results.

Starting from the pretrained ViT, we evaluated the anchor on 512 images from the Places-365 validation set (Zhou et al., 2018), the dataset on which the PRH was evaluated (Huh et al., 2024). The function-matching loss in Eq. 1 is evaluated over the anchor and alternative network’s logits on these same images. We record the CLS-token features after every transformer block together with the final pre-logits features, and steer the walk to reduce the highest CKA between any layer of the alternative network and any layer of the anchor. Because the hard maximum is not differentiable, we steer using a smooth softmax CKA surrogate while reporting the hard maximum CKA throughout (Appendix G.1). During HNC, the representational similarity between the anchor and the alternative network falls steadily, both on the images the walk was steered on (the probe set) and on images never seen during HNC (the held-out set) (Fig. 3A). The max-over-layers kNN score also falls to a comparable extent even though the walk never steers it directly (Fig. 3A), indicating that HNC reorganizes its image representations in general rather than exploiting a particular metric on a particular image set. Throughout the walk, the alternative networks keep the anchor’s top-1 predictions on the probe images. When evaluated on ImageNet, which was never used to constrain the HNC, the top-1 accuracy drops by less than 1% and top-5 accuracy remains nearly unchanged (Fig. 3B).

After applying HNC, the network’s weights have changed by only 1.3% in norm, yet its resulting representations have become less similar to the anchor’s than every trained vision model we compare against, spanning different sizes, architectures, training objectives, and training data (Fig. 3C). More remarkably, it is even less similar than comparing a randomly initialized ViT to the anchor (Fig. 3C). The pairwise cosine similarities between image representations on the probe set change drastically from the anchor to the HNC endpoint (Fig. 3D). A multidimensional scaling (MDS) embedding of 20 images from four classes further reveals that HNC reorganizes the neighborhood structure both within and across classes (Fig. 3E). To identify which aspects of visual similarity change, we select the five most similar image pairs in the anchor and HNC endpoint. We then score each pair’s similarity based on either their semantic content (DINOv2), color composition, or spatial layout (Appendix G.2). Like the anchor, the HNC endpoint’s most similar image pairs remain close in semantic content and color. However, they have lower spatial-layout similarity, approaching the random-pair baseline (Fig. 3F), indicating that the endpoint can represent images with different spatial layouts as similar. Overall, HNC reveals substantial representational freedom within a single model that is underexplored by standard gradient-based training, even in ViTs trained on challenging tasks. This suggests that rather than interpreting cross-model convergence as evidence for a unique underlying representation fully specified by the task, the observed similarity may arise due to an optimization bias that samples a narrow subset of solutions.

## 5 HNC finds behaviorally distinct, high-reward policies in reinforcement learning

Unlike supervised learning, where the target output for each data sample is precisely defined, in reinforcement learning (RL), the scalar reward rarely fully specifies the optimal sequences of actions over an episode. This underspecification can admit many behaviorally distinct policies with similar reward level, even unintended strategies that exploit the reward function rather than accomplish the intended task, a phenomenon known as reward hacking. We therefore apply HNC to search for behaviorally distinct solutions that achieve comparable return to a trained policy.

Let $\pi_{\theta_{0}}$ be the policy anchor. We seek weights θ whose actions differ from the anchor’s while the return is unchanged. The true reward function is usually unknown, but from an agent’s perspective, the return can be approximated by the off-policy importance sampling surrogate. Given a buffer of state-action pairs ${\left\{\left(s_{i},a_{i}\right)\right\}}_{i=1}^{N}$ collected from rollouts of the anchor policy, we aim to preserve the surrogate reward $\phi_{R}(\theta )=\frac{1}{N}\sum_{i}\;\frac{\pi_{\theta }\left(a_{i}|s_{i}\right)}{\pi_{\theta_{0}}\left(a_{i}|s_{i}\right)}A_{i}^{0}$, where $\pi_{\theta_{0}}\left(a_{i}|s_{i}\right)$ denotes the probability of taking action $a_{i}$ in state $s_{i}$ and $A_{i}^{0}$ is the advantage of sample i estimated from the buffer rollouts (Appendix I.1). We find maximally different policies by steering the null space walk to maximize the behavioral divergence, defined by the KL divergence between the action distributions of the anchor and the alternative policy on the buffer states: $\phi_{B}(\theta )=\frac{1}{N}\sum_{i=1}^{N}\;D_{KL}\left(\pi_{\theta_{0}}\left(\cdot |s_{i}\right)\|\pi_{\theta }\left(\cdot |s_{i}\right)\right)$. We periodically recollect the state–action buffer from the current policy to account for the changing state distribution, and recompute the surrogate reward and its null space along the walk. Details for continuous and discrete action spaces are provided in Appendix I.2.

We applied HNC on Plume Tracking (Singh et al., 2023) and AI Safety Gridworld (Leike et al., 2017) environment; additional MuJoCo results are provided in Appendix I.7. On the *Plume Tracking* task (Singh et al., 2023) (Fig. 4A), RNNs simulating artificial flies are trained by deep RL to navigate to the source of a turbulent odor plume in a windy 2D arena. Trained artificial flies resemble real flies by surging upwind when they detect odor and casting (sweeping back and forth across the wind) after losing it (Singh et al., 2023) (Fig. 4A). Over the HNC, the action divergence between anchor and the alternative network steadily increases, while the episode return and the success rate (Appendix I.4) are maintained, even increased early in the walk (Fig. 4B). The alternative policy solves the task in a visibly different way: instead of surging straight into the plume, the agent slides to the edge of the plume and smoothly tracks it (Fig. 4A). Behavioral quantification confirms that the alternative policy casts less and on average stays farther from the centerline of the plume (Fig. 4C; details in Appendix I.4). Strikingly, when evaluated on out-of-distribution conditions with sparse odor or switching wind direction, the alternative policy outperforms the anchor (Appendix I.5).

We next ask whether HNC can expose reward-hacking policies near a well-behaved solution in the AI Safety Gridworlds environment (Leike et al., 2017). In the *boat race* task, an agent earns a proxy reward of +3 each time it enters an arrow tile clockwise (Fig. 4D). The true return, which the agent never sees, instead measures net clockwise progress. The proxy therefore admits a known exploit: stepping on and off a single arrow tile collects the proxy reward repeatedly without net progress. We train MLP policies with PPO on the true return, which yields track-following behavior in all 10 seeds (Appendix I.6). Starting from these anchors, we use HNC to search for behaviorally distinct policies while preserving the proxy return. Behavioral divergence increases steadily along every null space walk, and the endpoints separate into two clusters (Fig. 4E). The three most divergent endpoints adopt the reward-hacking strategy, where their net clockwise progress falls to zero. Figure 4D contrasts the state occupancy of an anchor and its HNC endpoint, showing a transition from track-following to oscillation around a single arrow. Overall, these results demonstrate that HNC can find behaviorally distinct yet reward-matched policies nearby in weight space in RL-trained networks, and can expose the underspecification in reward design that admits reward hacking solutions.

## 6 HNC measures loss landscape geometry

Beyond finding alternative solutions, HNC provides two complementary measures of loss-landscape geometry: the Hessian characterizes the local geometry at an anchor, while the null space walk tracks how that geometry changes along a trajectory. We use these measurements to ask how model size and task complexity shape the solution set. Previous work has addressed this question through loss barriers between independently trained networks (Garipov et al., 2018; Draxler et al., 2018; Frankle et al., 2020; Entezari et al., 2022), or through stylized theoretical models (Cooper, 2018; Simsek et al., 2021). HNC instead enables direct measurements from a single trained network. Here, we train CNNs on CIFAR100 subsets while varying network width and the number of classes, our proxy for task complexity (Appendix J.1).

We first estimate the **effective null fraction**, the fraction of parameter directions along which a small step leaves the outputs approximately unchanged. We set the spectral cutoff using a common function-drift tolerance, where a step of 1% of the weight norm along a flat direction raises the function-matching loss in Eq. 1 by at most ϵ=0.05 (Appendix J). This ties the cutoff to an interpretable output-drift tolerance, allowing comparisons across conditions with different overall Hessian scales. The trends below hold across different magnitudes of ϵ (Appendix J.2). The effective null fraction grows with width and shrinks with the number of classes (Fig. 5A). Nevertheless, even the smallest network on the hardest task retains a large effective null fraction. We next ask whether smaller networks and harder tasks instead increase the functional cost of moving along these directions.

We therefore measured the **normalized curvature** along the HNC walk, $d^{⊤}Hd/(nC)$, where d is a unit-norm step direction, n is the number of probe inputs, and C is the number of output classes. This quantity measures how sensitive the outputs are to small weight changes along d. To avoid constraining the output sensitivity, we replaced the output-drift cutoff with a common relative flatness threshold, $\mu =10^{-6}\lambda_{\text{max}}$. Normalized curvature generally increases with task complexity and decreases with width (Fig. 5B), and these trends persist across the relative thresholds tested (Appendix J.3). Although approximately flat directions remain abundant across the sweep, the directions HNC follows become more sensitive to weight changes on harder tasks. This indicates that the landscape around a local minimum stiffens and increases in curvature for harder tasks, thereby providing a potential mechanism for prior work that suggests that the loss landscape fragments into separate basins as tasks get harder (Frankle et al., 2020; Entezari et al., 2022; Simsek et al., 2021).

## 7 Discussion

We introduced Hessian Null Space Continuation (HNC), a scalable, domain-agnostic method for traversing the connected solution space around a trained network while preserving its input-output mapping. Across RNNs, Vision Transformers, and reinforcement-learning policies, HNC uncovered solutions with substantially different representations, dynamics, and behavioral strategies. It also provided local geometric measurements of the solution set, revealing how model size and task complexity shape its dimension and functional sensitivity.

Our geometric results in Section 6 complement Huang et al. (2025), which found that harder tasks reduce variability in neural dynamics across independently trained RNNs. They also found that inter-model variability decreases with model size, whereas our results show that larger models have larger null spaces on the same task. Together, these findings suggest that larger models admit more alternative solutions within a local region of weight space, even as training converges to a more narrow subset of them. This is consistent with the stronger simplicity bias of large models proposed to explain representational convergence across models (Huh et al., 2024). Prior work has shown that parameter symmetries and reparameterizations can also reorganize representations while preserving function (Theiss et al., 2026a;b). However, the diverse solutions found by HNC go beyond reparameterizations of a trained solution: they involve qualitative changes in the network’s dynamical regime, altered out-of-distribution generalization, and qualitatively distinct behavioral strategies. Moreover, whereas the parameter symmetries studied by Theiss et al. (2026a) involve discrete operations that may change the size of the network, such as neuron duplication, HNC makes continuous, small changes to the weights of a trained network to reach distinct solutions.

One limitation of HNC is that function preservation is approximate and enforced on a finite probe set. Small function-matching loss therefore does not guarantee output agreement on unseen inputs, particularly under distribution shift. The diversity accessible to HNC depends on both probe coverage and the allowed output drift, making held-out functional evaluation important when interpreting the resulting alternatives (Appendix F.8, G.5, G.6). HNC is also a local search algorithm, making it agnostic to the existence of multiple solution basins. However, we found that alternating with a global method such as that in Ostrow et al. (2026) enables broader search (Appendix E).

Beyond the input-output mapping, the preserved quantity can be any differentiable function of the network. Replacing the function-matching loss with the task loss would instead allow HNC to explore the broader set of task-compatible networks (Appendix D). The steering objective is equally flexible and could encode performance on a second task to obtain multitask networks or alignment with neural data (Appendix H). The same Hessian spectrum also provides a complementary prescription when the goal is to change the network’s function: flat directions preserve the function, whereas sharp directions are most effective for changing it (Gan & Isola, 2026; Liang et al., 2026).

More broadly, HNC allows us to constructively explore the solution space and compare the particular solution reached by training with the function-preserving alternatives around it. This comparison helps distinguish properties demanded by the task from those specific to an individual training run (D’Amour et al., 2022; Fisher et al., 2019). In this respect, HNC enables empirical studies of the optimizer’s implicit bias and complements approaches that estimate implicit regularization from discrepancies between weight updates and loss gradients (Rudoler et al., 2026) or from the displacement of Hessian eigenvectors during training (Marjankowska et al., 2026). Our method could also be further scaled to large language models, for which second-order information has already been used to improve optimization (Martens & Grosse, 2015; George et al., 2018; Liu et al., 2024; Grosse et al., 2023). Finally, the function-preserving solutions exposed by HNC provide a natural geometric setting for model merging, fine-tuning, and editing (Wortsman et al., 2022; Ilharco et al., 2023; Ainsworth et al., 2023; Meng et al., 2022; Mitchell et al., 2022; Gan & Isola, 2026), as well as for auditing reward specifications by uncovering behaviorally distinct or reward-hacking policies (Amodei et al., 2016; Leike et al., 2017; Skalse et al., 2022).

## Supplementary Material

Supplement 1

## Figures and Tables

**Figure 1:**
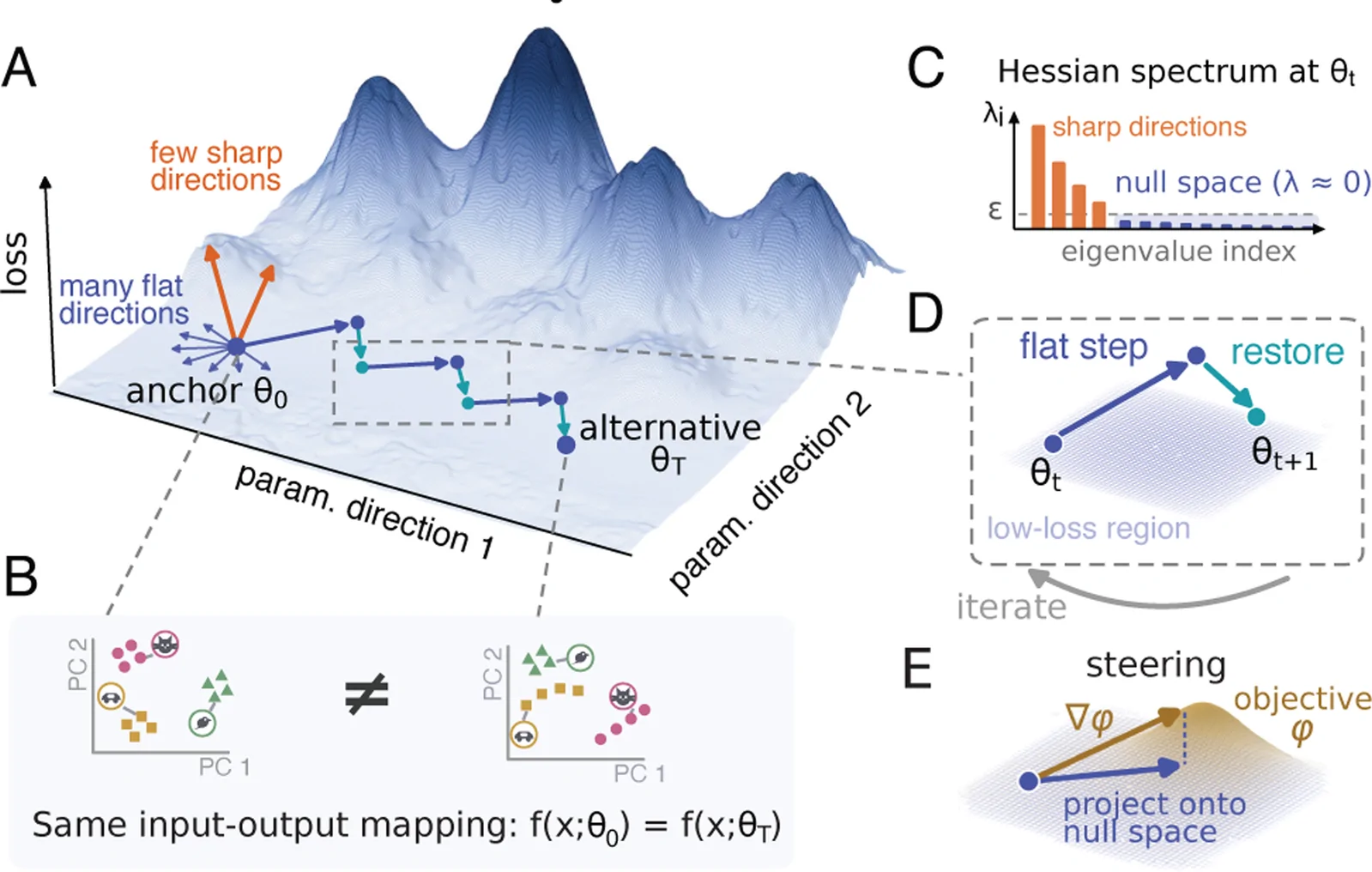
Hessian Null Space Continuation (HNC) overview. **(A)** Around a trained network $\theta_{0}$ (the anchor), the function-matching loss Eq. 1 is sharp along a few directions and flat along many. HNC follows the flat directions to an alternative network $\theta_{T}$ within the connected low-loss region. **(B)** The two networks share the same input-output mapping, but their hidden-state representations of the same inputs can differ. **(C)** The Hessian spectrum at $\theta_{t}$ separates a few sharp directions from a high-dimensional null space with eigenvalues below a threshold ϵ. **(D)** Each iteration takes a step within the null space (flat step), then re-minimizes the function-matching loss (restore), yielding $\theta_{t+1}$. **(E)** The flat step can be steered to optimize a differentiable objective φ by following its gradient projected onto the null space.

**Figure 2:**
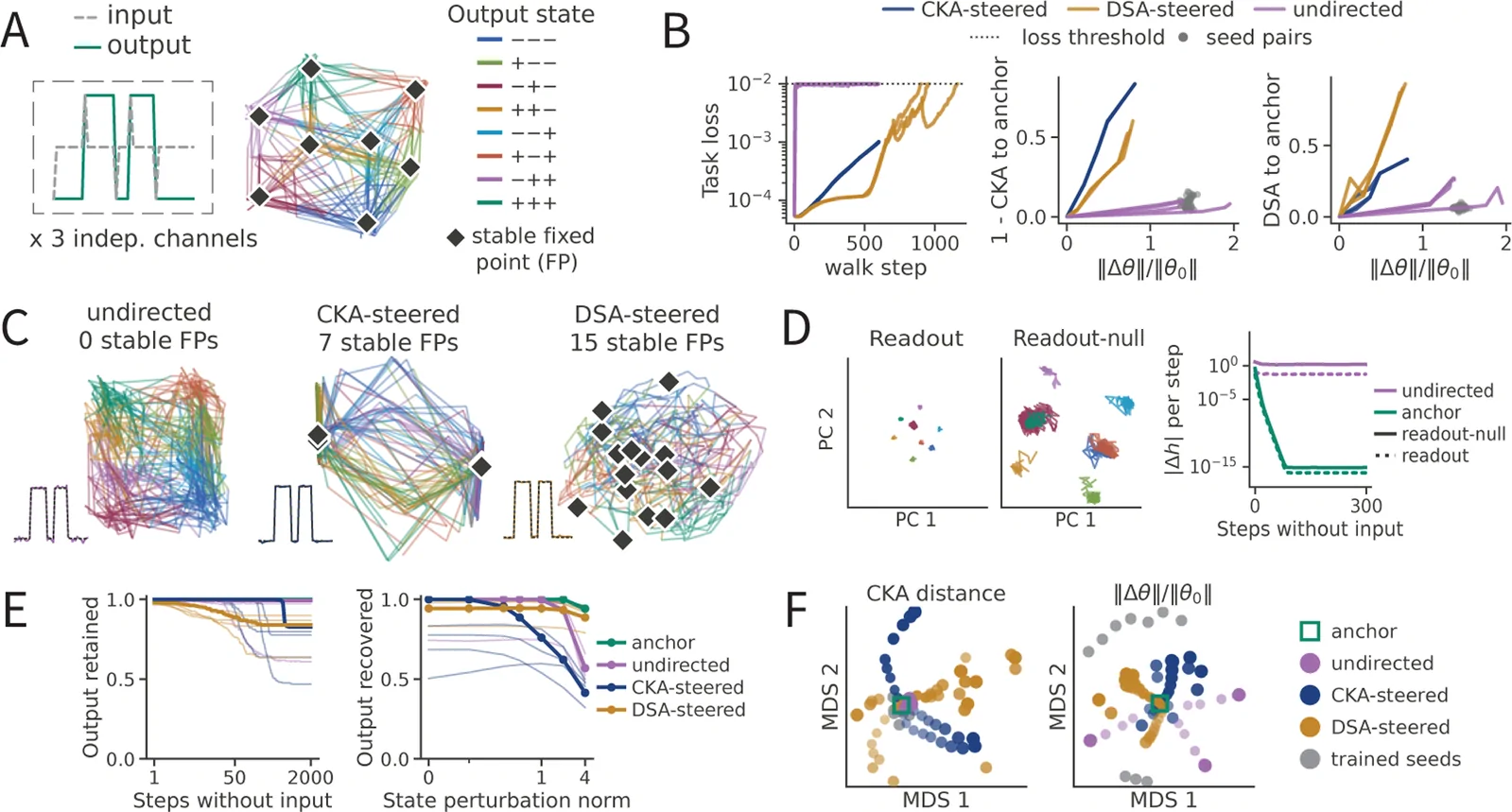
HNC drives RNNs to learn drastically different dynamics in a memory task. **(A)** The 3-bit flip-flop task and the anchor’s solution. Left: one input channel and the corresponding output, where the output holds the last nonzero input. Right: hidden-state trajectories of the trained anchor projected onto their top three principal components, colored by the output state, with the eight stable fixed points (black diamonds) at the vertices of a cube. **(B)** Undirected, CKA-steered, and DSA-steered HNC walks from the same anchor. Left: task loss plotted against the walk step. Middle and right: representational (1 − CKA) and dynamical (DSA) distance to the anchor against relative weight movement. **(C)** Hidden-state trajectory projected onto their top three principles components, and output traces from networks reached during the HNC walks. **(D)** Left and middle: hidden states of the undirected endpoint during input-free memory periods, projected onto the top two PCs of the readout and readout-null subspaces, colored by output state as in (A). Right: per-step hidden-state movement in the readout (dotted) and readout-null (solid) subspaces. **(E)** Fraction of output retained over increasing steps without input (left) and recovered after hidden-state perturbations (right). Thick lines: networks shown in (C); thin lines: endpoints of other independent HNC walks and the other trained anchors. **(F)** MDS embeddings of 131 networks (the anchor, ten independently trained seeds, and all checkpoints from three HNC walks of each type), using pairwise representational distance (1 – CKA, left) or relative weight distance (right). Marker size and opacity increase along each walk.

**Figure 3:**
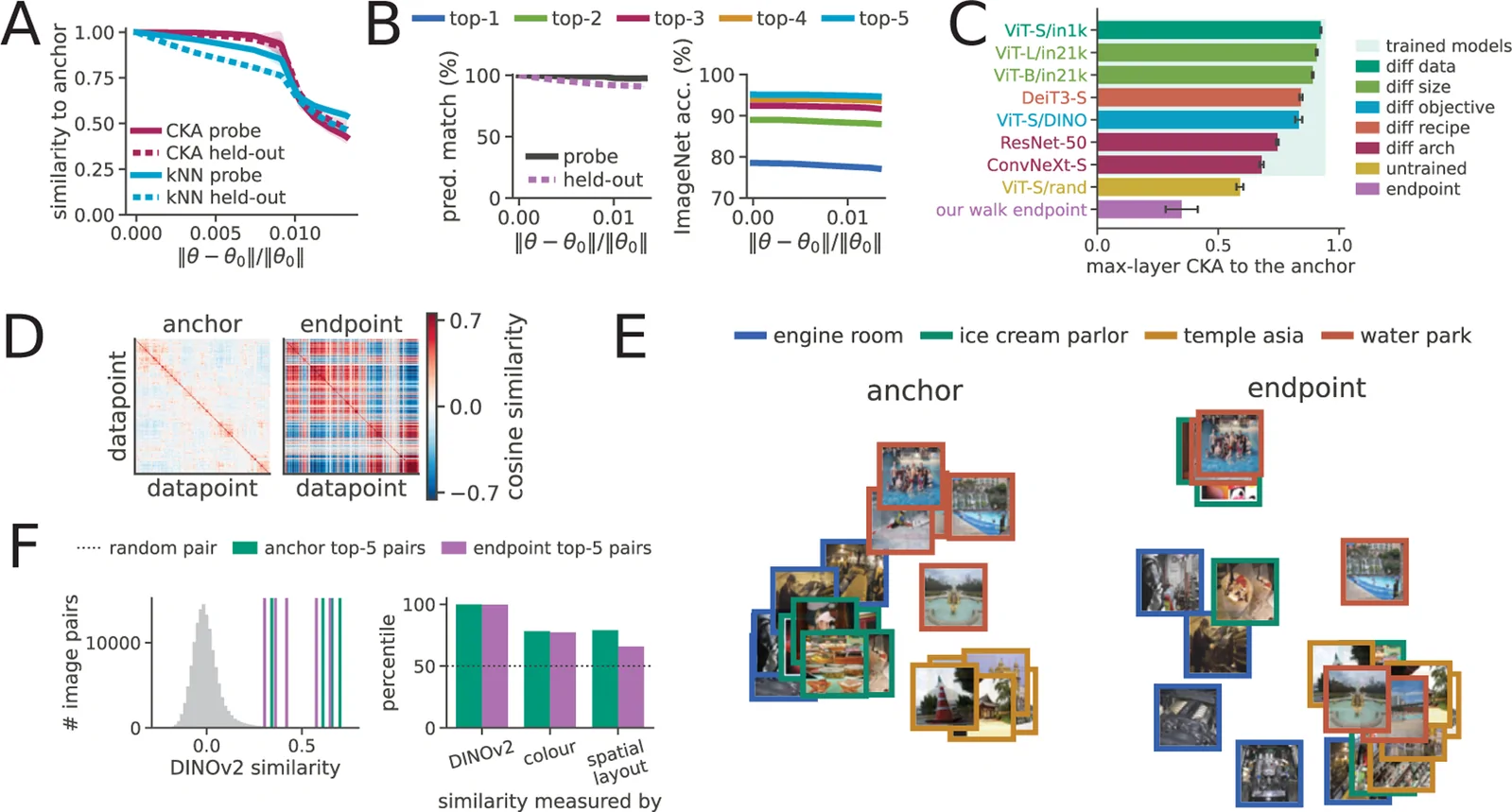
HNC reveals representational freedom underexplored by gradient descent in ViTs. **(A)** Max-over-layers CKA and *k*-nearest-neighbor (kNN) to the anchor along the HNC walk, both on images it was steered on (the probe set) and on images never seen during HNC (the held-out set). **(B)** Left: agreement between the anchor and HNC endpoint’s predictions on Places-365 images. Right: top-1 to top-5 ImageNet accuracy. **(C)** Max-over-layers CKA to the anchor, between our HNC endpoint, an untrained ViT, and seven trained models. In (A-C), lines show the mean and shaded bands indicate ±1 s.d. across five random probe sets. **(D)** Pairwise cosine similarities between penultimate-layer representations of probe set images, for the anchor and one HNC endpoint. **(E)** Multidimensional scaling (MDS) embedding of the similarities in (D) for 20 images from 4 classes. **(F)** For both the anchor and HNC endpoint, we take the five image pairs represented as most similar, and score each pair’s similarity on three attributes: semantic content (DINOv2 feature similarity), color, and spatial layout. Scores are percentiles among all pairs of probe images, where higher means the network’s closest pairs share that attribute more than chance. Dashed line is the level of a random pair.

**Figure 4:**
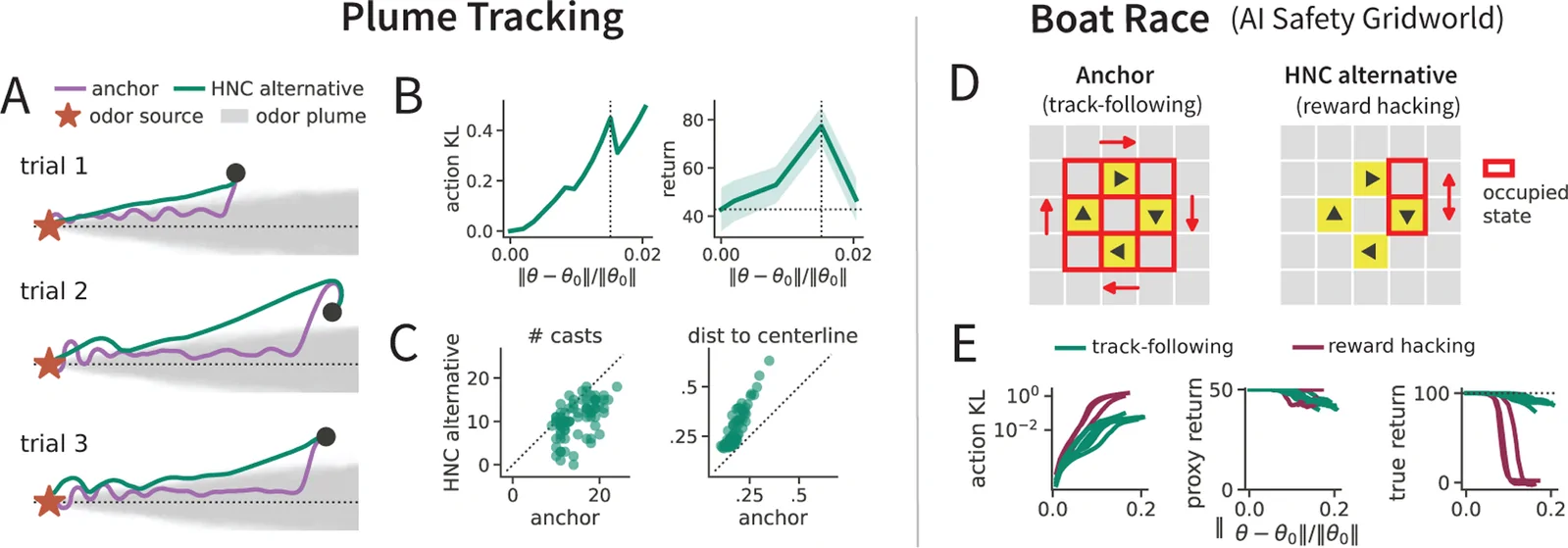
HNC finds behaviorally distinct, high-reward policies in RL. **(A)** *Plume Tracking* task where the agents navigate to the source of an odor plume, which travels away from the source under the dynamics of the wind in the environment. Anchor and alternative policy’s navigation trajectory are shown for three trials. **(B)** Action divergence and return plotted against relative weight change during HNC (dotted vertical line: the policy that we analyze in (A) and (C)). **(C)** Number of casts, defined as the number of times the agent sweeps across the wind and reverses its heading direction, and mean distance from the plume centerline for 240 shared initial conditions. **(D)** *Boat race* task. The proxy reward gives +3 for entering an arrow tile clockwise; the true return measures net clockwise progress around the track but is hidden to the agent during standard training. The anchor follows the track as intended, while HNC exposes a reward-hacking policy that steps on and off a single arrow tile, collecting the proxy reward without making clockwise progress. **(E)** Action divergence, proxy return, and true return plotted against relative weight change during HNC for ten seeds.

**Figure 5:**
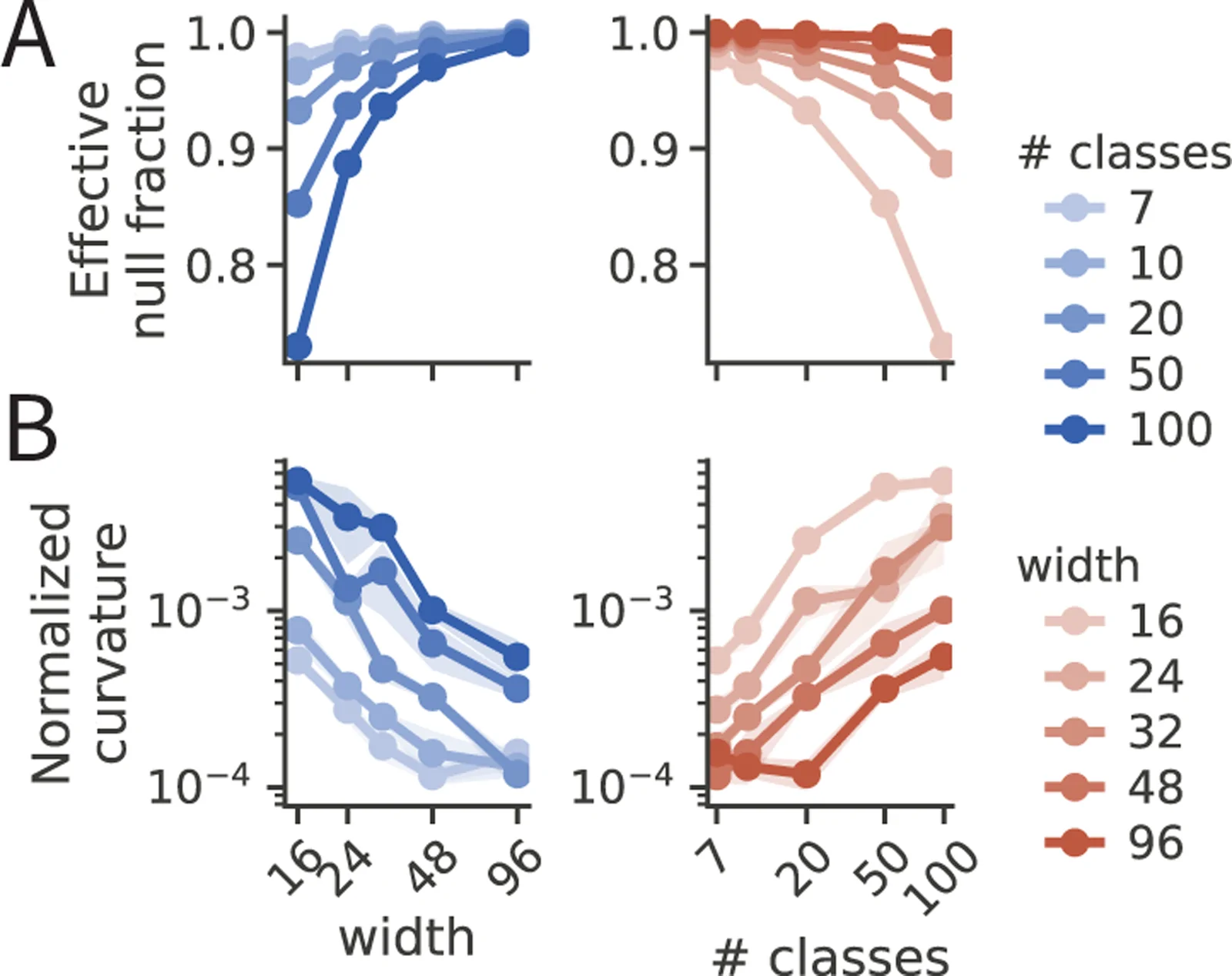
Model size and task difficulty shape local flatness and curvature. **(A)** Effective null fraction as a function of model width and task difficulty (number of image classes). **(B)** Normalized curvature along HNC-stepped directions. Curvature is summarized by the median over steps within each walk. Shading indicates standard error across 3 seeds.
