# Supplementary material for: Traversing the solution space of neural networks with Hessian Null Space Continuation
Source: ArXiv. 2026 Sep 29:arXiv:2609.38081v1. Preprint. [Version 1] (PMC13648821)
Supplement: Supplementary file 1 [file NIHPP2609.38081v1-supplement-1.pdf]

APPENDIX OUTLINE

Method

A Hessian Null-Space Continuation pseudocode . . . 18
A.1 Computational cost . . . 18
A.2 Finding the flattest directions by LOBPCG . . . 19
A.3 Solving the soft projection by conjugate gradients . . . 19
B Gauss–Newton approximation of the Hessian at loss minima . . . 19
C Ablation study on HNC algorithm . . . 20
C.1 Function-restoring step . . . 20
C.2 Null-space projection of the steering gradient . . . 21
C.3 Relinearization . . . 21
C.4 Comparison with penalized optimization . . . 22
D Preserving function versus task loss in HNC . . . 22
E Combining HNC with global search for alternative solutions . . . 26

Experiments

F Details for the 3BFF RNN experiments . . . 29
F.1 Architecture and training of the 3BFF RNN . . . 29
F.2 Size of the flat subspace under different thresholds . . . 29
F.3 Hyperparameters of the null-space walks . . . 30
F.4 A set of diverse 3BFF solutions . . . 30
F.5 MDS embeddings of the solutions reachable from ten anchors . . . 32
F.6 Function-preserving structured pruning . . . 33
F.7 Steered solutions are SGD-stable . . . 34
F.8 Out-of-distribution probes of the walk endpoints . . . 35
G Additional experiments on ViT . . . 36
G.1 Smooth surrogate for the max-over-layers CKA . . . 36
G.2 Scoring the visual attributes of the most similar image pairs . . . 36
G.3 Steering by mutual k-nearest neighbor . . . 36
G.4 Hyperparameters of the ViT walks . . . 38
G.5 Functional preservation beyond the probe set for CKA-steered HNC . . . 39
G.6 Functional preservation beyond the probe set for mutual-kNN-steered HNC 41
G.7 Steering to maximize representational divergence at different layers . . . 43
G.8 Steered convergence between ViT-S and ViT-B . . . 44
G.9 Scaling with the size of the probe set . . . 45
G.10 MDS embeddings of the ViT solutions reached by HNC . . . 46
H Steering Brain-Score at fixed function . . . 46
I Extending HNC to reinforcement learning . . . 49
I.1 Advantage estimates . . . 49
I.2 Behavioral divergence for continuous and discrete action spaces . . . 49
I.3 Hyperparameters of the reinforcement-learning walks . . . 50
I.4 Success rate along the plume-tracking walk . . . 51
I.5 Out-of-distribution evaluation of the plume-tracking HNC walk . . . 51
I.6 The boat race policies . . . 51
I.7 MuJoCo Ant locomotion . . . 52
J Probing loss-landscape geometry with HNC . . . 53
J.1 Architecture and training of the CNN sweep . . . 53
J.2 Sweeping max loss drift to define the null-space . . . 54
J.3 Per-step curvature at different relative thresholds . . . 54
J.4 Representation dimensionality across the sweep . . . 54

A Hessian Null-Space Continuation Pseudocode

After the restore steps, HNC accepts a step only if the loss stays below a ceiling τ. The step-size rule and relinearization schedule are described per experiment (Appendices F.3, G.4 and I.3). Following the discussion in Appendix B, Algorithms 2 and 3 use the Gauss–Newton operator $\mathbf{G} = \boldsymbol{J}^\top \boldsymbol{J}$ in place of the Hessian $\boldsymbol{H}$, because it equals $\boldsymbol{H}$ at the anchor, stays positive semidefinite along the walk, and is cheaper to apply.

Algorithm 1 Hessian null-space continuation (HNC)

Require: trained anchor $\boldsymbol{\theta}_0$, probe set $\boldsymbol{X}$; null-space step size η, restore step size α and number of restore steps m, continuation steps T, null threshold ϵ, block size k, loss ceiling τ; *optional* potential φ with damping μ

$\boldsymbol{\theta} \leftarrow \boldsymbol{\theta}_0$; $\mathcal{L}$ as in Eq. 1; $\mathcal{V}_0 \leftarrow \textsc{NullSpace}(\boldsymbol{\theta}, \epsilon, k)$ ▷ the initial null-space; Alg. 2
for $t = 1, \ldots, T$ **do**
  $\boldsymbol{d} \leftarrow \textsc{StepDir}(\boldsymbol{\theta}, \mathcal{V}_0, \varphi, \mu)$ ▷ step direction; Alg. 3
  $\tilde{\boldsymbol{\theta}} \leftarrow \boldsymbol{\theta} + \eta\, \boldsymbol{d}/\|\boldsymbol{d}\|$ ▷ null-space step
  for $j = 1, \ldots, m$ **do**
    $\tilde{\boldsymbol{\theta}} \leftarrow \tilde{\boldsymbol{\theta}} - \alpha \nabla \mathcal{L}(\tilde{\boldsymbol{\theta}})$ ▷ project back onto $\mathcal{L} \approx 0$
  end for
  if $\mathcal{L}(\tilde{\boldsymbol{\theta}}) > \tau$ **then**
    shrink η; $\mathcal{V}_0 \leftarrow \textsc{NullSpace}(\boldsymbol{\theta}, \epsilon, k)$ ▷ reject: keep $\boldsymbol{\theta}$, relinearize
  else
    $\boldsymbol{\theta} \leftarrow \tilde{\boldsymbol{\theta}}$ ▷ accept
  end if
  relinearize periodically, $\mathcal{V}_0 \leftarrow \textsc{NullSpace}(\boldsymbol{\theta}, \epsilon, k)$ ▷ relinearization schedule
  record $\boldsymbol{\theta}_t \leftarrow \boldsymbol{\theta}$
end for
return a trajectory of function-preserving solutions $\{\boldsymbol{\theta}_t\}_{t=1}^{T}$

Algorithm 2 $\textsc{NullSpace}(\boldsymbol{\theta}, \epsilon, k)$: near-null (flat) subspace of the loss at $\boldsymbol{\theta}$

Require: weights $\boldsymbol{\theta}$, null threshold ϵ, block size k

define the matrix-free Gauss–Newton operator $\mathbf{G} : \boldsymbol{v} \mapsto \boldsymbol{J}^\top(\boldsymbol{J}\boldsymbol{v})$ at $\boldsymbol{\theta}$
$\{(\lambda_i, \boldsymbol{v}_i)\}_{i=1}^{k} \leftarrow \text{LOBPCG}(\mathbf{G}, k)$ ▷ bottom k eigenpairs of the spectrum
return $\mathcal{V}_0 = \text{span}\{\boldsymbol{v}_i : \lambda_i < \epsilon\}$

Algorithm 3 $\textsc{StepDir}(\boldsymbol{\theta}, \mathcal{V}_0, \varphi, \mu)$: null-space step direction inside the flat subspace

Require: weights $\boldsymbol{\theta}$, flat subspace $\mathcal{V}_0$; *optional* potential φ with damping μ

if potential φ is given **then**
  return $(\boldsymbol{I} + \mathbf{G}/\mu)^{-1} \nabla \varphi(\boldsymbol{\theta})$ ▷ *steering*
else
  return any $\boldsymbol{d} \in \mathcal{V}_0$ ▷ undirected exploration
end if

A.1 Computational Cost

HNC is matrix-free and accesses the Hessian $\boldsymbol{H}$ only through Hessian–vector products $\boldsymbol{H}\boldsymbol{v}$, which automatic differentiation computes without explicitly forming $\boldsymbol{H}$ (Pearlmutter, 1994). Each product costs approximately one gradient evaluation and requires memory linear in the number of parameters P. A dense approach instead must store the $P \times P$ Hessian, requiring $\mathcal{O}(P^2)$ memory, and its eigendecomposition costs $\mathcal{O}(P^3)$ time. In contrast, LOBPCG applies $\boldsymbol{H}$ to a block of k vectors and stores only this block. For fixed k and iteration count, its time and memory scale as $\mathcal{O}(kP)$. The dense approach is feasible, and faster due to lower overhead, for the 4,611-parameter 3BFF RNN, where we use it to validate the iterative solver. For the 22-million-parameter ViT-S, however, the dense Hessian alone would require approximately 2 PB of memory, whereas the matrix-free approach remains practical. Table 1 summarizes the computational costs.

Table 1: Cost of a dense eigendecomposition of the Hessian against the matrix-free LOBPCG route used by HNC. P is the number of parameters and k the block size. RNN timings are for the 3BFF anchor of Section 3 on one CPU node with 8 threads; ViT-S timings are from the walks of Section 4 on one NVIDIA H200 with 512 probe images.

	Dense eigendecomposition	Matrix-free LOBPCG
Time	$\mathcal{O}(P^3)$	$\mathcal{O}(kP)$
Memory	$\mathcal{O}(P^2)$	$\mathcal{O}(kP)$
Memory, 3BFF RNN ($P = 4611$)	85 MB	1.2 MB ($k = 64$)
Time, 3BFF RNN	10 s	39 s ($k = 64$)
Memory, ViT-S ($P = 22$M)	1.9 PB	5.6 GB ($k = 64$)
Time, ViT-S	2×10^7 products to assemble ($\sim$months of GPU time), factorization infeasible	≈ 1 s per product; one continuation step ≈ 1 min

A.2 Finding the flattest directions by LOBPCG

Section 2.2.1 needs the k eigenvectors of $\boldsymbol{H}$ with the smallest eigenvalues, which are the directions with flattest curvature. LOBPCG (Knyazev, 2001) finds them by minimizing the Rayleigh quotient

$$\rho(\boldsymbol{v}) \;=\; \frac{\boldsymbol{v}^\top \boldsymbol{H}\boldsymbol{v}}{\boldsymbol{v}^\top \boldsymbol{v}}, \tag{4}$$

which measures the curvature of the function-matching loss along a parameter direction $\boldsymbol{v}$. Its minimum over all $\boldsymbol{v}$ is the smallest eigenvalue, attained at the corresponding eigenvector. LOBPCG minimizes Eq. 4 jointly over a block of k mutually orthogonal directions, which at convergence span the bottom of the spectrum. Each iteration updates the block using the current directions, the residuals $\boldsymbol{H}\boldsymbol{v} - \rho(\boldsymbol{v})\boldsymbol{v}$, and the previous iteration's directions, which is the "locally optimal" part of the method and plays the same role as the momentum term in conjugate gradients. The block is accessed only through the products $\boldsymbol{H}\boldsymbol{v}$, so the solver never explicitly forms $\boldsymbol{H}$. We compute these products by automatic differentiation (Pearlmutter, 1994), at a cost of roughly one gradient evaluation each (Appendix A.1).

A.3 Solving the soft projection by conjugate gradients

The steered direction $\boldsymbol{d} = (\boldsymbol{I} + \mathbf{G}/\mu)^{-1}\nabla\varphi$ of Algorithm 3 is the solution of the linear system $(\boldsymbol{I} + \mathbf{G}/\mu)\,\boldsymbol{d} = \nabla\varphi$. The matrix $\boldsymbol{I} + \mathbf{G}/\mu$ is symmetric positive definite, so we solve the system with the conjugate gradient method (CG; Hestenes & Stiefel, 1952), which touches $\mathbf{G}$ only through products $\mathbf{G}\boldsymbol{v}$. Each product is one forward-mode Jacobian–vector product followed by one reverse-mode product, $\boldsymbol{J}^\top(\boldsymbol{J}\boldsymbol{v})$, and costs about as much as one gradient evaluation, so a solve with n_{CG} iterations costs about n_{CG} gradient evaluations and never forms $\mathbf{G}$. We run a fixed budget of n_{CG} iterations and exit early once the relative residual falls below 10^{-8}. Along a walk the solution changes slowly from step to step, so the solve is warm-started at the previous step's direction. The exception is the 3BFF walks of Fig. 2, which start every solve from zero with a larger iteration budget (Tables 5 and 6). Because the operator is re-formed at the current weights at every step, the steered walks need no separate relinearization.

B Gauss–Newton approximation of the Hessian at loss minima

Our methods reads the flat directions of the loss from a curvature operator that is cheaper than the full Hessian, the Gauss-Newton operator $\mathbf{G} = \boldsymbol{J}^\top\boldsymbol{J}$ which provides close approximation of the full Hessian around loss minimizers.

Write the residual $\boldsymbol{r}(\boldsymbol{\theta}) = f_{\boldsymbol{\theta}}(\boldsymbol{X}) - f_{\boldsymbol{\theta}_0}(\boldsymbol{X})$, so that $\mathcal{L}(\boldsymbol{\theta}) = \frac{1}{2}\|\boldsymbol{r}(\boldsymbol{\theta})\|^2$, and let $\boldsymbol{J} = \partial\boldsymbol{r}/\partial\boldsymbol{\theta} = \partial f_{\boldsymbol{\theta}}(\boldsymbol{X})/\partial\boldsymbol{\theta}$ be its Jacobian (the anchor outputs $f_{\boldsymbol{\theta}_0}(\boldsymbol{X})$ are constant). The gradient is $\nabla\mathcal{L} = \boldsymbol{J}^\top\boldsymbol{r}$, and differentiating once more gives

$$\nabla^2\mathcal{L}(\boldsymbol{\theta}) \;=\; \underbrace{\boldsymbol{J}^\top\boldsymbol{J}}_{\mathbf{G}} + \sum_k r_k(\boldsymbol{\theta})\,\nabla^2 r_k(\boldsymbol{\theta}). \tag{5}$$

The first term is the Gauss–Newton operator; the second is a residual-weighted sum of per-output curvatures. By construction the anchor is a loss minimizer with near-zero residual, $\boldsymbol{r}(\boldsymbol{\theta}_0) =$

$f_{\theta_0}(X) - f_{\theta_0}(X) = \mathbf{0}$, so the second term vanishes and

$$\nabla^2 \mathcal{L}(\boldsymbol{\theta}_0) \;=\; \boldsymbol{J}^\top \boldsymbol{J} \;=\; \mathbf{G}. \tag{6}$$

Because $\mathbf{G} = \boldsymbol{J}^\top \boldsymbol{J}$ is symmetric positive semidefinite, its eigenvalues are real and nonnegative, so the flat (near-null) subspace is unambiguously the bottom of its spectrum, with no spurious negative-curvature directions to disentangle. And $\mathbf{G}$ never has to be formed: a Gauss–Newton–vector product is one Jacobian-vector product followed by one vector-Jacobian product, which is what keeps the null-space computations 1 affordable at scale.

C Ablation study on HNC algorithm

We ablate the main components of HNC using RNNs trained on the 3BFF task (Appendix H.1). Unless stated otherwise, we steer the walks to minimize CKA similarity to the anchor. The ablations address three questions: (1) Do the function-restoring steps correct the higher-order drift accumulated during the null-space walk? (2) Does projecting the steering gradient onto the null-space improve function preservation? (3) Because the null-space is only a local approximation, does relinearization reduce function drift as the walk moves away from the anchor, and how does its frequency affect the outcome?

Finally, we compare HNC with a direct penalty-based alternative that jointly optimizes the steering objective and task loss:

$$\max_{\boldsymbol{\theta}} \; \varphi(\boldsymbol{\theta}) - \beta \mathcal{L}_{\text{task}}(\boldsymbol{\theta}), \tag{7}$$

where β controls the trade-off between divergence and task performance. This comparison tests whether explicitly following the evolving null-space allows HNC to find more dissimilar solutions at a matched task-loss.

C.1 Function-restoring step

After each null-space step, HNC takes m gradient steps on the function-matching loss to pull the network back toward the low-loss region. Figure 6 varies m on an undirected walk at three null-space step sizes η. More function-restoring steps reduce the accumulated function drift and therefore lower the task loss at every step size, with the largest effect at the largest step. At the smallest step size the benefit saturates after a few restore steps, and the walk ends at or below the anchor's loss. At $\eta = 0.3$, twelve restore steps lower the endpoint task loss from 6.6 times the anchor's loss to 3.6 times, with diminishing returns from each additional step. The function-restoring step therefore does not eliminate the drift induced by large null-space steps, and taking smaller steps within the null-space protects the function more effectively than correcting afterwards. The function-restoring step is best thought of as a safeguard that limits drift rather than a projection that removes it, which is why our HNC walks are combined with a small step size, an adaptive step schedule, and a loss ceiling.

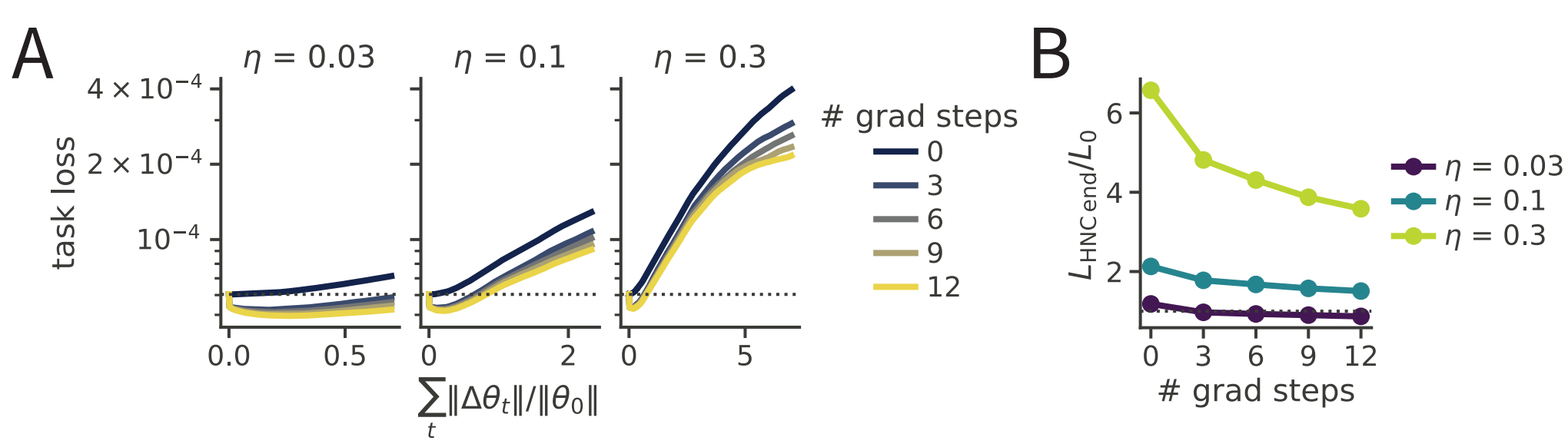

Figure 6: **Ablation experiment on the function-restoring step.** Undirected null-space walks at three null-space step sizes η, with $m \in \{0, 3, 6, 9, 12\}$ restore steps. **(A)** The task loss against the normalized weight travel during HNC walks, where the dotted line is the anchor's loss. **(B)** HNC endpoint's task loss relative to the anchor's against m. Larger null-space steps drift faster, and the restore step corrects for part of the drift rather than eliminating it.

C.2 NULL-SPACE PROJECTION OF THE STEERING GRADIENT

When the walk is steered by a differentiable objective φ, we project its gradient onto the null-space before taking the step. The soft projection is

$$\boldsymbol{d} \;=\; (\boldsymbol{I} + \boldsymbol{H}/\mu)^{-1}\, \nabla\varphi(\boldsymbol{\theta}), \tag{8}$$

where $\mu > 0$ is a damping factor. Along each eigenvector $\boldsymbol{v}_i$ of the Hessian, this projection rescales the gradient component by $1/(1 + \lambda_i/\mu)$, where λ_i is the corresponding eigenvalue. The factor is close to one for flat directions ($\lambda_i \ll \mu$) and close to zero for sharp directions ($\lambda_i \gg \mu$), so the projection keeps the gradient along flat directions and removes it along sharp ones. A larger μ therefore retains the gradient along sharper directions. Figure 7 shows the task loss and the representational divergence ΔCKA along HNC for several values of μ_{rel}, where $\mu_{\text{rel}} = \infty$ denotes no projection at all.

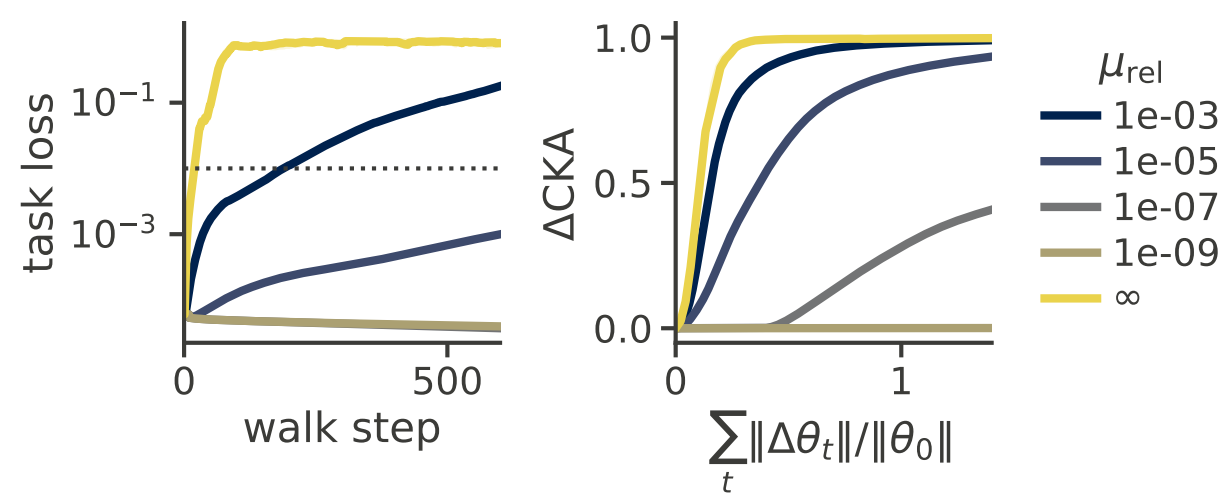

Figure 7: **Ablation experiment on the null-space projection of the steering gradient.** CKA-steered walks from the same 3BFF anchor network at varying magnitudes of damping. Here, the maximum loss threshold is removed so that we can compare the task loss reached during HNC under different damping. A larger damping factor allows the steering gradient along sharper directions to pass through, therefore leading to faster representational divergence at the price of higher task loss. In contrast, with a highly strict damping factor ($\mu_{\text{rel}} = 10^{-9}$), neither the task loss nor the representation changes at all.

A larger damping factor lets the walk diverge faster, but the task loss grows with it. At $\mu_{\text{rel}} = 10^{-7}$ the task loss stays close to the anchor's while ΔCKA rises steadily. Increasing μ_{rel} to 10^{-5} and 10^{-3} speeds up the divergence, at the cost of a clearly higher task loss. With no projection at all ($\mu_{\text{rel}} = \infty$), the walk follows the raw CKA gradient: the steering objective rises fastest of all, but the task loss rises just as rapidly and the function breaks within a few dozen steps, even with the restore step still in place. At the other extreme, the smallest damping factor (10^{-9}) leaves both the task loss and the representation unchanged. The magnitude of μ therefore trades how far the steering objective moves against how much function drifts.

C.3 RELINEARIZATION

The null-space is computed at a given weight configuration, so as the walk moves away from those weights, the curvature along the null-space directions can change and the directions can stop being flat. Relinearization recomputes the null-space at the current weights. To isolate its effect, the walks in Figure 8 use a fixed null-space step size and relinearize on a fixed schedule: never, or every 50, 20, or 5 steps.

More frequent relinearization always lowers the task loss relative to less frequent or no relinearization. When the step size is small, frequent relinearization matters less, but for a large step size it is critical to keep the task loss from blowing up (Figure 8).

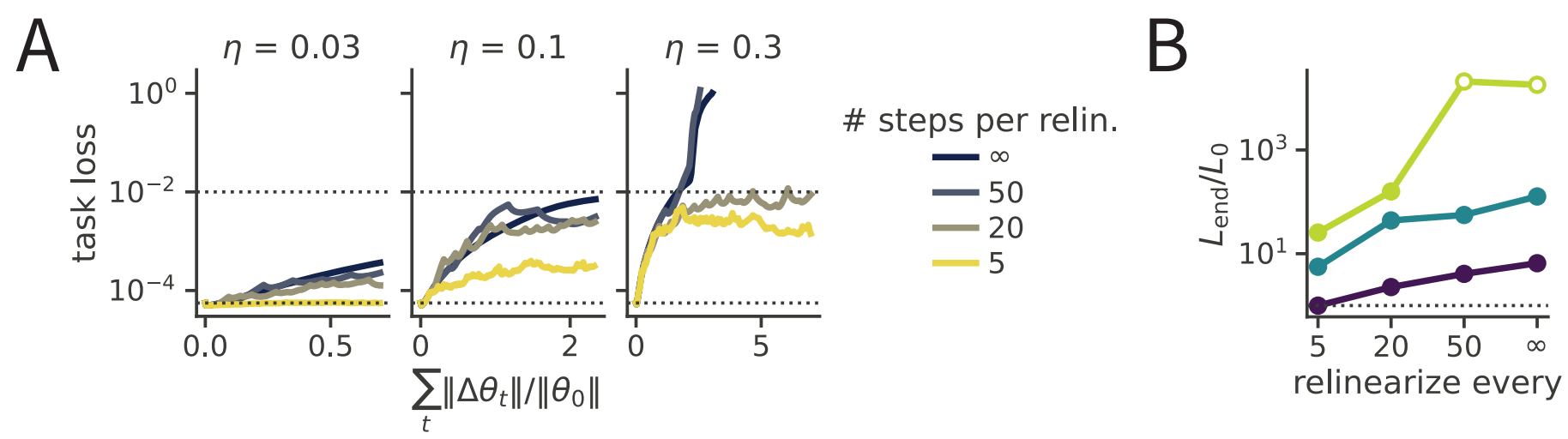

Figure 8: **Ablation experiment on the relinearization step.** **(A)** Constraint task loss against the normalized weight travel; dotted lines mark the anchor's loss and the walk's usual ceiling. **(B)** Endpoint task loss relative to the anchor's against different relinearization interval. Open markers mark walks whose loss blew up.

C.4 Comparison with penalized optimization

The most straightforward way to find a dissimilar solution is to train on the task loss with a penalty on similarity to the anchor, as in Eq. 7. This needs no curvature information: the network follows the combined gradient with steps of the same length as the HNC walk, and the weight β sets how strongly similarity is penalized. We compare this against HNC over the same 600 steps, running HNC at four damping factors and the penalized training at three values of β (Figure 9).

At every level of task loss, HNC reaches a larger divergence from the anchor than penalized training. The two methods also arrive at their divergence in different ways. Penalized training crosses the loss ceiling within its first 20 steps, gains nearly all of its divergence while the task loss is high, and only then returns toward low loss. In comparison, HNC never leaves the low-loss region and gains its divergence steadily.

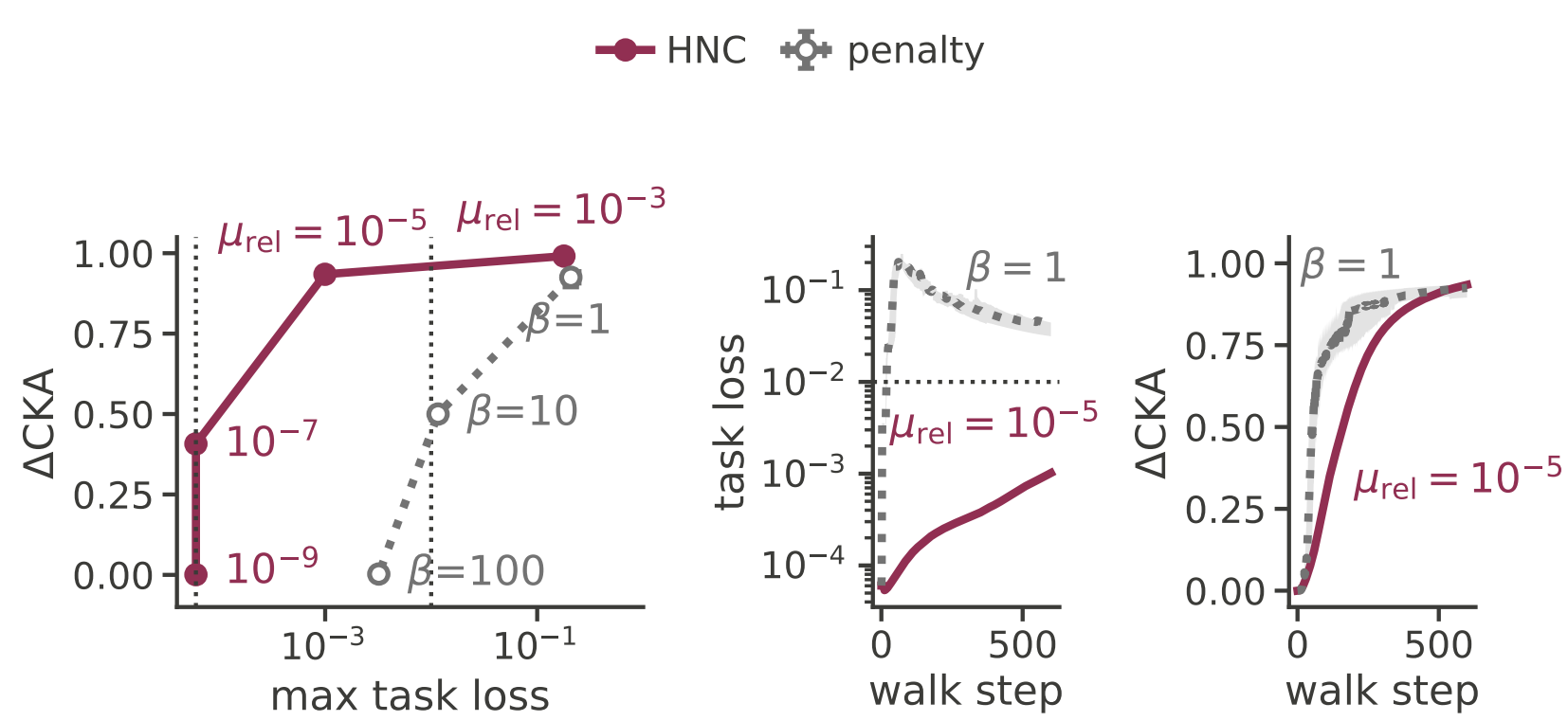

Figure 9: **HNC against training with a similarity penalty.** **Left:** the highest task loss reached during 600 steps against the divergence from the anchor, $\Delta\text{CKA} = 1 - \text{CKA}$, for HNC at four flatness thresholds μ_{rel} and for penalized training at three penalty weights β (five seeds each; markers show the median, bars the range). Dotted lines mark the anchor's loss and the loss ceiling. **Middle, right:** task loss and divergence over the same 600 steps for one HNC walk ($\mu_{\text{rel}} = 10^{-5}$) and one penalized run ($\beta = 1$) that end at similar divergence. At every level of task loss, HNC reaches more divergence. The penalized run crosses the loss ceiling within its first twenty steps, gains nearly all of its divergence during that excursion, and only then returns toward low loss, whereas HNC never leaves the low-loss region.

D Preserving function versus task loss in HNC

HNC holds the function-matching loss of Eq. 1 approximately fixed rather than the task loss on which the network was trained. Preserving the task loss instead would let HNC explore the broader set of task-compatible networks, so here we discuss why we choose to preserve the function-matching loss instead in the paper.

The anchor is an exact minimum of the function-matching loss but only an approximate minimum of the task loss. A flat direction of the Hessian keeps a loss constant only if the gradient of that loss is also zero. Training stops when the task-loss gradient $\boldsymbol{g}$ is small, not when it vanishes, so a step $\eta\boldsymbol{v}$ along a flat direction still raises the task loss by $\eta\,\boldsymbol{g}^\top\boldsymbol{v}$. The null-space step removes the second-order term but still carries the first-order term. Because the gradient term is linear in the step size while the curvature term is quadratic, it is the larger of the two at the small step sizes that HNC takes. Walking a flat subspace of the task loss therefore can drift away from the task the network was trained on, and the drift accumulates step after step. The function-matching loss of Eq. 1 has zero gradient at the anchor by construction, because the anchor reproduces its own outputs exactly. Its flat directions are therefore flat in both first and second order, which is why HNC preserves it rather than the task loss.

The two losses also differ in the signs of their Hessian eigenvalues. The function-matching Hessian at the anchor is positive semidefinite (Appendix B), so its eigenvalues are all nonnegative. Its algebraically smallest eigenvalues are therefore also those closest to zero and identify the flattest directions. On the other hand, the task-loss Hessian contains an additional term:

$$\nabla^2\mathcal{L}_{\text{task}} = \boldsymbol{J}^\top\left(\nabla_f^2\ell\right)\boldsymbol{J} + \sum_k \frac{\partial\ell}{\partial f_k}\,\nabla^2 f_k. \tag{9}$$

The output Hessians $\nabla^2 f_k$ have no sign constraint, so the additional term can outweigh the nonnegative Gauss–Newton term in some directions, giving the task-loss Hessian both positive and negative eigenvalues and making it indefinite. On 3BFF, we find several hundred negative eigenvalues at every trained anchor. Selecting the algebraically smallest eigenvalues therefore favors negative-curvature directions over nearly flat directions, whose eigenvalues are closest to zero.

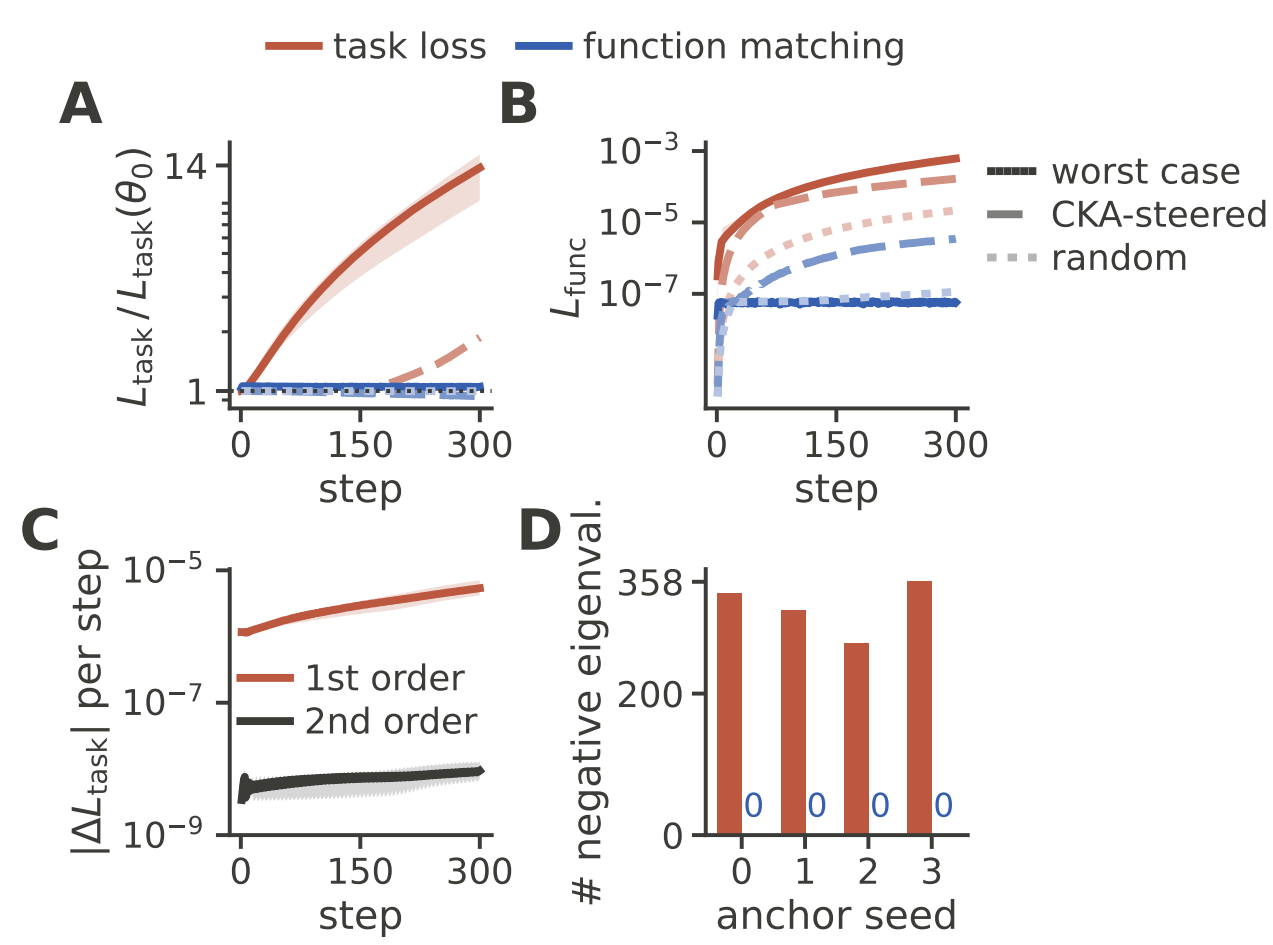

Figure 10: **Task-loss and function-matching walks along the worst-case flat direction on 3BFF. A, B**: task loss relative to its anchor value, and function-matching loss to the outputs of the anchor, over 300 null-space steps from four anchors (median, band from minimum to maximum). Solid lines follow the flat direction along which the task loss rises fastest; the paler dashed lines follow the CKA-steered heading and a random flat direction from one anchor. **(C)**: change of the task loss per step along the task-loss walk, split into the first-order term set by the gradient and the second-order term set by the curvature. **(D)**: number of negative Hessian eigenvalues at each anchor for the two losses. The task-loss walk drifts because the gradient, not the curvature, moves the loss, whereas the function-matching walk holds both losses in place.

We measured these effects on four independently trained 3BFF anchors (seeds 0 to 3 of Appendix F.1), using a dense Hessian and eigendecomposition in double precision so that the sign of every eigenvalue is exact. Two walks start from each anchor and are identical except for the loss that defines the flat subspace and is restored after every null-space step: the task MSE or the function-matching loss. Both walks take 300 null-space steps of the same size along the flat direction in which the task loss rises fastest, the worst case for a task-loss walk, with no loss ceiling so that the drift is visible (Figure 10). At every anchor the task-loss gradient is nonzero, between

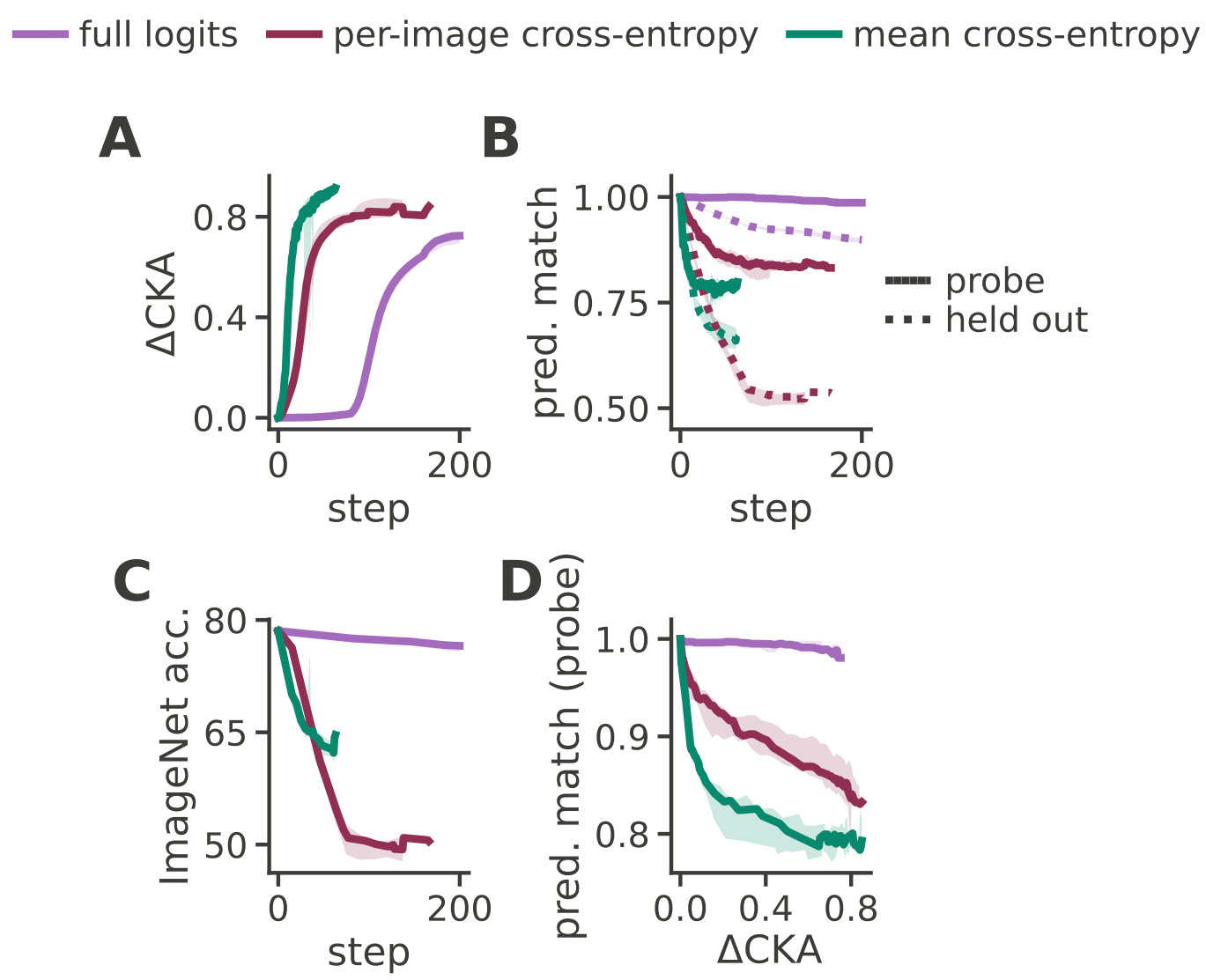

Figure 11: **Constraint strength of three preserved quantities on ViT-S.** CKA-steered walks from one anchor that hold fixed, on a probe of 512 labeled ImageNet validation images, the full logit vector, the per-image cross-entropy, or the mean cross-entropy (median over probe seeds, band from minimum to maximum). **(A)**: representational divergence from the anchor along the walk. **(B)**: fraction of images that keep the top-1 prediction of the anchor, on the probe images (solid) and on $5{,}000$ held-out images (dotted). **(C)**: held-out top-1 accuracy. **(D)**: probe prediction match against divergence, so the walks are compared at matched representational change rather than at matched step. The fewer constraints a quantity imposes per image, the more predictions change, on the probe and beyond it.

2×10^{-3} and 1×10^{-2} in norm, and a small part of it lies inside the flat subspace. The task-loss Hessian has several hundred negative eigenvalues out of $4{,}611$ parameters, whereas the Hessian of the function-matching loss has none. Along the walk, the first-order drift of the task loss per step exceeds its second-order term by two to three orders of magnitude, and the task-loss walk multiplies the task loss roughly tenfold. The function-matching walk keeps the outputs pinned to the anchor and, as a by-product, holds the task loss within ten percent of its anchor value.

On 3BFF the two losses are nevertheless close in practice, because the task target is the full output trajectory and the trained anchor reaches it up to a small residual, so the task MSE is nearly a function-matching loss to a fixed target.

The full output constrains the network more tightly than a scalar loss. Both losses are enforced on a finite probe set, so the question is how well the constraint carries over to inputs outside the probe. Many output vectors share the same loss value on an image, so a network that keeps the anchor's loss on every probe image can still change its prediction on that image, and it is unconstrained on unseen images. Matching the full output vector fixes one number per class for every probe image rather than one number in total, so the probe constraint transfers better beyond the probe.

We tested this on ViT-S with walks from the same anchor, steered by the same max-over-layers CKA objective with the same solver settings as in Section 4, on a probe of 512 labeled ImageNet validation images. The walks differ only in what they hold fixed: the full logits (the function-matching loss), the per-image cross-entropy at its anchor value, or the mean cross-entropy at its anchor value. The two cross-entropy variants are exact level sets and therefore share the zero-gradient property of the previous paragraph. What they lack is constraint strength.

In Figure 11, the logit walk keeps almost every probe prediction and stays within a few points of the accuracy of the anchor, while both cross-entropy walks lose predictions from the first steps and give up far more accuracy. They lose predictions even on the probe images whose loss is pinned, because a walk can trade errors on some probe images for gains on others at constant loss. The walks also diverge at very different rates, so a given step means something different for each: the

cross-entropy walks climb within a few steps and the logit walk moves only later, which is why panel D compares them at matched representational change instead. Off the probe the ordering of the two cross-entropy variants is not stable, because the mean cross-entropy walk moves the network less per unit of representational change and so carries fewer of its probe errors to unseen images.

To quantify the drift, Table 2 counts the constraints each quantity imposes and reports the probe prediction match at three values of divergence. The full logit vector constrains a thousand numbers per image and holds almost every probe prediction at any divergence we reach. The per-image cross-entropy imposes one constraint per image and the mean cross-entropy a single constraint for the whole probe, and both lose probe predictions immediately and keep losing them as the walk proceeds.

Table 2: Preserved quantities on ViT-S, ordered by the number of constraints they impose on a probe of 512 labeled ImageNet validation images over $1{,}000$ classes. Prediction match is the fraction of probe images given the top-1 prediction of the anchor, at three values of representational divergence (max over layers, mean and standard deviation over probe seeds).

		Pred. match (probe) with $\Delta\text{CKA} =$		
Preserved quantity	# constraints	0.2	0.4	0.6
Full logits	512,000	0.996	0.994	0.993
Per-image cross-entropy	512	0.916	0.894	0.875
Mean cross-entropy	1	0.831	0.811	0.793

Function matching needs neither labels nor a task. The function-matching loss requires only the outputs of the anchor, so the same procedure runs on unlabeled probes, on shifted inputs, on policies whose return is flat or non-differentiable (Appendix I), and on language models where the natural target is the next-token distribution. Because any task loss is a function of the outputs, preserving the function preserves every task loss at once, and the function-preserving set is a subset of the task-compatible set.

Nonetheless, someone who wants the broader solution set for a given task can replace Eq. 1 by the task loss, as each caveat above can be mitigated. The first-order drift disappears if the task loss is held at its anchor value through a squared residual, $\frac{1}{2}\big(\mathcal{L}_{\text{task}}(\boldsymbol{\theta}) - \mathcal{L}_{\text{task}}(\boldsymbol{\theta}_0)\big)^2$, which has zero gradient at the anchor by construction. Alternatively the null-space step can be projected orthogonal to the task-loss gradient, or the anchor can be trained closer to convergence before applying HNC. Additionally, the loose constraint can be tightened by constraining more probe inputs, constraining the per-input losses rather than their mean, or adding the decision on each input as a further target. Each of these moves the constraint toward the full output, and in the limit recovers Eq. 1.

E COMBINING HNC WITH GLOBAL SEARCH FOR ALTERNATIVE SOLUTIONS

HNC starts from a single trained network and explores the connected, function-preserving region around it, so it reaches solutions in the local region. Retraining is the opposite kind of search: a fresh run from a new initialization can land anywhere in weight space, but where it lands is not under our control, and independent runs tend to converge on similar solutions. Here we give a proof of principle that the two can be combined. Starting from the anchor, we alternate two stages. A DSA-steered HNC walk first moves the current network along its loss level set while maximizing the summed dynamical distance to every network found so far. A fresh network is then trained from scratch with the same summed distance as a repulsive regularizer, which asks training to land far from all of them. Each stage hands its endpoint to the next as the new anchor or the new set of networks to repel.

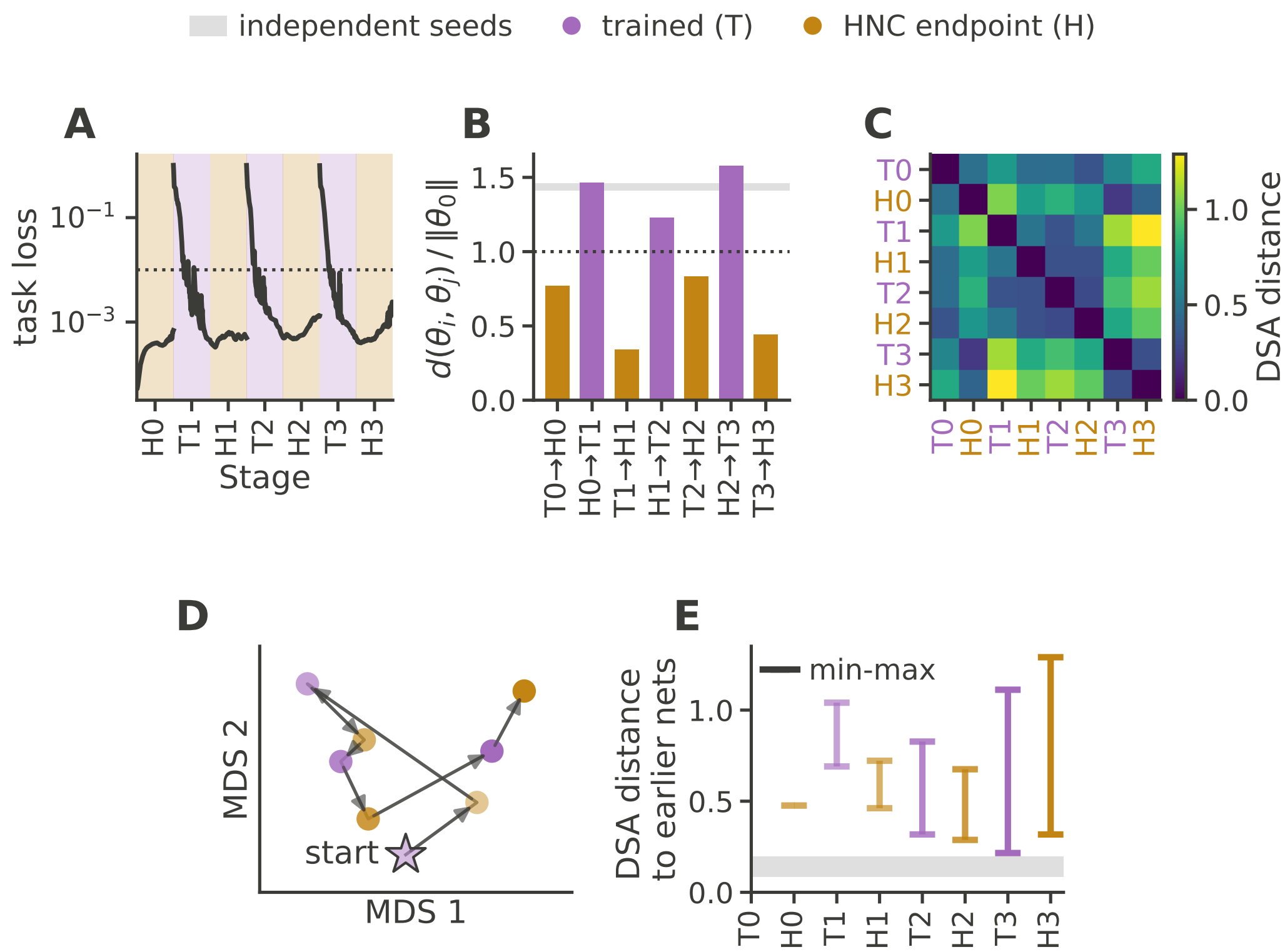

Figure 12: **Alternating retraining with the DSA-steered HNC walk yields a chain of dynamically distinct 3BFF solutions at preserved function.** Starting from the anchor T_0, each round first runs a DSA-steered walk from the current trained network (H_k), then trains a fresh network with a penalty on dynamical similarity to every network found so far (T_{k+1}). **(A)** Held-out task loss along the chain (purple: retraining; orange: HNC walk; dotted line: loss ceiling). Every trained network reaches the anchor's accuracy and every walk stays well below the ceiling. **(B)** Weight distance between consecutive networks, in units of the anchor's weight norm; the grey band is the range of distances between independently trained seeds. Retraining lands as far away as an unrelated seed, whereas a walk moves less than one anchor norm. **(C)** Pairwise DSA distance between the eight networks. **(D)** Two-dimensional embedding of the same distances; the star is the anchor and the arrows follow the chain. **(E)** For each new network, its DSA distance to the nearest and farthest earlier network, against the range between independently trained seeds (grey band). Every network in the chain lies farther from all its predecessors than two seeds lie from each other.

The chain travels farther in weight space than either method alone, and every network in it performs the task as well as the anchor (Figure 12). Each retraining moves about as far as an unrelated seed, and each walk then moves a further fraction of an anchor norm without leaving its loss valley. More

importantly, the chain keeps finding new mechanisms: attractor-based memories, memories held by ongoing drift with no stable fixed point, and a solution with more attractors than memory states (Figure 13), with correspondingly different dynamical spectra (Figure 14). Every new member lies farther from all of its predecessors than two independently trained seeds lie from each other, so alternating global and local search covers more of the solution space than repeated retraining would.

There are other ways to give HNC a more global reach. For example, we can loosen the loss ceiling during the walk and filter the visited networks by task loss afterwards, so the walk could then cross shallow ridges and basin boundaries. The walk could also be restarted from a perturbed copy of its endpoint, a small random kick followed by a few restore steps, to leave a flat sheet whose boundary it has reached. The same regularizer could also repel the alternative networks from solutions found by different architectures or optimizers, and the walk could be steered toward a target network rather than away from the anchor, as in subsection G.8, which would let it bridge two independently found solutions through function-preserving intermediates. We leave these directions to future work.

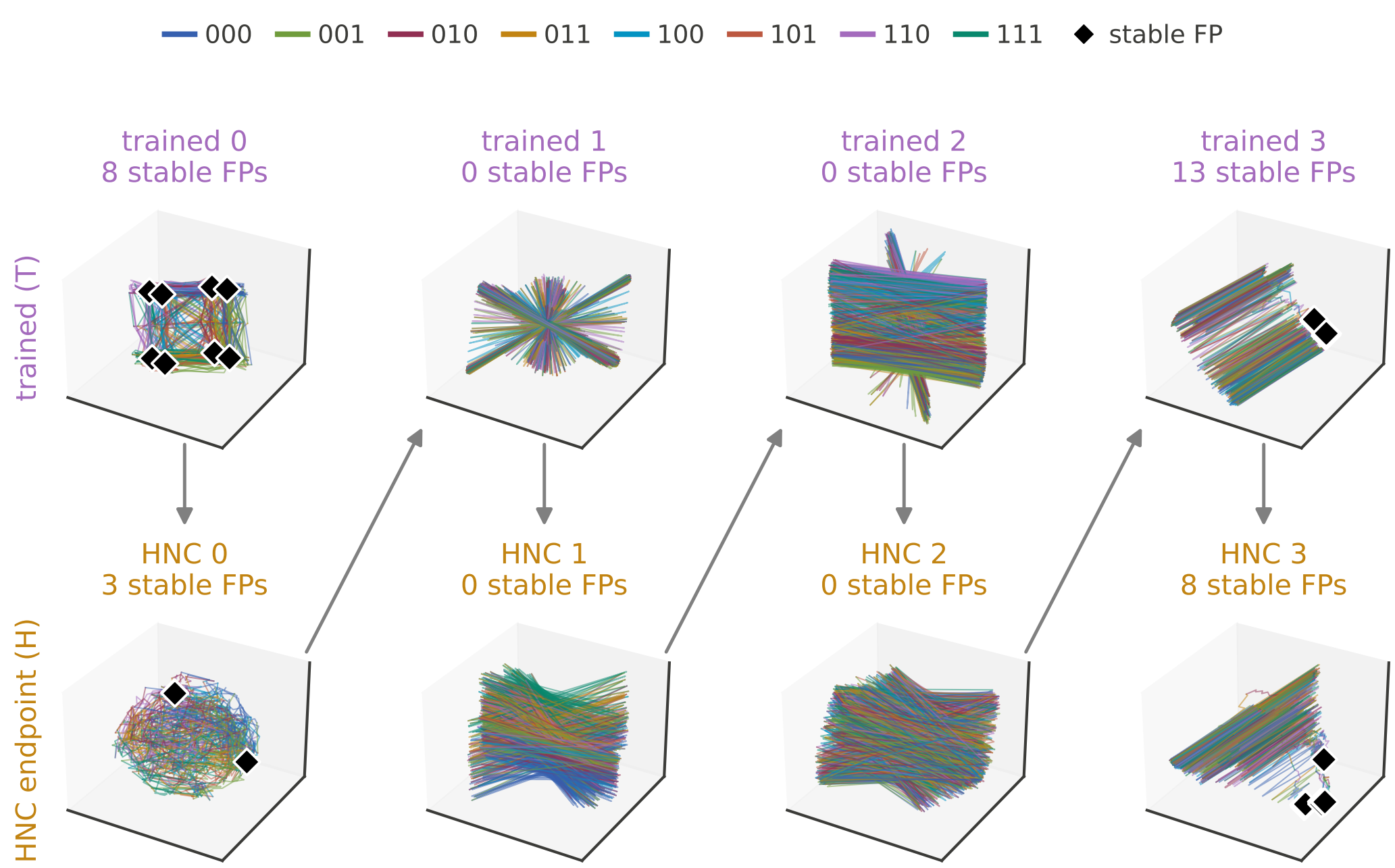

Figure 13: **The chain visits qualitatively different dynamical mechanisms.** Hidden-state trajectories of every network in the chain on the same trials, projected onto each network's top three principal components and coloured by memory state, with stable fixed points as black diamonds. Top row: trained networks; bottom row: the HNC endpoints reached from the network above; grey arrows give the order of the chain. The anchor stores each memory state at its own fixed point. The first walk keeps only a few fixed points and spreads the activity into a cloud, the two retrained networks that follow hold the memory with no stable fixed point at all, and the last round returns to a fixed-point solution with more fixed points than memory states. All eight networks solve the task with bit accuracy above 0.999.

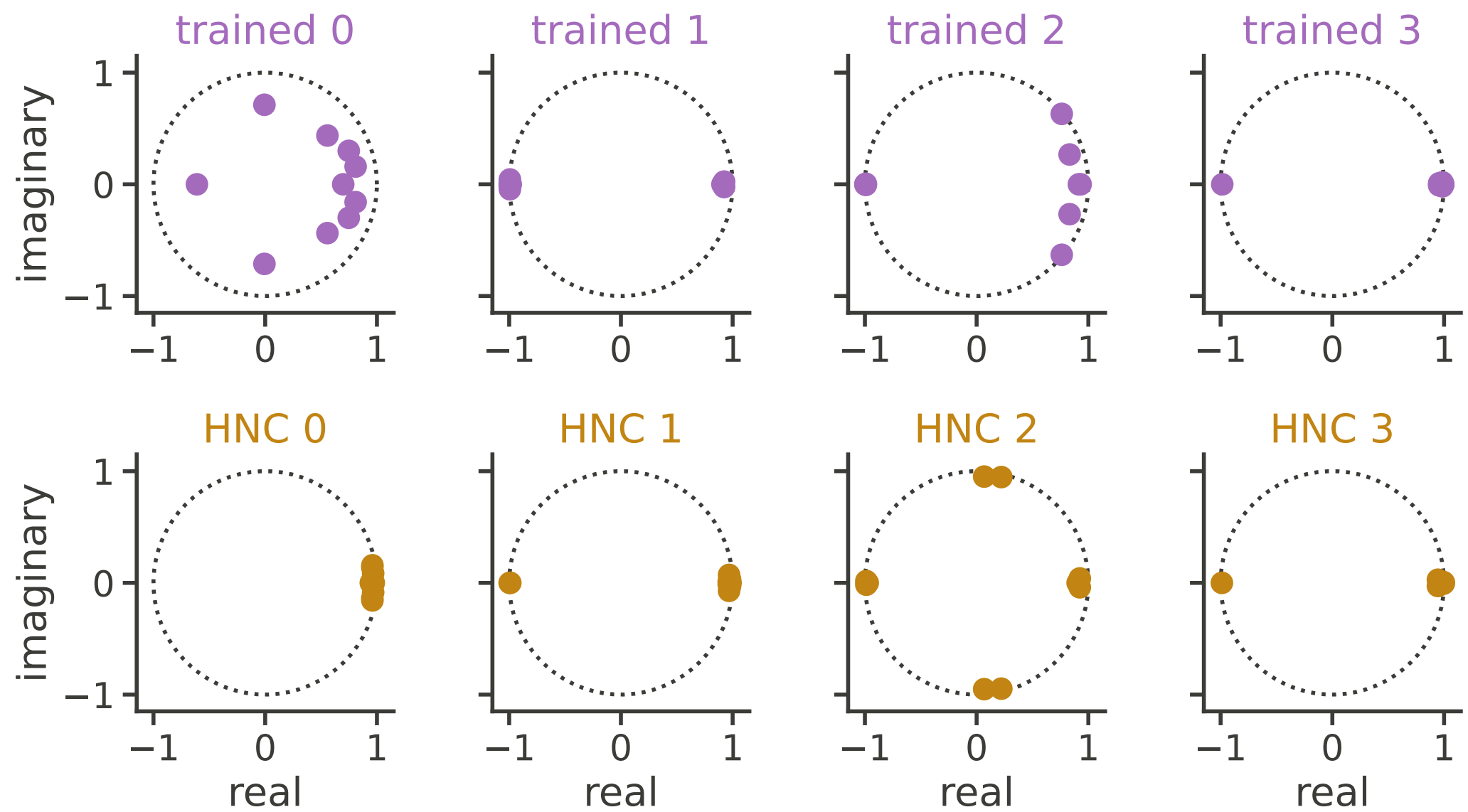

Figure 14: **Eigenvalue spectra of the recurrent dynamics along the chain.** Eigenvalues of a linear model fitted to each network's hidden-state trajectories, with the unit circle for reference; the distance between these spectra is the quantity that both the retraining penalty and the steered walk push apart. The anchor's eigenvalues fan out from the real axis. The networks without fixed points concentrate their eigenvalues at $+1$ and -1, a slowly decaying mode and a mode that alternates every step, and the third walk endpoint adds a pair of modes that repeat every four steps.

F DETAILS FOR THE 3BFF RNN EXPERIMENTS

F.1 ARCHITECTURE AND TRAINING OF THE 3BFF RNN

Task In the 3-bit flip-flop task, each RNN receives N=3 independent input channels taking values in -1, 0, +1, which switch with probability $p_{\text{flip}} = 0.3$. The network has N=3 output channels that must retain the most recent nonzero input on their respective channels. Trials are $T = 100$ timesteps long, and each batch contains 256 trials. Since the three memories are binary and independent. The task has $2^3 = 8$ distinct memory states, which is the origin of the eight-fixed-point cube in Fig. 2.

Architecture We use a vanilla tanh RNN with $N_h = 64$ hidden units, whose update rule is

$$\boldsymbol{h}_t = \tanh\big(\boldsymbol{W}_{\text{ih}}\boldsymbol{u}_t + \boldsymbol{b}_{\text{ih}} + \boldsymbol{W}_{\text{hh}}\boldsymbol{h}_{t-1} + \boldsymbol{b}_{\text{hh}}\big), \qquad \boldsymbol{y}_t = \boldsymbol{W}_{\text{out}}\boldsymbol{h}_t + \boldsymbol{b}_{\text{out}}. \tag{10}$$

We use Kaiming uniform initialization for $\boldsymbol{W}_{\text{ih}}$ and $\boldsymbol{W}_{\text{out}}$, and orthogonal initialization for $\boldsymbol{W}_{\text{hh}}$. All biases are zero. All biases are initialized to zero.

Training Networks are trained by backpropagation through time over all $T = 100$ timesteps on the Mean Squared Error (MSE) loss between the network's output and the target. We use Adam with a constant learning rate of 10^{-3} with the gradient clipped to a global norm of 1.0. Training stops once the epoch loss falls below 5×10^{-5} on two consecutive epochs.

F.2 SIZE OF THE FLAT SUBSPACE UNDER DIFFERENT THRESHOLDS

The 3BFF RNN is small enough ($P = 4611$) that we can form the full Hessian and eigendecompose it directly, which gives the complete spectrum rather than the k flat directions that LOBPCG returns. Figure 15 shows the spectra of the five trained networks. A few directions carry almost all of the curvature: the effective dimensionality of the spectrum, measured by its participation ratio, is 23.2 ± 1.6 out of 4611 (mean $\pm$ s.d. over five anchors). The remaining eigenvalues decay smoothly over nine orders of magnitude with no gap between sharp and flat directions. The size of the flat subspace therefore depends on where the threshold is placed (Figure 15C, Table 3).

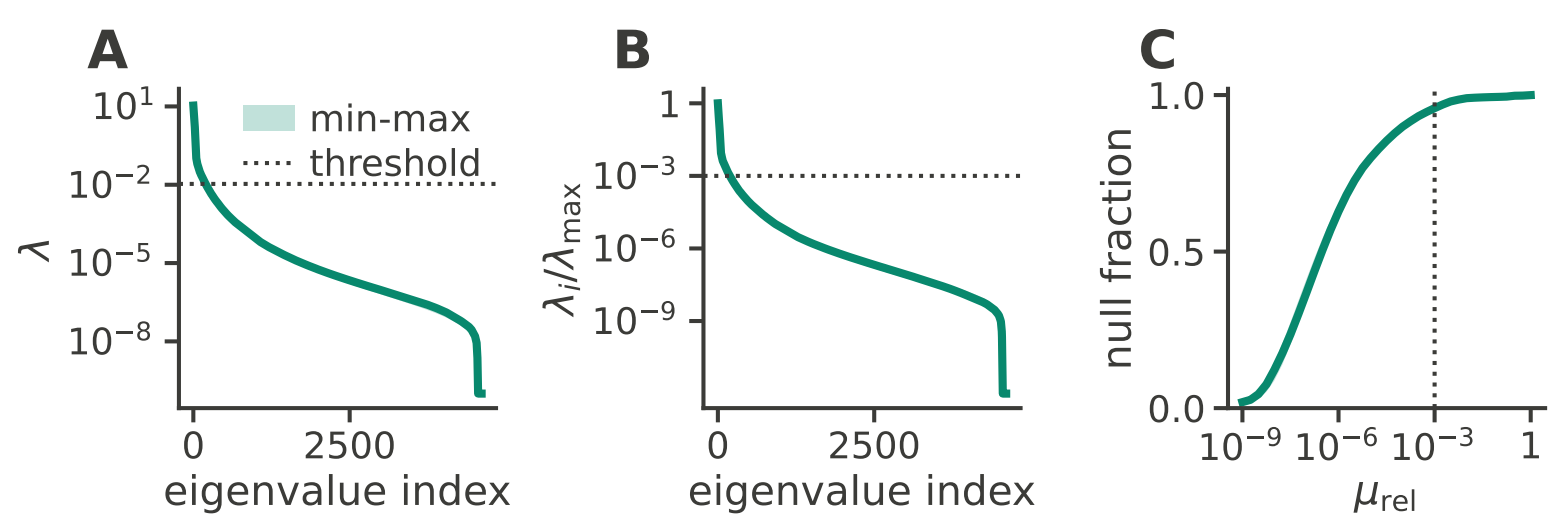

Figure 15: **Hessian spectrum of the trained 3BFF RNNs. (A)** All 4611 eigenvalues at the anchor, sorted. **(B)** The same eigenvalues divided by the largest one. **(C)** Null fraction, the fraction of parameter directions with $\lambda_i < \mu_{\text{rel}}\lambda_{\max}$, as the threshold varies. Curves and bands are the median and range over five independently trained anchors. Dotted lines mark the threshold of the undirected walks, $\mu_{\text{rel}} = 10^{-3}$.

Table 3: Dimension of the flat subspace of the 3BFF RNN ($P = 4611$) as the relative flatness threshold μ_{rel} varies. A direction is counted as flat when its eigenvalue satisfies $\lambda < \mu_{\text{rel}}\lambda_1$, where λ_1 is the top eigenvalue of the Hessian (median 10.8 across seeds).

μ_{rel}	absolute flatness threshold	median dimension	fraction of P	max $\Delta\mathcal{L}$
1.0×10^{-7}	1.1×10^{-6}	1701	36.9%	5.4×10^{-7}
1.0×10^{-6}	1.1×10^{-5}	2896	62.8%	5.4×10^{-6}
1.0×10^{-5}	1.1×10^{-4}	3684	79.9%	5.4×10^{-5}
1.0×10^{-4}	1.1×10^{-3}	4145	89.9%	5.4×10^{-4}
1.0×10^{-3}	1.1×10^{-2}	4418	95.8%	5.4×10^{-3}
1.0×10^{-2}	1.1×10^{-1}	4565	99.0%	5.4×10^{-2}

F.3 Hyperparameters of the null-space walks

Tables 4 to 6 list the settings of the walks in Fig. 2. Every walk starts from one of five independently trained anchors (seeds 0 to 4 of subsection F.1), with three replicates per arm and anchor. The task MSE is measured on a fixed batch of 128 trials. After each null-space step and its restore steps, the step is accepted if this loss is below the loss ceiling and rejected otherwise. A rejected step is undone and the step size halved; after an accepted step the step size grows again. The undirected walks follow an explicit basis of the flattest eigenvectors, which they recompute on a fixed schedule and after every rejection. The steered walks never form a basis. They project the gradient of the steering objective with the soft projection of Section 2, solved at the current weights at every step, so the flat directions are refreshed automatically and no separate relinearization is needed.

Table 4: Settings of the undirected walks in Fig. 2.

Hyperparameter	Value
Flat directions	explicit basis of eigenvectors with $\lambda < 10^{-3}\lambda_1$
Step direction	one random heading direction uniformly sampled from $\mathcal{V}_0$, held fixed and re-projected onto the current basis at every relinearization
Restore steps	12 per null-space step
Null-space step size η	starts at 1.0, halved on rejection and grown $1.1\times$ on acceptance of a step; lower bounded by 10^{-4} and upper bounded by 4.0
Loss ceiling	10^{-2}
Relinearization	every 20 accepted steps or on rejection, at most 60 times
Walk length	600 steps

Table 5: Settings of the CKA-steered walks in Fig. 2.

Hyperparameter	Value
Flat directions	soft projection with damping $\mu = 10^{-7}\lambda_1$
Step direction	gradient of the steering objective, after a seeded random first step
Steering objective	$1 -$ CKA between the hidden states of the current network and the anchor, on a probe of 32 trials
Solver	plain CG, 200 iterations, started from zero at every step (Appendix A.3)
Restore steps	12 per null-space step
Null-space step size η	starts at 0.03; halved on rejection down to 5×10^{-4}
Loss ceiling	10^{-2}
Relinearization	at every step
Walk length	600 steps

F.4 A set of diverse 3BFF solutions

Here, we show a set of diverse solutions that all complete the task with 100% accuracy whose task MSE lies well below 0.05 over an example CKA-steered, DSA-steered, and undirected null-space walk.

Table 6: Settings of the DSA-steered walks in Fig. 2.

Hyperparameter	Value
Flat directions	soft projection with damping $\mu = 10^{-7}\lambda_1$
Step direction	gradient of the steering objective, after a seeded random first step
Steering objective	DSA distance between the hidden-state dynamics of the current network and the anchor (5 delays, rank 10), on a probe of 32 trials
Solver	plain CG, 200 iterations, started from zero at every step (Appendix A.3)
Restore steps	12 per null-space step
Null-space step size η	starts at 0.03; halved on rejection down to 5×10^{-4}
Loss ceiling	10^{-2}
Relinearization	at every step
Walk length	2,000 steps

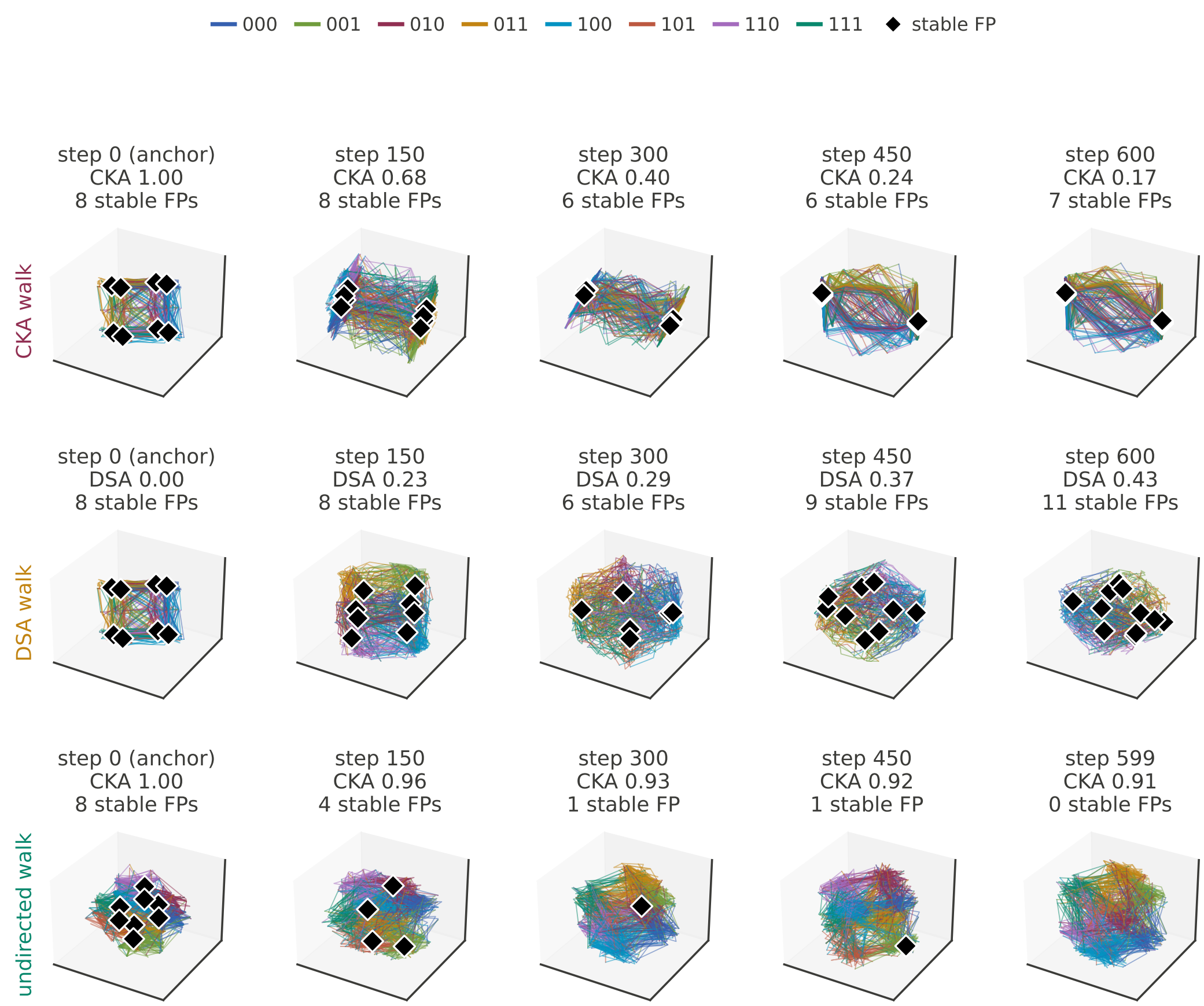

Figure 16: **A set of diverse 3BFF solutions produced by the function-preserving null-space walks.** Hidden-state trajectories on the probe trials, projected onto each snapshot's own top three activity PCs and coloured by the current memory state, with the snapshot's stable zero-input fixed points as black diamonds.

F.5 MDS EMBEDDINGS OF THE SOLUTIONS REACHABLE FROM TEN ANCHORS

Fig. 2F embeds the solutions reachable from one anchor, here we aggregate all networks reached during the HNC walks starting from 10 anchors to highlight the diversity of solutions that a task admits. We trained ten anchors with the training procedure of Appendix F.1. From each of the ten anchors, we first ran 3 undirected, DSA-steered, and CKA-steered HNC walks, resulting in a total of 9 HNC walks, and saving evenly spaced checkpoints per walk. From five of the anchors, we additionally ran walks steered away from an earlier HNC endpoint instead of the anchor, as in Appendix E.

For every pair of networks we computed the CKA distance between hidden states on a common set of probe trials and the Euclidean weight distance relative to the anchor norm, and embedded each distance matrix in two dimensions with metric MDS (Figure 17).

The structure of this solution clearly decouples at the representational level and at the weight level. In weight space the networks group by anchor. Each anchor sits at the centre of its own walks, and no walk comes closer to another anchor than to its own. In representation space, all ten anchors are close to each other, and the steered walks leave them in many directions, reaching distances several times the spread of independent training. The solutions reached by HNC spread across the embedding rather than collapsing into a tight cluster, so the 3BFF task admits far more solutions than independent training reveals.

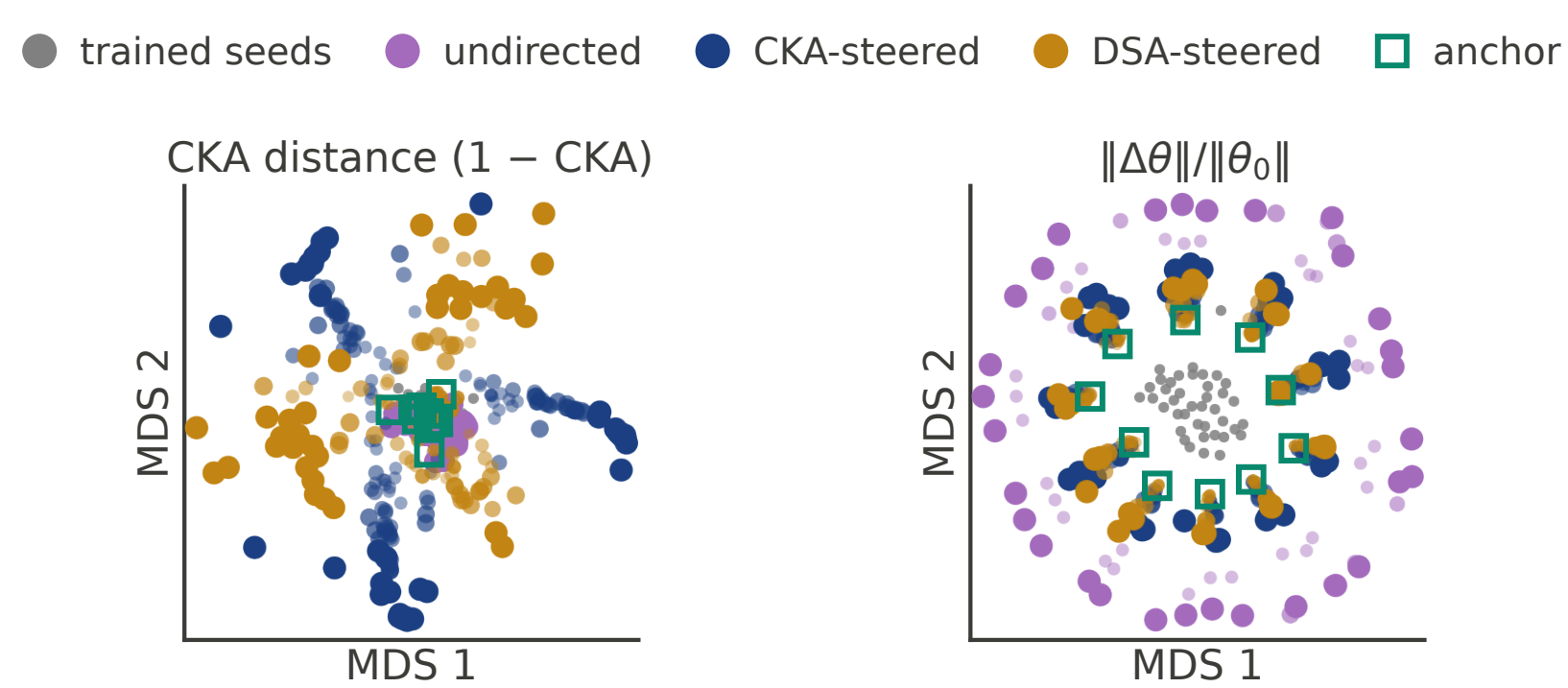

Figure 17: **MDS embeddings of the solutions reachable from ten anchors, by representation and by weights.** Metric MDS of 470 networks: ten independently trained anchors (hollow squares), four checkpoints from each of their undirected, CKA-steered, and DSA-steered walks, and forty further independently trained networks (grey). Walks steered away from an earlier endpoint rather than from the anchor share the colour of the metric they steer. Marker size and opacity grow along each walk. Left: representational distance $(1 - \mathrm{CKA})$. Right: relative weight distance. In weights every anchor forms its own cluster with its walks around it. In representation all anchors and trained networks collapse onto one point, and the walks from every anchor spread far beyond it.

F.6 FUNCTION-PRESERVING STRUCTURED PRUNING

Beyond representational and dynamical dissimilarity, the steering potential can also target the structure of the weights themselves. Here we steer the walk to maximize sparsity of the network weights so to enable network pruning. In the RNN, a unit whose overall output weight magnitude is zero affects neither the recurrent dynamics nor the output, so it can be removed without affecting the network computation. Let unit j's outgoing weight group be $\boldsymbol{g}_j = [\, \boldsymbol{W}_{\text{hh}}[:, j]\, ;\, \boldsymbol{W}_{\text{out}}[:, j]\,]$, we want to steer the walk to maximize a differentiable count of the total number of removable units,

$$\varphi_{\text{prune}}(\boldsymbol{\theta}) \;=\; \frac{1}{N_h} \sum_{j=1}^{N_h} \exp\!\Big(-\tfrac{\|\boldsymbol{g}_j\|^2}{2\sigma^2}\Big), \tag{11}$$

where each unit contributes a Gaussian bump that equals one when its outgoing group is zero and vanishes when the group is large. Because all outgoing weights of a unit share a single bump, the gradient of φ_{prune} drives all output weights of a single unit toward zero together.

Starting from the seed-0 anchor, we apply HNC with an annealed σ schedule, gradually decreasing σ from the median norm of the anchor's outgoing weight groups to one-tenth of that value. This causes the walk to initially shrink all outgoing weights uniformly, before increasingly targeting low-magnitude groups and driving them effectively to zero. Throughout the walk, the fraction of removable units steadily increases (Figure 18A). Physically removing the units with near-zero outgoing weights prunes the 64-unit RNN down to 11 units, about 17% of the original hidden size and 5% of the original parameters. The truncation leads to a small rise in the task MSE, yet the pruned network still performs the 3BFF task with 100% accuracy (Figure 18B, C). This suggests that HNC can directly find and exploit the unconstrained degrees of freedom in the network weights, enabling network pruning.

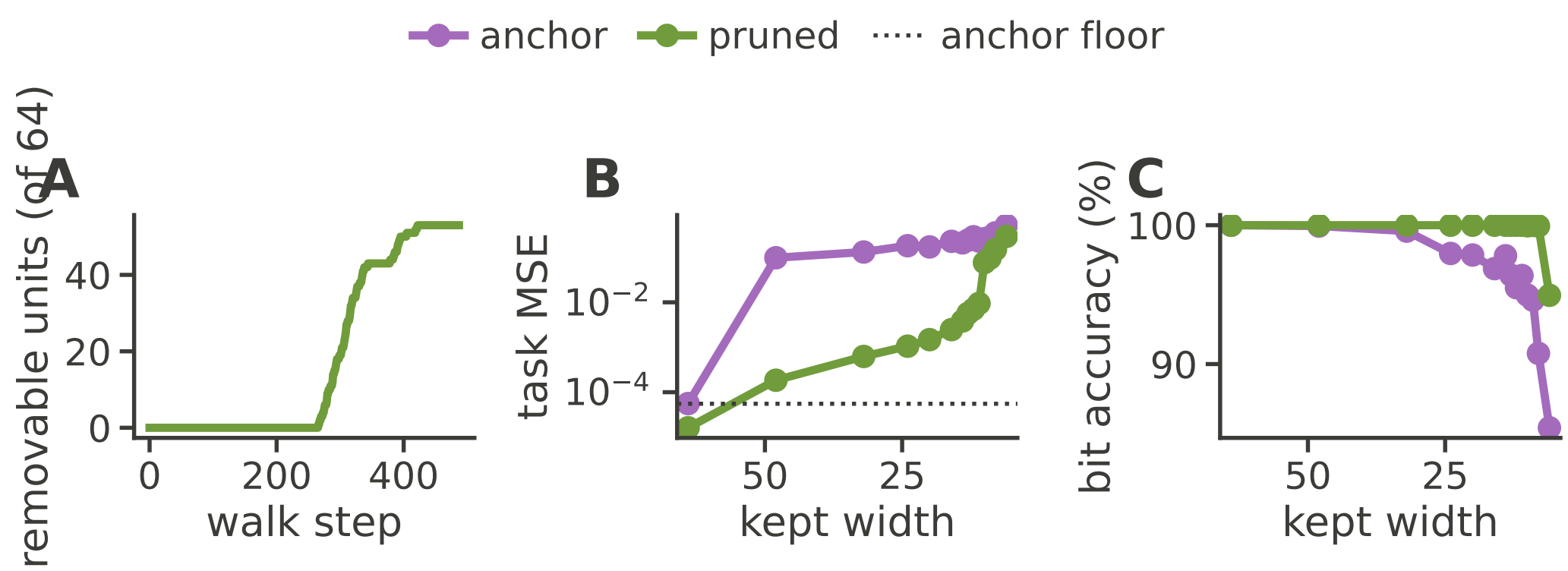

Figure 18: **The prune-steered walk makes the network hard-prunable at preserved function.** **(A)** Fraction of removable units (outgoing group norm below the dead threshold) against the normalized weight movement, the walk arclength divided by the anchor weight norm. **(B)** Hard-truncation test: the RNN is physically rebuilt keeping only the units with the largest outgoing group norms; task MSE against the removed fraction, for the anchor and the prune-steered endpoint. The dotted line marks the largest removed fraction at which the pruned network is still bit-perfect ($53/64 = 0.83$, 11 units kept). **(C)** Bit accuracy of the same truncations: the pruned endpoint stays at 100% down to 11 units, while the anchor is already imperfect at the first truncation and degrades from there.

F.7 STEERED SOLUTIONS ARE SGD-STABLE

Next, we ask whether the solution originally found by gradient descent is privileged for the 3BFF task, such that retraining with SGD would converge back to it. We therefore resume SGD on the 3BFF task for 8,000 steps from each HNC endpoint and, as references, from the anchor and from a fresh random initialization (Figure 19).

Upon retraining, the optimization settles into a solution in the vicinity of each steered endpoint. The property that each walk was steered by (the CKA distance to the anchor, the DSA distance to the anchor, or the fraction of removable W_{hh} columns) stays where the walk left it. Moreover, retraining moves the network weights only a small distance, far less than the distance traveled from a random initialization. The retrained solutions come no closer to the anchor than the steered endpoints they started from either, so retraining does not pull the endpoints back toward the anchor in the weight space (Figure 20).

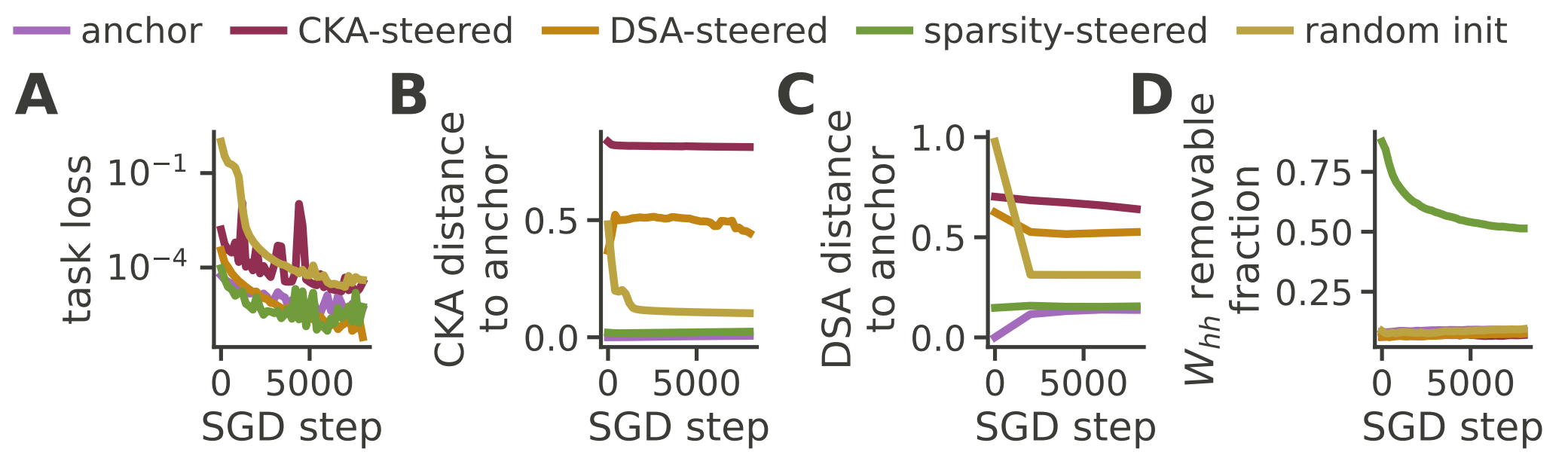

Figure 19: **Resumed SGD maintains the steered divergence while finding a lower-loss solution.** Plain SGD resumed from each steered 3BFF endpoint (and from the anchor and a random initialization), tracking the task loss and the quantities the steering moved: representation (CKA distance to the anchor), dynamics (DSA distance to the anchor), and structure (fraction of near-zero W_{hh} columns).

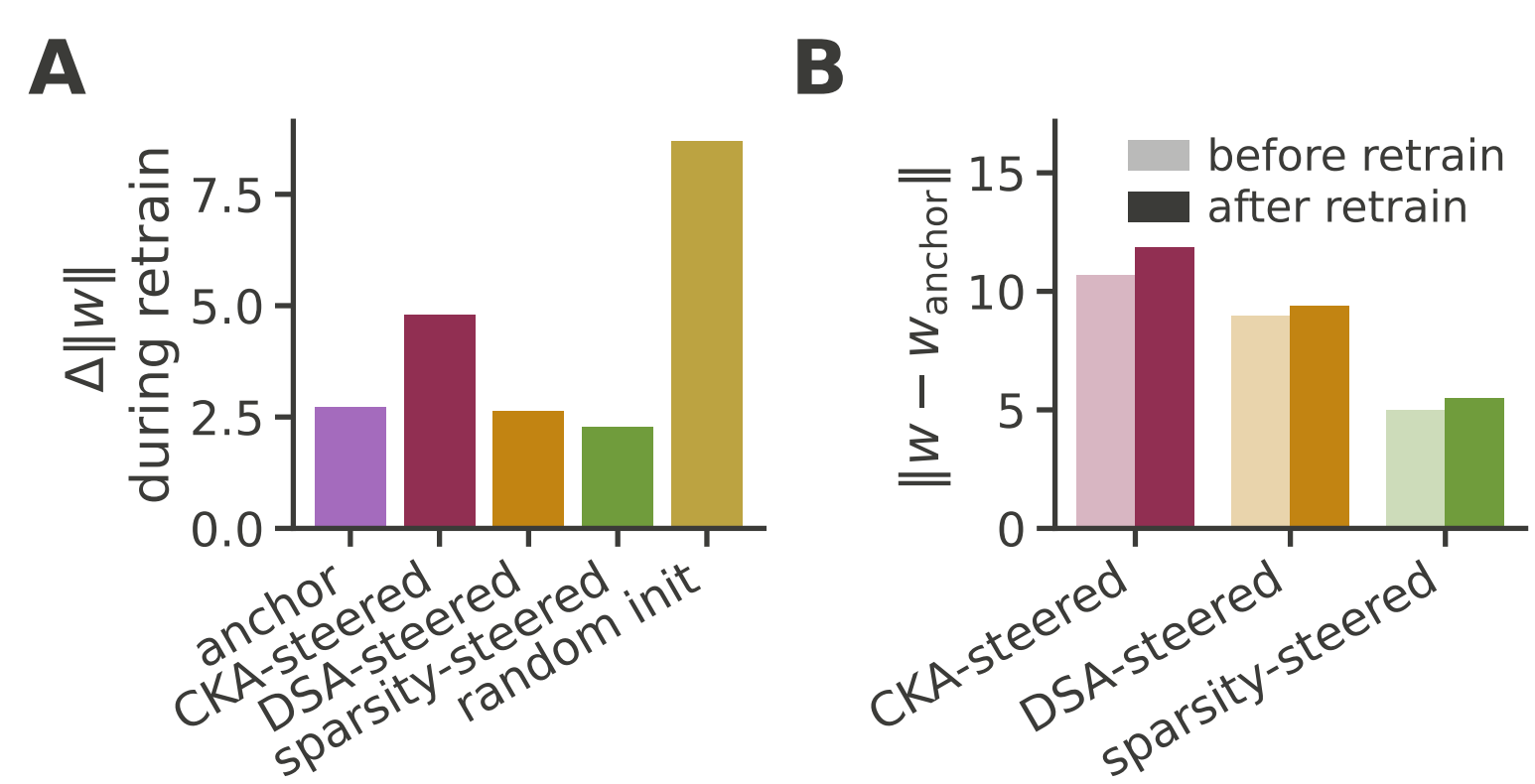

Figure 20: **SGD stays local. (A)** How far the weights move during the resumed training, from each start. **(B)** Distance of each steered solution to the anchor before (light) and after (solid) retraining.

F.8 Out-of-distribution probes of the walk endpoints

The walk endpoints match the anchor on the trained input distribution, so we ask whether they also match it on inputs that this distribution never contains. During training, each input bit receives a unit pulse at every step with probability 0.3. We therefore vary the pulse magnitude and the pulse rate, and we evaluate the anchor and the three walk endpoints, all of which reach an in-distribution accuracy of 0.99 or above (Figure 21). Memory retention and recovery from hidden-state perturbations are shown in Fig. 2E.

Weak pulses degrade the DSA-steered and undirected endpoints before the anchor. Strong pulses degrade only the undirected endpoint, whose recurrent spectral radius has grown along the walk. Rare pulses leave long stretches without input within a trial. There the DSA-steered endpoint loses accuracy first, in line with its weaker memory retention in Fig. 2E.

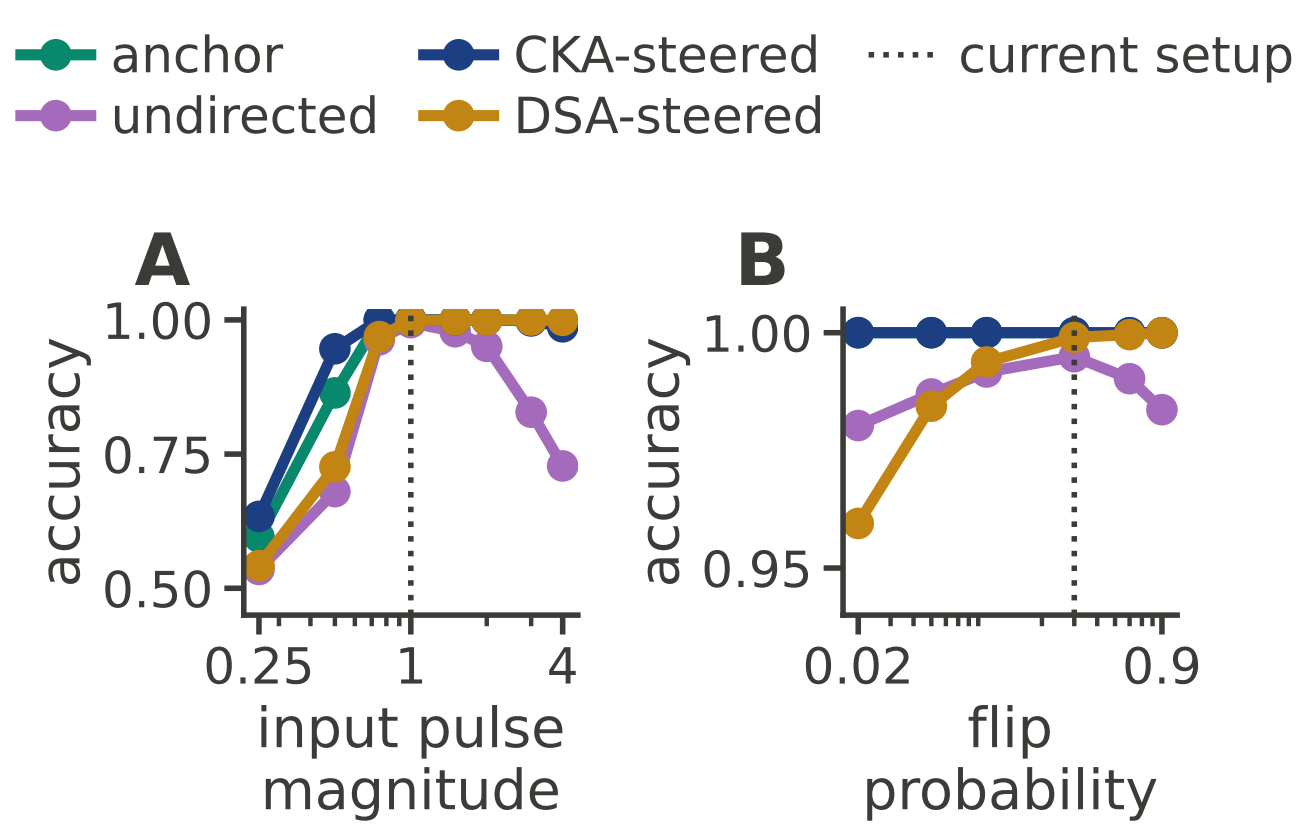

Figure 21: **Out-of-distribution input probes of the walk endpoints.** Accuracy of the anchor and the three walk endpoints when the input statistics differ from training. The dotted line marks the trained value. **(A)** All input pulses scaled by a common magnitude. **(B)** Pulse probability per step. Rare pulses leave long stretches without input within a trial.

G Additional experiments on ViT

G.1 Smooth surrogate for the max-over-layers CKA

Section 4 steers the walk to lower the highest CKA between any layer of the anchor and any layer of the current network. Let $c_{ij}(\boldsymbol{\theta})$ be the linear CKA between the CLS-token features of layer i of the anchor and layer j of the current network on the $N = 512$ probe images, for $i, j = 1, \ldots, L$ with $L = 13$ (one per transformer block plus the pre-logits features). The hard maximum $\max_{i,j} c_{ij}$ is not smooth: its gradient comes from a single layer pair and jumps whenever another pair takes over. The walk therefore ascends a smooth version,

$$\varphi_{\text{CKA}}(\boldsymbol{\theta}) \;=\; 1 - \frac{1}{\beta} \log \sum_{i,j=1}^{L} \exp\bigl(\beta\, c_{ij}(\boldsymbol{\theta})\bigr), \qquad \beta = 30, \tag{12}$$

which replaces the maximum by a log-sum-exp. For large β the log-sum-exp stays close to the maximum. Its gradient is a weighted average of the gradients of all c_{ij}, with most of the weight on the most similar layer pairs, so each step pushes down whichever pairs are currently closest to the maximum. The kNN potential of Eq. 13 uses the same construction with the soft mutual-kNN alignment in place of c_{ij}. Throughout, we report the exact hard maximum (Fig. 3).

G.2 Scoring the visual attributes of the most similar image pairs

Figure 3E asks what the anchor and the endpoint have in common when they represent two images similarly. For the anchor and the HNC endpoint network, we rank all possible pairs of probe images by the cosine similarity of their penultimate-layer features and keep the five highest-ranked pairs. Every pair of probe images is then scored on three visual attributes that are independent of the class identity of each image:

- *Semantic content.* Cosine similarity between the class-token features of a DINOv2 ViT-S/14 (Oquab et al., 2024). This model is trained without labels, so it cannot inherit the anchor's supervision.
- *Color.* Cosine similarity between $8 \times 8 \times 8$ HSV color histograms. Each pixel is described by three numbers: its hue, saturation, and value. Each of the three axes is divided into eight bins, giving 512 bins in total, and we count how many of the image's pixels fall into each one and normalize the sum of counts to one. The histogram records only what colors an image contains and in what proportions, and is unchanged if the pixels are rearranged.
- *Spatial layout.* Correlation between the images downsampled to 32×32 grayscale, which keeps the coarse arrangement of light and dark regions and discards color and fine detail.

The three scores are on different scales, so we report each one as a percentile within the distribution of that score over all pairs of probe images. A pair drawn at random sits at the 50th percentile by construction, which sets the dashed reference line, and a high percentile means the network's closest pairs share that attribute more than two arbitrary images do. The plotted value is the mean percentile over the five pairs.

G.3 Steering by mutual k-nearest neighbor

Section 4 steers the linear CKA. Here we repeat the experiment with the other similarity score used by the PRH, the mutual k-nearest-neighbor alignment of Huh et al. (2024) (Figure 22). The anchor is the same ImageNet-trained ViT-S/16, the function is held fixed on 256 Places-365 images, and the score follows the PRH exactly: for each image, the fraction of its $k = 10$ nearest neighbors that the two representations share, averaged over images and taken at its maximum over all layer pairs. Nearest-neighbor sets change discontinuously as the weights move, so they cannot be steered directly. We therefore relax them: image b counts as a neighbor of image a with a weight that decays smoothly with how far b lies beyond the k-th nearest neighbor of a. Writing $d_{ab}^{(l)}$ for the squared distance between the layer-l features of a and b, and $r_a^{(l)}$ for the squared distance from a to its k-th nearest neighbor, the soft neighborhood at layer l is $S_{ab}^{(l)}(\boldsymbol{\theta}) = \sigma\bigl((r_a^{(l)} - d_{ab}^{(l)})/w_l\bigr)$, where σ is the logistic sigmoid, $r_a^{(l)}$ is held constant when differentiating, and the width w_l is set once from

the anchor at 5% of the median $r_a^{(l)}$ over the probe. The steering potential is the soft dissimilarity

$$\varphi_{\mathrm{kNN}}(\boldsymbol{\theta}) \;=\; 1 - \frac{1}{\beta}\log\sum_{i,j=1}^{L}\exp\big(\beta\, m_{ij}(\boldsymbol{\theta})\big), \qquad m_{ij}(\boldsymbol{\theta}) \;=\; \frac{1}{Nk}\sum_{a\neq b}\bar{S}_{ab}^{(i)}\, S_{ab}^{(j)}(\boldsymbol{\theta}), \tag{13}$$

where m_{ij} measures how much of the anchor's neighborhood structure at layer i (the fixed set $\bar{S}^{(i)}$, computed at $\boldsymbol{\theta}_0$) survives in layer j of the current network, $N = 256$ is the probe size, $L = 13$ counts the class-token layers (one per block plus the pre-logits feature), and the log-sum-exp with $\beta = 30$ is a smooth stand-in for the maximum, as in Eq. 12. Raising φ_{kNN} therefore pushes down the alignment at whichever layer pair is currently the most similar. We report the exact hard score throughout.

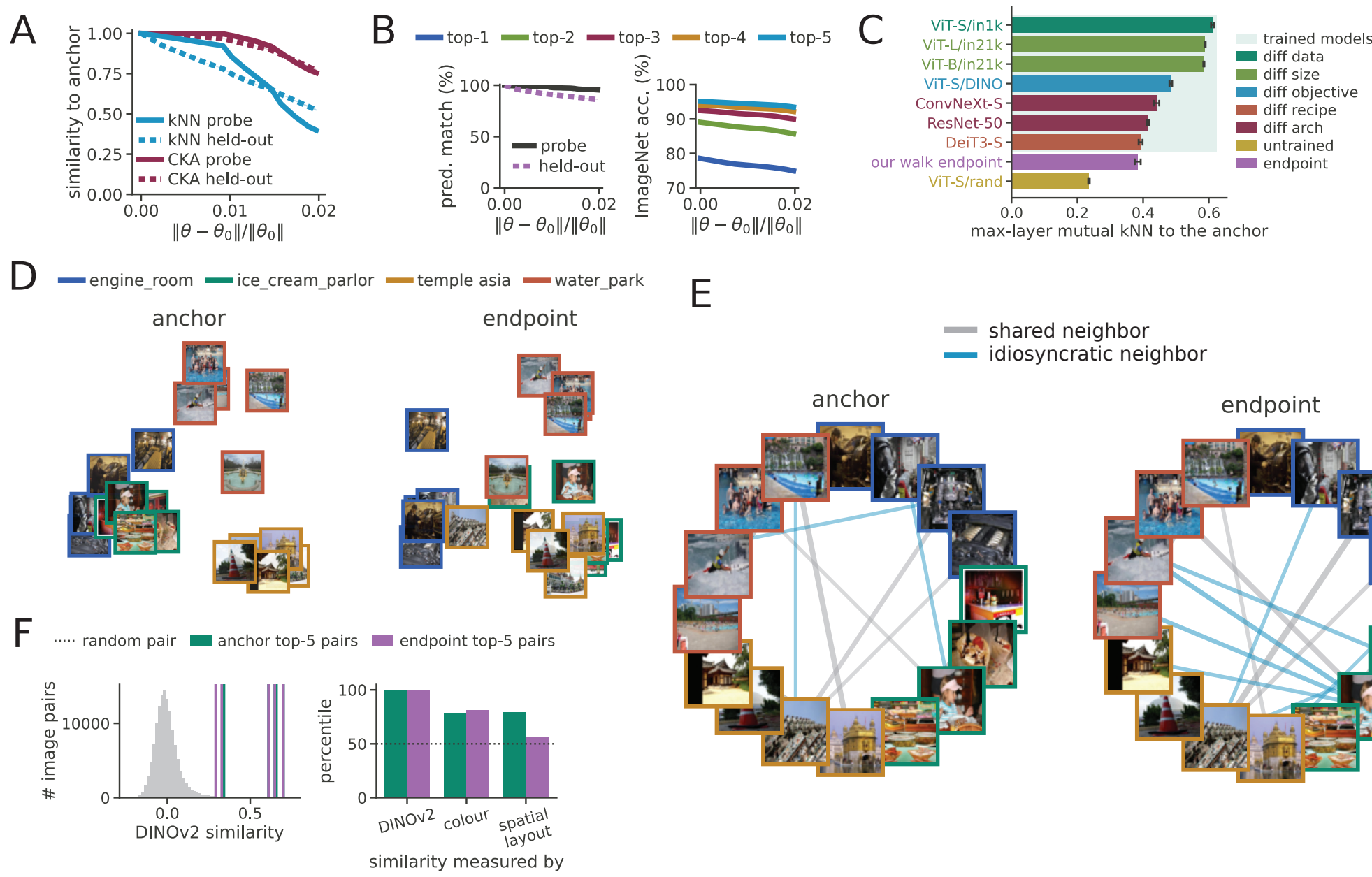

Figure 22: **Steering the mutual kNN score finds alternative representations at preserved input-output mapping.** **(A)** The max-over-layers mutual kNN similarity to the anchor falls along the walk, both on the probe images and on held-out images never seen during HNC. The max-over-layers CKA falls as well, even though the walk does not steer it. **(B)** The top-k predictions on Places-365 images and the top-k accuracy on ImageNet are largely preserved along the walk. **(C)** At the endpoint, the max-layer mutual kNN to the anchor sits below every independently trained model and above only an untrained initialization. **(D)** MDS embedding of 20 images from four classes, for the anchor and the endpoint. **(E)** Nearest-neighbor graph of the same images. Grey edges mark neighbors shared by the anchor and the endpoint; blue edges mark neighbors found in only one of the two networks. **(F)** The most similar image pairs of the endpoint match those of the anchor in semantic content (DINOv2) and color, but their spatial-layout similarity falls toward chance.

G.4 Hyperparameters of the ViT walks

Table 7: Settings of the CKA-steered ViT-S walk in Fig. 3.

Hyperparameter	Value
Anchor	ImageNet-trained ViT-S/16, 22M parameters
Probe set	512 Places-365 images
Flat directions	soft projection with damping $\mu = 10^{-7}\lambda_1$
Solver	preconditioned conjugate gradient, 40 iterations
Steering objective	φ_{CKA} of Eq. 12, $\beta = 30$
First step	random direction, soft-projected
Restore steps	6 per null-space step
Null-space step size η	starts at 0.05; halved on rejection down to 10^{-3}
Loss ceiling	10^{-2}
Relinearization	at every step
Walk length	up to 336 steps

Table 8: Settings of the mutual-kNN-steered ViT-S walk in Figure 22.

Hyperparameter	Value
Anchor	ImageNet-trained ViT-S/16, 22M parameters
Probe set	512 Places-365 images
Flat directions	soft projection with damping $\mu = 10^{-7}\lambda_1$
Solver	preconditioned conjugate gradient, 40 iterations
Steering objective	φ_{kNN} of Eq. 13, $\beta = 30$, $k = 10$
First step	random direction, soft-projected
Restore steps	8 per null-space step
Null-space step size η	starts at 0.05; halved on rejection down to 10^{-3}
Loss ceiling	10^{-2}
Relinearization	at every step
Walk length	500 steps

G.5 Functional preservation beyond the probe set for CKA-steered HNC

The CKA-steered walks in Figure 3 constrain the function on only 512 Places-365 images. Although the endpoints match the anchor on these probe images, this constraint does not guarantee that they will preserve its behavior on unseen inputs. We therefore ask how well the endpoints retain the anchor's function beyond the probe set, including under distribution shift.

We evaluate the anchor and the endpoints of the five CKA-steered walks on held-out ImageNet validation images and on three distribution-shifted test sets with the ImageNet label space: ImageNet-V2 (Recht et al., 2019), ImageNet-Rendition (Hendrycks et al., 2021), and ImageNet-Sketch (Wang et al., 2019). For each dataset, we report three complementary measures. Top-k accuracy assesses whether the endpoints retain the anchor's overall task performance. Prediction match measures the fraction of images on which an endpoint makes exactly the same top-1 prediction as the anchor. Error consistency (Geirhos et al., 2020) measures chance-corrected agreement in which images the two networks classify correctly; unlike prediction match, it does not require the networks to select the same class when both are wrong.

We calibrate these measures against two reference networks. ViT-S/in1k has the same architecture as the anchor but is trained only on ImageNet-1k, providing a reference for a separately trained model. The diff-seed networks provide a stricter comparison: they repeat the anchor's final ImageNet-1k fine-tuning stage from the same ImageNet-21k checkpoint, changing only the random seed.

The CKA-steered endpoints closely track the anchor's top-k accuracy on all four datasets, with a small but consistent accuracy deficit across values of k (Figure 23A). Their exact prediction agreement is highest on clean ImageNet and ImageNet-V2 and decreases on Rendition and Sketch (Figure 23B). This decline is expected in part because, when both networks are wrong, prediction match counts them as agreeing only if they select the same incorrect class. Error consistency remains near 0.8 across all four datasets (Figure 23C), indicating that the endpoints continue to succeed and fail on largely the same images as the anchor even as exact prediction agreement decreases. Thus, their similarity to the anchor extends beyond aggregate accuracy to the image-level structure of their errors, although the agreement is not perfect.

The reference networks clarify the strength of this preservation. The endpoints exhibit higher prediction match and error consistency than ViT-S/in1k on every dataset, with the largest difference on Rendition and Sketch. More strikingly, their image-level agreement with the anchor is comparable to, and generally higher than, that of the diff-seed networks, despite their representations having moved much farther from the anchor. The CKA-steered walks therefore produce substantial representational change while retaining the anchor's off-probe behavior at least as well as repeating its final training stage. At the same time, the remaining accuracy loss and prediction disagreement show that this preservation is approximate rather than exact.

A possible alternative explanation is that the low CKA simply reflects damage to the network associated with its modest accuracy loss. We test this by adding Gaussian noise to the anchor's weights at increasing scales and comparing the resulting accuracy loss and CKA with those observed along HNC (Figure 24). Random perturbations matched to the endpoint's accuracy loss leave CKA above 0.98, and even perturbations that reduce accuracy to chance retain substantially higher CKA than the HNC endpoint. By contrast, the CKA-steered walk falls below the untrained-network reference after losing only less than 1% accuracy. The representational displacement produced by HNC therefore cannot be explained as a generic consequence of degraded performance.

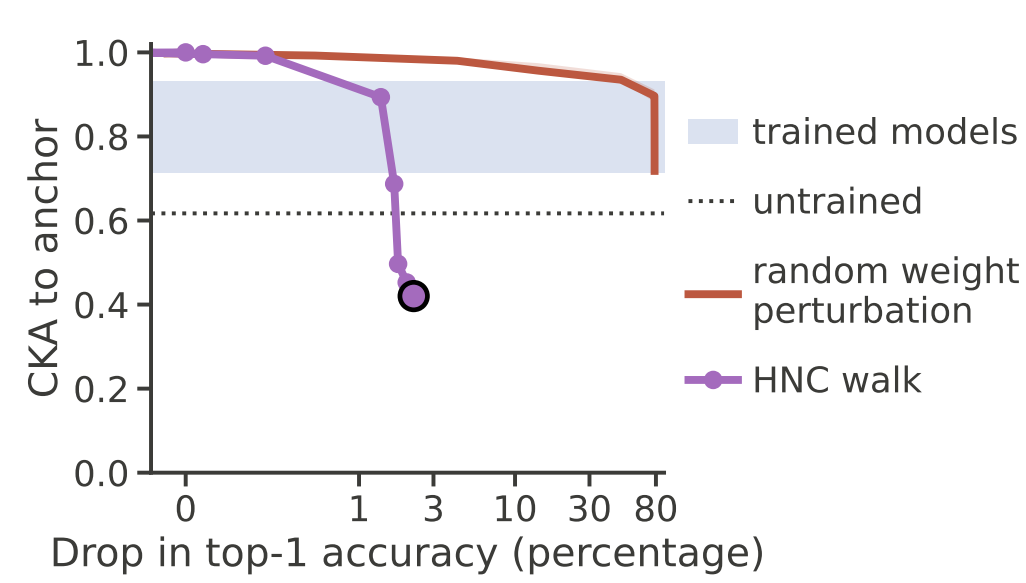

Figure 24: **CKA to the anchor against accuracy lost, for random weight noise and for the HNC walk.** Max-over-layers CKA against the drop in top-1 accuracy on held-out ImageNet validation images, for Gaussian weight noise of increasing scale (three seeds) and for the walk checkpoints up to the reported endpoint (ringed). Band: independently trained models; dotted: untrained.

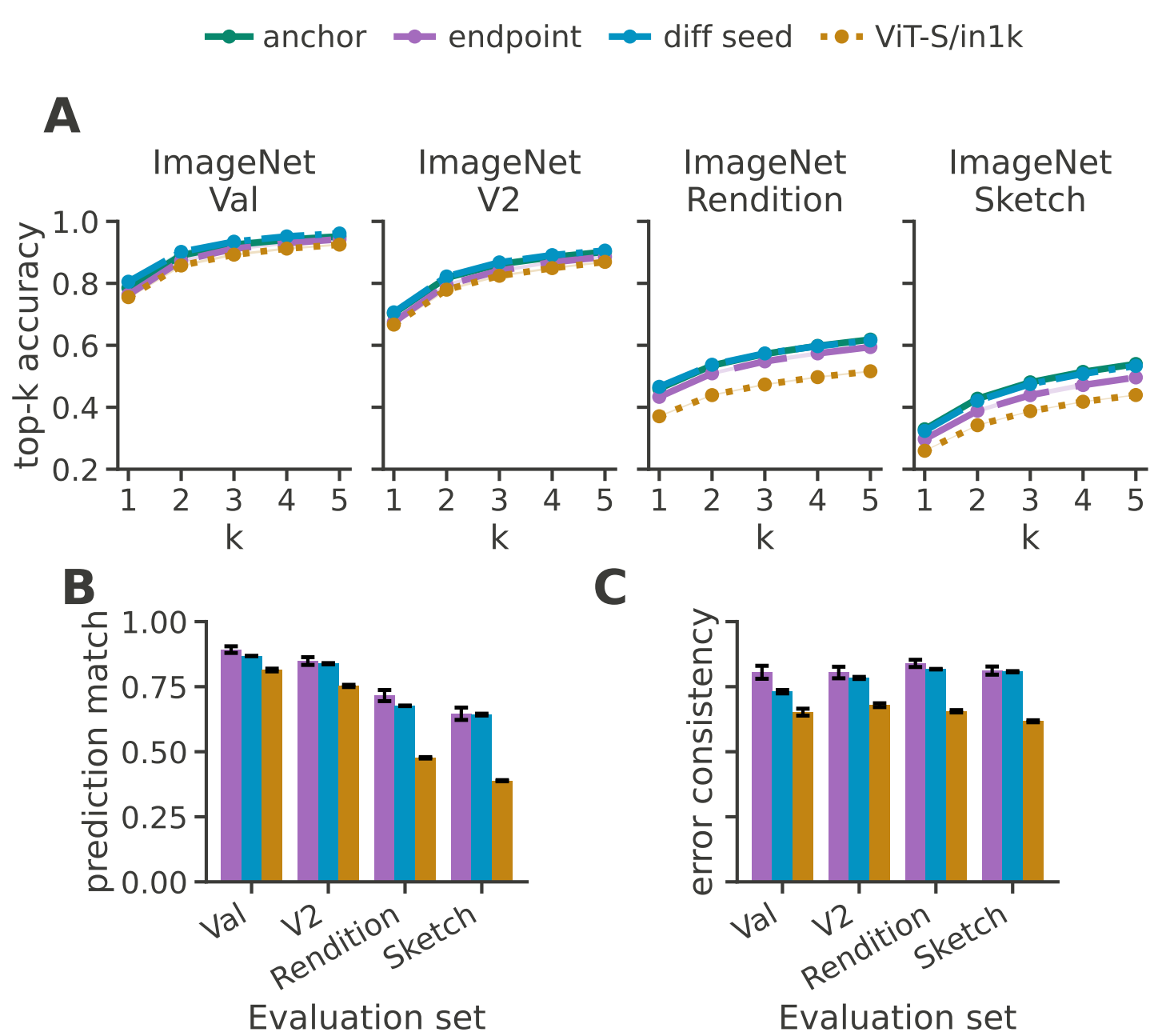

Figure 23: **Accuracy, prediction match and error consistency of the CKA-steered endpoints on four held-out test sets.** **(A)** Top-k accuracy of the anchor, the walk endpoints, a diff-seed network (the anchor's final fine-tuning stage repeated with a different seed) and an independently trained ViT-S/16 (ViT-S/in1k) on clean ImageNet validation images and on ImageNet-V2, ImageNet-Rendition and ImageNet-Sketch. None of these images constrained the walk. **(B)** Fraction of images on which a network makes the same top-1 prediction as the anchor. **(C)** Error consistency with the anchor (chance-corrected agreement on which images are classified correctly). Endpoint curves and bars show the mean and s.d. over the five walks of Figure 3; diff-seed curves and bars the mean and s.d. over two seeds. The anchor and ViT-S/in1k are single networks; their bands and bars show the s.d. over bootstrap resamples of the test images.

G.6 Functional preservation beyond the probe set for mutual-kNN-steered HNC

We next ask whether the representational result persists when HNC is steered with mutual-kNN similarity, which directly measures the preservation of local neighborhoods. Figure 22 shows that mutual-kNN similarity falls on both probe and held-out images and, at the endpoint, lies below that of every independently trained model in the comparison. We evaluate the functional preservation of these endpoints using the same datasets, measures, and reference networks as for the CKA-steered walks.

The mutual-kNN-steered endpoints retain the overall shape of the anchor's top-k accuracy curves, but their accuracy deficit is larger than for the CKA-steered endpoints (Figure 25A). Exact prediction agreement remains substantial, falling from approximately 0.85 on clean ImageNet to approximately 0.6 on ImageNet-Sketch (Figure 25B). Error consistency remains around 0.75 across datasets (Figure 25C), showing that the endpoints still tend to make errors on the same images as the anchor even when they choose different output classes.

The endpoints agree with the anchor more strongly than ViT-S/in1k under both prediction match and error consistency. They nevertheless fall below the diff-seed networks on all four datasets, particularly under distribution shift. The mutual-kNN-steered walks therefore recover the central representational result using a complementary metric, but at this operating point they preserve the anchor's off-probe behavior less faithfully than either the diff-seed networks or the CKA-steered walks. Together, the two experiments show that HNC can move representations beyond the variation observed across trained models while retaining substantial task behavior, but that the degree of functional preservation depends on the walk configuration and steering objective.

This comparison reflects the walk configuration tested here as much as the choice of similarity metric. The probe-size experiment in Section G.9 shows that increasing the probe set improves off-probe preservation. The present results therefore establish a less favorable preservation–displacement tradeoff for the mutual-kNN configuration tested here, rather than an intrinsic limitation of mutual-kNN steering.

To test whether the drop in mutual-kNN alignment simply reflects damage, we add Gaussian noise of increasing scale to the anchor's weights (Figure 26). Noise makes the network as dissimilar to the anchor as an independently trained model only after accuracy has fallen to chance. Noise that lowers accuracy as much as the walk does moves the weights about five times farther than the walk, yet the noisy network stays more similar to the anchor than any independently trained model. The walk, in contrast, becomes less similar to the anchor than every independently trained model after losing only a few points of accuracy.

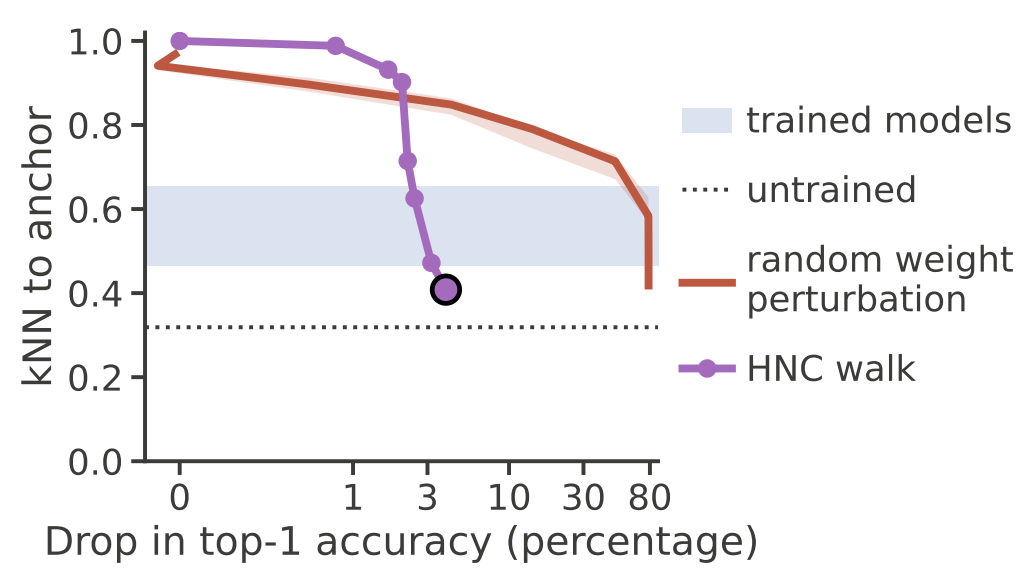

Figure 26: **Alignment to the anchor against accuracy lost, for random weight noise and for the HNC walk.** Max-over-layers mutual-kNN alignment against the drop in top-1 accuracy on held-out ImageNet validation images, for Gaussian weight noise of increasing scale (three seeds) and for the walk checkpoints up to the reported endpoint (ringed). Band: independently trained models; dotted: untrained.

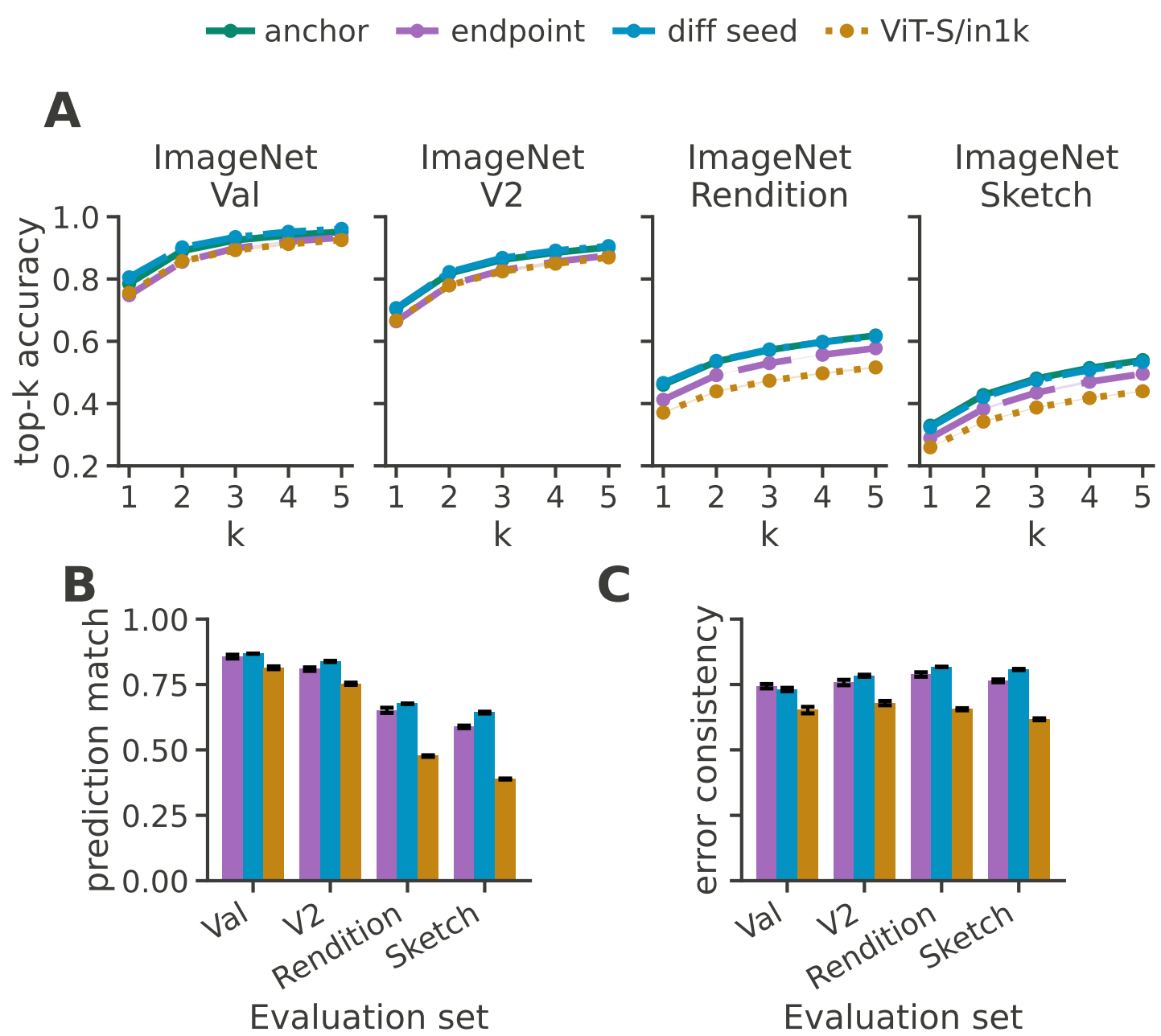

Figure 25: **Accuracy, prediction match and error consistency of the kNN-steered endpoints on four held-out test sets.** Same panels as Figure 23. **(A)** Top-k accuracy of the anchor, the walk endpoints, the diff-seed network and ViT-S/in1k. **(B)** Fraction of images on which a network makes the same top-1 prediction as the anchor. **(C)** Error consistency with the anchor (chance-corrected). Endpoint curves and bars show the mean and s.d. over three kNN-steered walks with different probe draws; diff-seed curves and bars the mean and s.d. over two seeds; anchor and ViT-S/in1k bands and bars the s.d. over bootstrap resamples of the test images.

G.7 Steering to maximize representational divergence at different layers

Section 4 steers the maximum similarity over all layer pairs. Here we aim the walk at a single layer instead, either the pre-logits feature (Figure 27) or the class token after the first block (Figure 28), steering CKA or the mutual kNN score at that layer alone. Both layers can be moved away from the anchor at preserved predictions. Moving the early layer costs more function per unit of similarity lost than moving the pre-logits feature, which is why its figure is shown over a shorter weight distance, but the similarity of early layers across models is not forced by the function either.

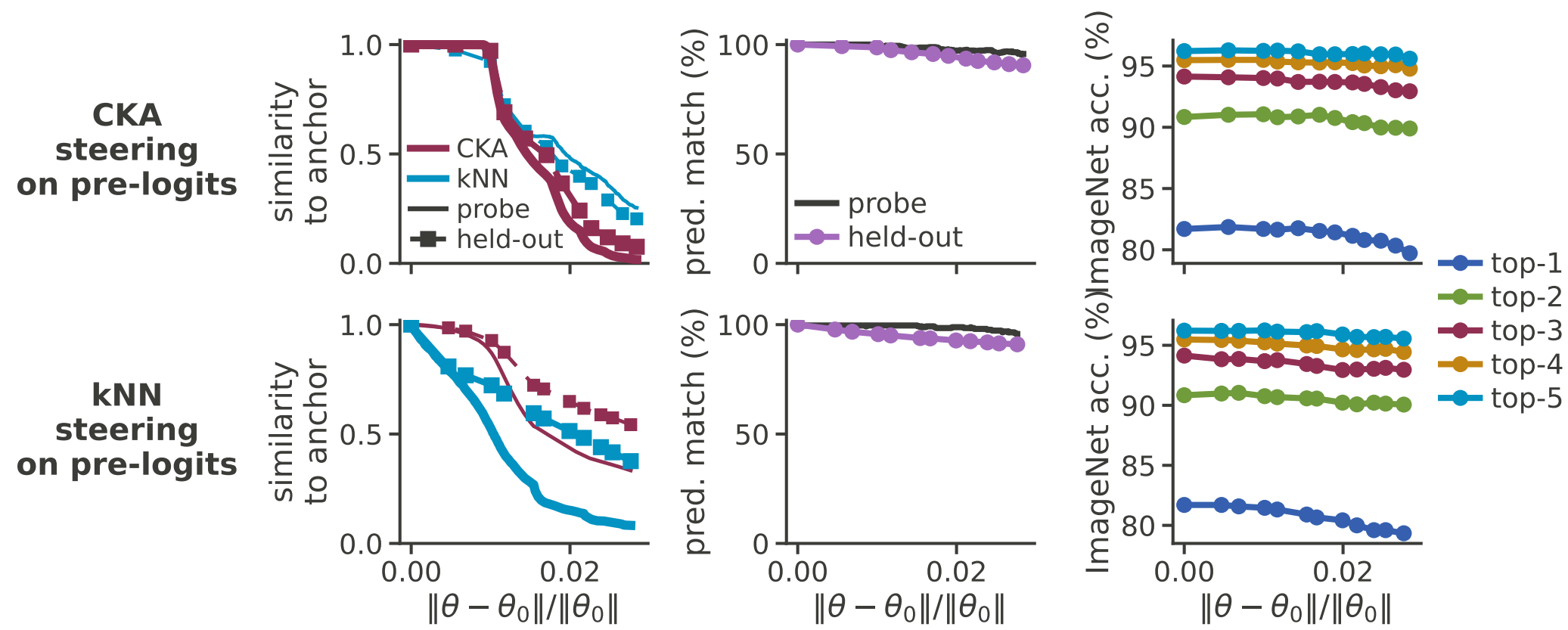

Figure 27: **Steering a single layer: the pre-logits feature.** Top row: CKA-steered walk; bottom row: mutual-kNN-steered walk. Left: similarity of the steered layer to the anchor (thick, steered metric; thin, its unsteered companion) on the probe and held out. Middle and right: function preservation along the same walk.

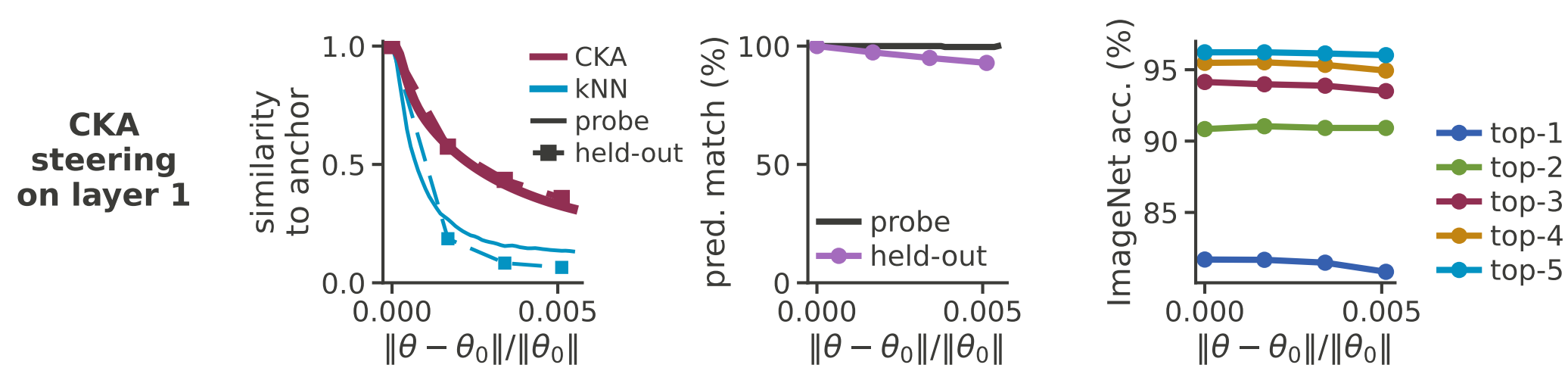

Figure 28: **Steering a single early layer: CKA of the block-1 class token.** Same panels as Figure 27, shown up to a weight distance of 0.005.

G.8 STEERED CONVERGENCE BETWEEN VIT-S AND VIT-B

The experiments so far move a network away from itself. Can a network instead be moved toward a different one? We keep ViT-S as the anchor and take a frozen ViT-B as the target. For each network we take the class token after every block plus the pre-logits feature, thirteen depths in all, and compare the two depth for depth with linear CKA on the same 256 Places-365 images that constrain the function. The walk holds the predictions of ViT-S on those images fixed and steers toward a higher mean similarity to ViT-B over depths.

Averaged over depths, the similarity of ViT-S to ViT-B rises from 0.78 to 0.96 (Figure 29A), while ViT-S keeps 96% of its own predictions on the constrained images (Figure 29B). The gain lands where the two models disagreed most: the anchor already resembles ViT-B in the early blocks and much less in the middle and late ones, and it is those depths that rise, leaving the endpoint uniformly aligned across the network. So the similarity that two independently trained models happen to share is not a ceiling set by the task. A small, function-preserving change reaches well past it, which means that the degree of cross-model convergence, and not only its existence, needs an explanation beyond the function requiring it.

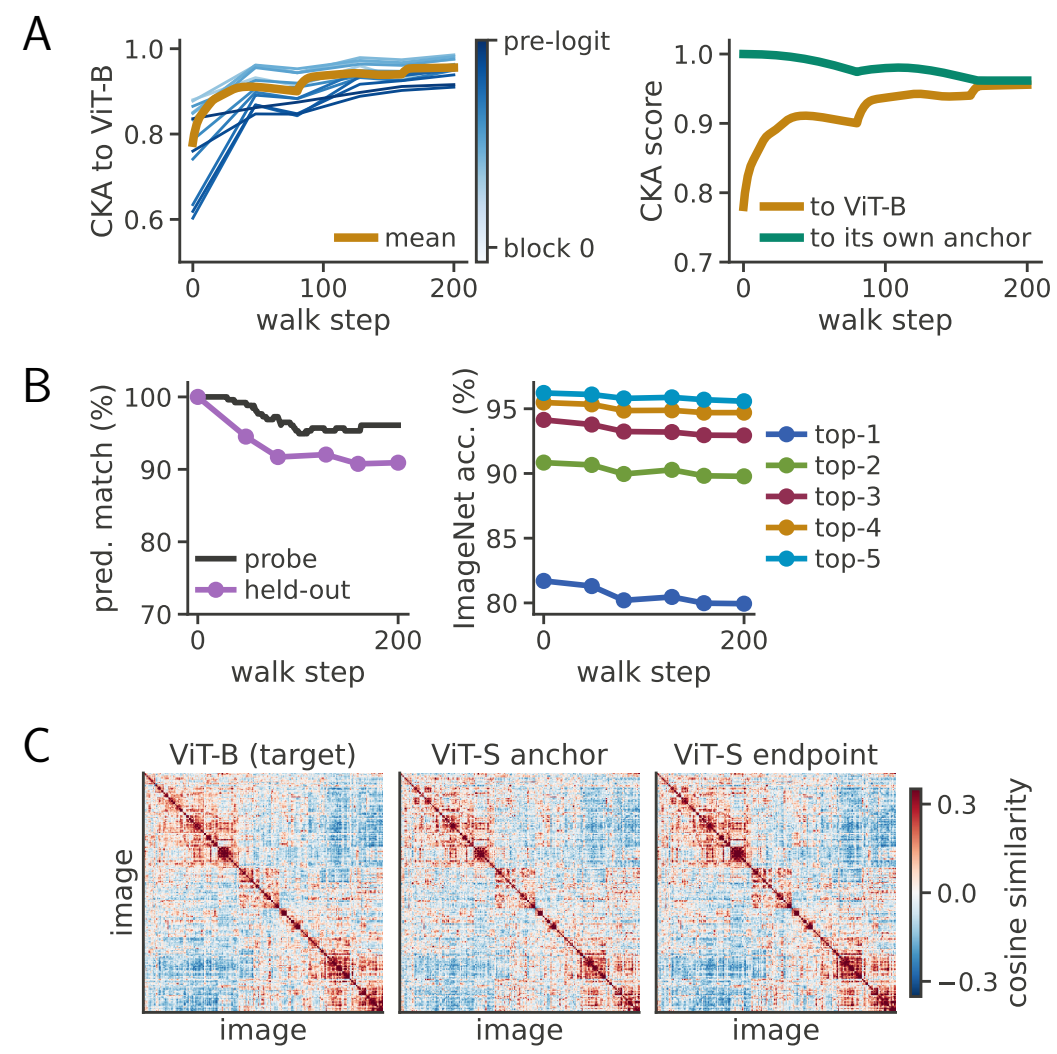

Figure 29: **ViT-S can be steered most of the way to ViT-B without changing what it computes.** **(A)** CKA to the frozen ViT-B over the walk, one thin curve per depth (shaded early to late) under the steered mean, and the same mean against the similarity ViT-S keeps to its own starting representation. **(B)** Function: prediction match to the anchor on the constrained probe and on held-out ImageNet images, and ImageNet top-1 to top-5 accuracy. **(C)** Probe image kernels, in one ordering taken from ViT-B, for ViT-B, the ViT-S anchor, and the ViT-S endpoint.

On 5,000 held-out ImageNet validation images, from a different dataset than the probe, the endpoint keeps nine in ten of the anchor's predictions and loses a little over one point of top-1 accuracy. The kernels tell the same story without a scalar: ordered by the block structure of ViT-B, that structure is faint in the anchor kernel and sharp in the endpoint kernel (Figure 29C). The complementary walk, steering ViT-S away from ViT-B, would bracket the similarity of two trained models from below as well as from above, and we leave it to future work.

G.9 Scaling with the size of the probe set

The walks of Section 4 hold the function fixed on 512 Places-365 images. Here we ask how the size of that probe set shapes the outcome: how far the representation can be steered, how well the function is preserved off the probe, and what the walk costs. We rerun the max-over-layers mutual-kNN walk on ViT-S with everything else unchanged and vary only the probe size. A larger probe set allows more metric displacement and preserves the function better, at a compute cost that grows in proportion (Figure 30). It may seem odd that a tighter constraint lets the representation move farther. The reason is that a larger probe pins the function more firmly, so the walk stays under its loss ceiling for more steps and can spend more of them steering the similarity score.

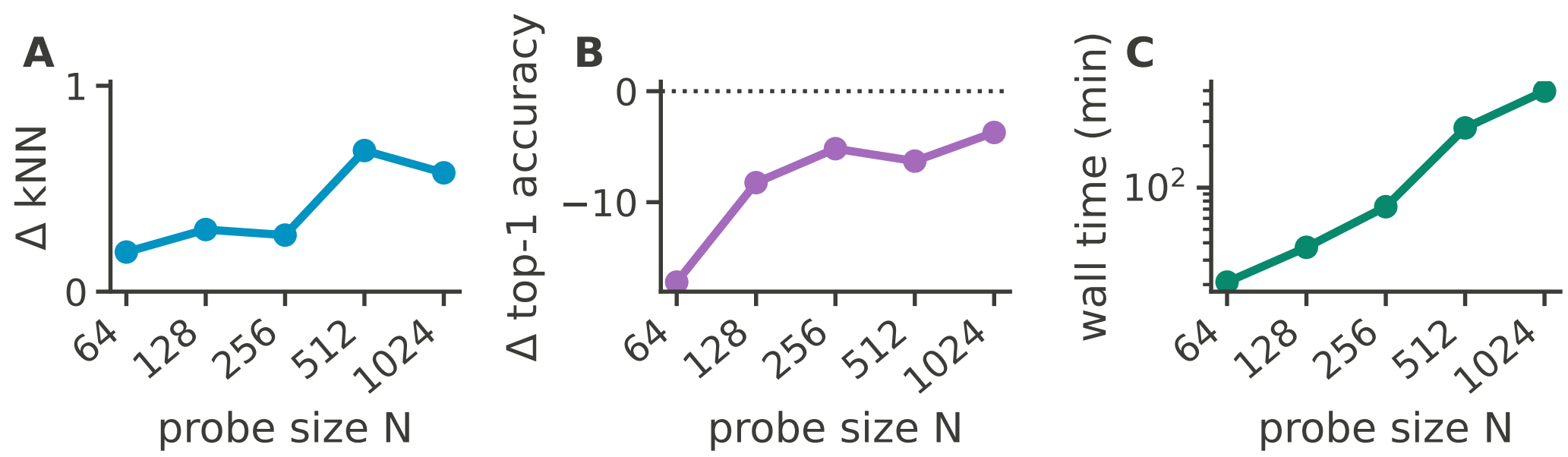

Figure 30: **A larger probe set allows more metric displacement and a better-preserved function, at proportionally more compute.** Each point is one function-preserving max-over-layers mutual-kNN walk on ViT-S. **(A)** Metric displacement at the null-space walk's endpoint **(B)** Function drift measured as the change in top-1 accuracy on 5,000 held-out ImageNet validation images that the walk never constrained on. **(C)** Total wall clock runtime. The per-step cost grows about linearly in N.

G.10 MDS EMBEDDINGS OF THE VIT SOLUTIONS REACHED BY HNC

Section 4 steers the ViT against CKA and Appendix G.3 against mutual-kNN alignment. Here we embed both sets of walks together. Starting from the same ImageNet-pretrained ViT-S/16, we ran CKA-steered and kNN-steered walks on five probe sets of 512 Places-365 images each, keeping ten checkpoints per walk. For each of these ten walks we also ran a walk steered away from its endpoint instead of from the anchor, on the same probe set, as in Appendix E. All checkpoints were evaluated on a shared set of 512 held-out Places-365 images that no walk was steered on. We computed three pairwise distances, the CKA distance and the mutual-kNN distance between penultimate-layer representations and the Euclidean weight distance relative to the anchor norm, and embedded each with metric MDS (Figure 31).

Three patterns hold across the embeddings. First, CKA-steered and kNN-steered walks leave the anchor in different directions under every distance, so the two similarity scores lead to different alternative representations. Second, walks on different probe sets fan out as separate rays rather than converging on one destination. A CKA-steered endpoint and the endpoint repelled from it are further from each other than either is from the anchor. The pretrained network is surrounded by many mutually distinct representations rather than by a single alternative. Third, how far a network appears to have moved depends on the distance used. The kNN-steered walks form a tight cluster near the anchor under CKA distance but spread widely under kNN distance and in weights, whereas CKA steering moves both scores. Every walk moves the weights by a small fraction of the anchor norm while moving the representation far, as in the RNN experiments.

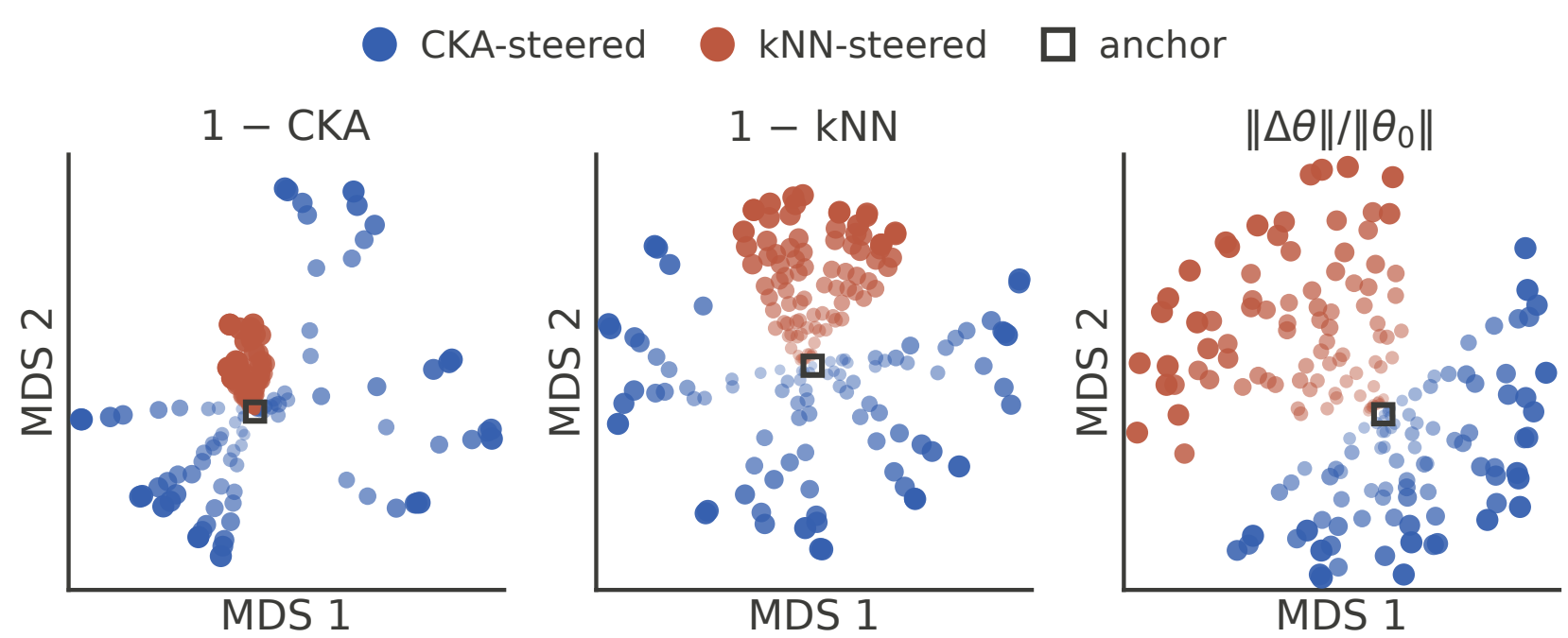

Figure 31: **Solutions reached by CKA- and kNN-steered HNC from a pretrained ViT-S/16, under three distances.** Metric MDS of every checkpoint of CKA-steered (blue) and mutual-kNN-steered (red) walks on five probe sets each, together with walks steered away from those endpoints rather than from the anchor, drawn in the colour of the metric they steer. Marker size and opacity grow along each walk. Distances are computed on 512 held-out Places-365 images that no walk was steered on: representational distance by CKA (left) and by mutual-kNN alignment (middle) of the penultimate layer, and relative weight distance (right). The two steering metrics leave the anchor in separate directions under every distance. kNN-steered walks look compact under CKA distance but spread widely under kNN distance, so how far a representation has moved depends on what is measured.

H STEERING BRAIN-SCORE AT FIXED FUNCTION

A central goal of computational neuroscience is to build predictive models of the brain. To evaluate the neural predictivity of candidate models, Brain-Score (Schrimpf et al., 2018) scores how well a model's internal representations predict recorded neural responses, with benchmark suites for both vision and language models. It is widely used to rank models and to ask which architectures and training objectives yield the most brain-like representations. A model's Brain-Score is usually treated as a fixed property of its architecture, training objective, and training data. Here we ask how much of that score is instead a property of which function-equivalent solution training happened to reach. We take ImageNet-pretrained vision models, hold their logits fixed, and steer HNC to raise or lower how well their features predict recorded neural activity.

The reported metric We report the official neural-predictivity metric of Brain-Score (Schrimpf et al., 2018) on the MajajHong2015 IT benchmark (Majaj et al., 2015): responses of $s = 168$ recording sites in macaque inferior temporal cortex to $n = 3{,}200$ object images. Let $\boldsymbol{F} \in \mathbb{R}^{n \times d}$ be the model's features at a committed layer on those images and $\boldsymbol{Y} \in \mathbb{R}^{n \times s}$ the recorded responses. The metric is a cross-validated linear regression. The images are split into ten folds. On the training images of fold f, a partial-least-squares regression with 25 components fits a linear map $\boldsymbol{W}_f = \mathrm{PLS}_{25}(\boldsymbol{F}_{\mathrm{train}}, \boldsymbol{Y}_{\mathrm{train}})$ from features to responses. On the held-out images of that fold, the predictions $\hat{\boldsymbol{Y}} = \boldsymbol{F}_{\mathrm{test}} \boldsymbol{W}_f$ are compared with the recorded responses one site at a time by Pearson correlation, $r_{j,f} = \mathrm{corr}(\hat{\boldsymbol{Y}}_{:,j}, \boldsymbol{Y}_{:,j})$. The score is the median over sites, averaged over folds, and divided by the noise ceiling of the data (the same correlation computed between two halves of the recorded trials, 0.82 for this benchmark):

$$\mathrm{BrainScore}(\boldsymbol{F}) \;=\; \frac{1}{\mathrm{ceiling}} \cdot \frac{1}{10} \sum_{f=1}^{10} \operatorname*{median}_{j} \; r_{j,f}. \tag{14}$$

Partial least squares is fit by an iterative procedure, so we do not differentiate through it.

The steered surrogate Steering instead uses a simpler quantity that measures the same thing, how well a linear readout of the features generalizes to held-out images. The features are projected onto a 64-dimensional PCA basis fitted once to the anchor and then frozen, $\boldsymbol{Z}(\boldsymbol{\theta}) = (\boldsymbol{F}(\boldsymbol{\theta}) - \boldsymbol{m})\boldsymbol{V}$, which keeps the regression well posed with fewer images than feature dimensions. The steering images are split in half. On the first half a ridge regression is fit, $\boldsymbol{W}(\boldsymbol{\theta}) = (\boldsymbol{Z}_{\mathrm{tr}}^{\top} \boldsymbol{Z}_{\mathrm{tr}} + \lambda \boldsymbol{I})^{-1} \boldsymbol{Z}_{\mathrm{tr}}^{\top} \boldsymbol{Y}_{\mathrm{tr}}$, and on the second half its predictions are correlated with the recorded responses site by site,

$$\varphi_{\mathrm{pred}}(\boldsymbol{\theta}) \;=\; \frac{1}{s} \sum_{j=1}^{s} \mathrm{corr}\big(\boldsymbol{Z}_{\mathrm{ev}}(\boldsymbol{\theta})\, \boldsymbol{W}(\boldsymbol{\theta})_{:,j},\; \boldsymbol{Y}_{\mathrm{ev},j}\big). \tag{15}$$

Every step of this computation is differentiable in $\boldsymbol{\theta}$, so the walk can ascend φ_{pred} to raise predictivity or descend it to lower predictivity. The surrogate and the official metric are different quantities, computed with different regressions and, in our held-out evaluations, on different images and sites. A change in the official score is therefore not the target of the optimization.

Results On two models the official score moves substantially while the function barely changes (top-1 decisions preserved on at least 94% of images, logit KL between 10^{-5} and 10^{-3}). The first is the top model on the Brain-Score neural leaderboard, an 86M-parameter ViT with relative-position embeddings. Its IT predictivity moves from 0.618 to 0.719 between the down and up endpoints, a range of about 0.10 on the images and sites used for steering, and still 0.039 on sites and images that neither the walk nor the surrogate ever saw. The second is a plain ViT-S/16 (22M parameters), the lowest scorer among the models we compare. It moves almost as far, from 0.546 to 0.627 on held-out images, so steerability does not depend on starting from a high-scoring model. For comparison, the spread across seven architectures run through the same pipeline is 0.074. The range opened within a single network at fixed function therefore matches or exceeds the entire gap across architectures (Figure 32). The change is also specific to the steered region: walks that move IT predictivity leave the predictivity of V1, V2, and V4 essentially unchanged.

Therefore, a model's neural predictivity is not fully determined by its architecture, objective, and data alone. It also depends on which of the many function-equivalent solutions the optimizer happened to land on.

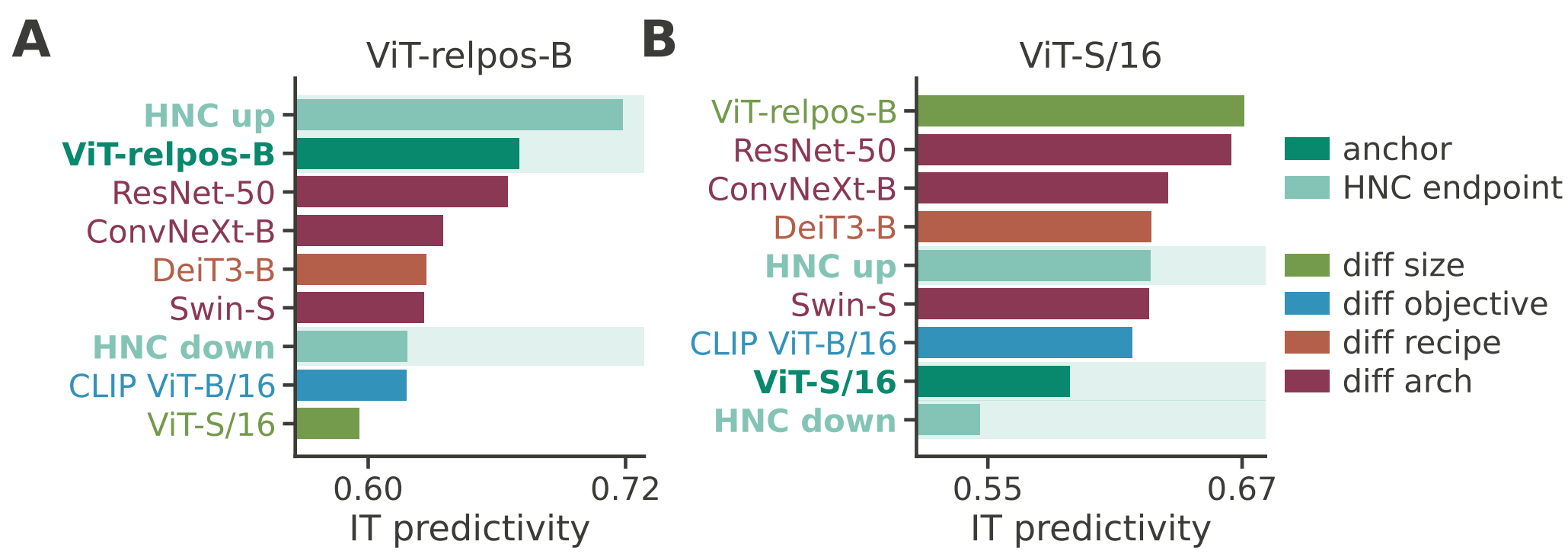

Figure 32: **Steering one network at fixed function moves its Brain-Score as much as switching architectures.** IT predictivity on MajajHong2015 (PLS-25, ceiling-normalized, the same pooled pipeline for every bar) for two anchors and their up and down HNC endpoints, alongside the other leaderboard models. **(A)** The top model of the neural leaderboard, a ViT-B with relative-position embeddings. Its up endpoint scores above every trained model, and the range between its two endpoints (0.618 to 0.719) is wider than the whole spread across models (0.075). **(B)** A plain ViT-S/16, the lowest scorer of the seven. Its range (0.546 to 0.627) still matches the spread across models, and its down endpoint falls below every trained model. Colors mark what mainly distinguishes each comparison model from the panel's anchor; shaded rows mark the anchor and its endpoints.

I EXTENDING HNC TO REINFORCEMENT LEARNING

I.1 ADVANTAGE ESTIMATES

For plume tracking and Ant, the advantages come from generalized advantage estimation (Schulman et al., 2016). Let r_t be the reward at step t of an episode and $V(s_t)$ the critic's value estimate. The advantage is a discounted sum of one-step errors,

$$\delta_t \;=\; r_t + \gamma\, V(s_{t+1}) - V(s_t), \qquad A_t \;=\; \sum_{l\geq 0} (\gamma\lambda)^l\, \delta_{t+l}, \tag{16}$$

with $\gamma = 0.99$ and $\lambda = 0.95$. The sum stops at the end of the episode, where $V(s_{t+1})$ is set to zero. The value estimates come from the network's own critic at the time of collection. The walk never moves the value head. The boat race walks use no critic. There, the advantage of a step is the discounted proxy return from that step to the end of the episode T,

$$A_t \;=\; \sum_{k=t}^{T} \gamma^{\,k-t}\, r_k, \tag{17}$$

with $\gamma = 0.9$ as in training. In all three tasks, we standardize the advantages over the buffer, as in PPO,

$$A_i^0 \;=\; \frac{A_i - \bar{A}}{\sigma_A}, \tag{18}$$

where $\bar{A}$ and σ_A are the mean and standard deviation of the advantages in the buffer. At the reference every importance ratio equals one, so ϕ_R reduces to the mean of the A_i^0 and is zero.

I.2 BEHAVIORAL DIVERGENCE FOR CONTINUOUS AND DISCRETE ACTION SPACES

The steering potential of the reinforcement-learning walks is the KL divergence between the anchor's and the alternative's action distributions, averaged over the buffer states $\{s_i\}_{i=1}^N$,

$$\phi_B(\theta) \;=\; \frac{1}{N}\sum_{i=1}^{N} D_{\mathrm{KL}}\big(\pi_{\theta_0}(\cdot \mid s_i)\,\big\|\,\pi_\theta(\cdot \mid s_i)\big). \tag{19}$$

This section gives its exact form in each action space and shows why it reduces to a mean-squared difference of mean actions in the continuous case.

Discrete action spaces The policy network outputs one logit per action and the policy is their softmax, so the KL divergence is computed directly from the two networks' action probabilities:

$$\phi_B(\theta) \;=\; \frac{1}{N}\sum_{i=1}^{N}\sum_{a\in\mathcal{A}} \pi_{\theta_0}(a \mid s_i)\, \log\frac{\pi_{\theta_0}(a \mid s_i)}{\pi_\theta(a \mid s_i)}, \tag{20}$$

where $\mathcal{A}$ is the discrete action set.

Continuous action spaces The policies are Gaussian, $\pi_\theta(\cdot \mid s) = \mathcal{N}\big(\mu_\theta(s), \Sigma\big)$, where the network outputs the state-dependent mean action $\mu_\theta(s) \in \mathbb{R}^d$ and the covariance $\Sigma = \mathrm{diag}(\sigma_1^2, \ldots, \sigma_d^2)$ is state-independent and held frozen at the anchor's value throughout the walk. For two d-dimensional Gaussians,

$$D_{\mathrm{KL}}\big(\mathcal{N}(\mu_0,\Sigma_0)\,\big\|\,\mathcal{N}(\mu_1,\Sigma_1)\big) \;=\; \tfrac{1}{2}\Big[\mathrm{tr}\big(\Sigma_1^{-1}\Sigma_0\big) - d + (\mu_1-\mu_0)^\top\Sigma_1^{-1}(\mu_1-\mu_0) + \log\tfrac{\det\Sigma_1}{\det\Sigma_0}\Big]. \tag{21}$$

Because both policies share the same frozen covariance ($\Sigma_0 = \Sigma_1 = \Sigma$), the trace term equals d and the log-determinant term vanishes, so only the quadratic term in the means survives:

$$D_{\mathrm{KL}}\big(\pi_{\theta_0}(\cdot \mid s)\,\big\|\,\pi_\theta(\cdot \mid s)\big) \;=\; \tfrac{1}{2}\,\big(\mu_\theta(s)-\mu_{\theta_0}(s)\big)^\top\Sigma^{-1}\big(\mu_\theta(s)-\mu_{\theta_0}(s)\big) \;=\; \sum_{j=1}^{d}\frac{\big(\mu_{\theta,j}(s)-\mu_{\theta_0,j}(s)\big)^2}{2\sigma_j^2}. \tag{22}$$

Averaged over the buffer, ϕ_B is therefore a per-dimension weighted mean-squared difference between the two policies' mean actions. Because the covariance is frozen, the weights $1/(2\sigma_j^2)$ are constants of the walk, and we maximize the unweighted form

$$\phi_B^{\mathrm{MSE}}(\theta) \;=\; \frac{1}{N}\sum_{i=1}^{N}\big\|\mu_\theta(s_i)-\mu_{\theta_0}(s_i)\big\|^2, \tag{23}$$

which coincides with Eq. 19 up to an overall constant when the σ_j are equal across action dimensions, and otherwise differs from it only by the fixed diagonal reweighting.

I.3 Hyperparameters of the reinforcement-learning walks

Table 9: Settings of the plume-tracking walk in Fig. 4.

Hyperparameter	Value
Anchor	PPO-trained 64-unit recurrent policy
Buffer	8 episodes of the current policy, re-collected every 5 steps
Flat directions	soft projection with damping $\mu = 10^{-5}\lambda_1$
Solver	conjugate gradient, 50 iterations
Steering objective	ϕ_B^{MSE} of Eq. 23
Restore steps	15 per null-space step
Null-space step size η	starts at 0.02; halved on rejection down to 2×10^{-3}
Loss ceiling	4×10^{-4}
Walk length	30 steps
Evaluation	every 5 steps on 60 fixed initial conditions with common random numbers

Table 10: Settings of the boat race walks in Fig. 4.

Hyperparameter	Value
Anchor	ten PPO-trained two-layer policies (Appendix I.6)
Buffer	32 episodes of the current policy with ϵ-greedy exploration, $\epsilon = 0.25$; re-collected every 3 steps
Flat directions	soft projection with damping $\mu = 10^{-5}\lambda_1$
Solver	conjugate gradient, 50 iterations
Steering objective	ϕ_B of Eq. 20
Restore steps	15 per null-space step
Null-space step size η	starts at 0.02; halved on rejection down to 2×10^{-3}
Loss ceiling	10^{-4}
Walk length	120 steps
Evaluation	every 5 steps over 100 episodes

Table 11: Settings of the MuJoCo Ant walk in Appendix I.7.

Hyperparameter	Value
Anchor	PPO-trained feed-forward actor with 256 hidden units
Buffer	4,000 transitions of the current policy, re-collected every 5 steps
Flat directions	soft projection with damping $\mu = 10^{-5}\lambda_1$
Solver	conjugate gradient, 50 iterations
Steering objective	ϕ_B^{MSE} of Eq. 23
Restore steps	15 per null-space step
Null-space step size η	starts at 0.08; halved on rejection down to 2×10^{-3}
Loss ceiling	4×10^{-4}
Walk length	until the action divergence reaches 1.0 (19 steps)
Evaluation	every 5 steps on 30 fixed initial states with common random numbers

I.4 Success rate along the plume-tracking walk

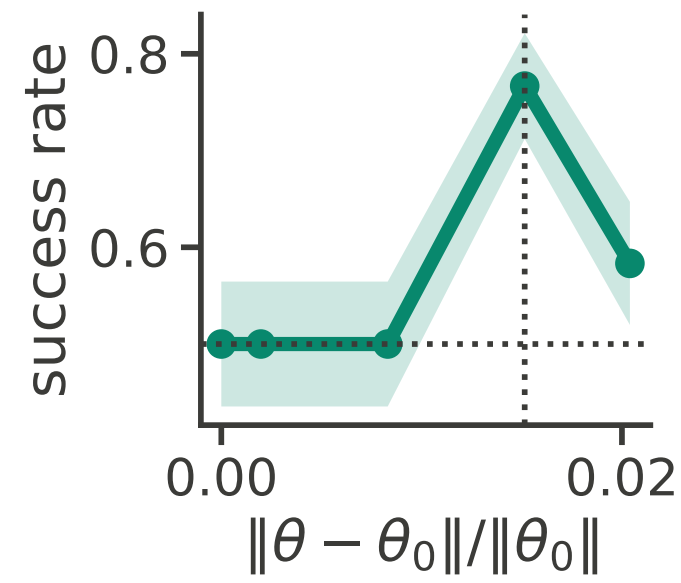

Figure 33: **Success rate along the plume-tracking walk.** The fraction of 60 fixed initial conditions in which the agent reaches the odor source, plotted against the relative weight distance from the anchor. Markers show the evaluated walk steps, and shading shows the standard error over initial conditions. The horizontal dotted line marks the anchor's success rate. The vertical dotted line marks the alternative policy shown in Fig. 4.

I.5 Out-of-distribution evaluation of the plume-tracking HNC walk

The plume-tracking result of Section 5 raises the question of how the alternative policy found on the reward level set behaves on conditions the training distribution never shows. We evaluate snapshots along the walk of Fig. 4 under four conditions: the constant-wind training condition, a mid-episode 45° wind switch, a sparse plume with puff density thinned to $0.1\times$, and noisy wind (Figure 34). Each condition uses 240 paired initial conditions with common random numbers, which we split into eight batches of 30 episodes to estimate a standard error of the homing rate. The constant-wind curve doubles as the level-set check, since the walk is still on the reward level set wherever this curve stays at or above the anchor's rate.

Every shifted condition improves along the walk. The anchor fails on every sparse-plume and noisy-wind episode, whereas the alternative policy analyzed in the main text (dotted line in Figure 34) homes on a fraction of them and more than doubles the anchor's homing rate under the wind switch. The wind-switch rate keeps rising past that snapshot, and at step 20 all curves collapse together, which is where the walk leaves the level set. The alternative policy therefore does not pay for its different search strategy with worse generalization. The reward level set around this anchor contains policies that are more robust than the trained one on every shifted condition we tested, so the training reward underdetermines out-of-distribution behavior, and HNC moves along the level set toward the more robust policies.

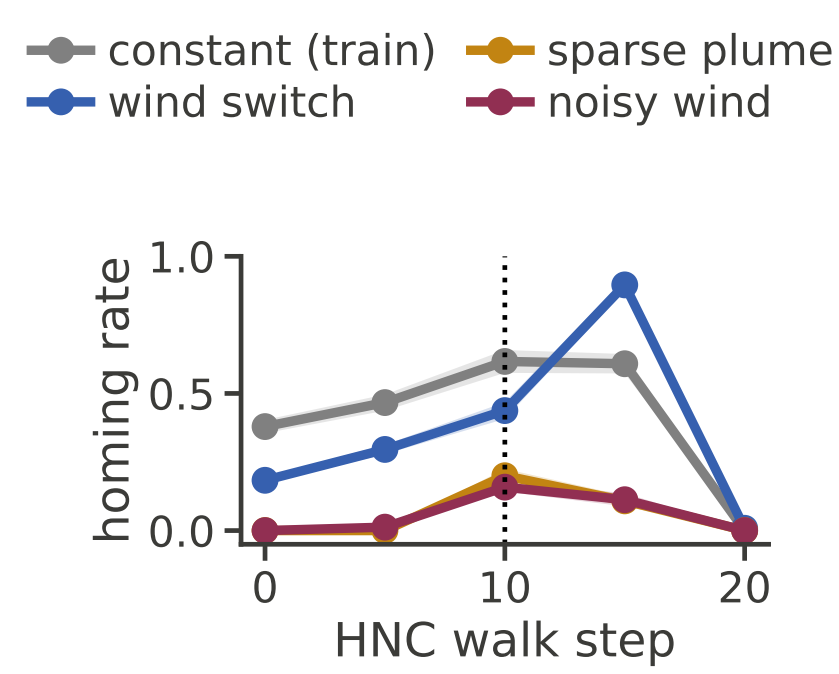

Figure 34: **The alternative plume-tracking policy generalizes better than its anchor on every shifted condition.** Homing rate against the HNC walk step for the walk of Fig. 4, evaluated under the constant-wind training condition and three shifted conditions. Lines and shading show the mean ± s.e.m. over eight batches of 30 paired initial conditions with common random numbers; rates are comparable across steps within a condition but not across conditions, since each condition has its own initial-condition grid. The dotted line marks the alternative policy analyzed in the main text (Fig. 4A,C).

I.6 The boat race policies

Task and rewards The boat race is a 5×5 gridworld from the AI Safety Gridworlds suite (Leike et al., 2017). A square track of four arrow tiles surrounds a wall, and each arrow marks the clockwise direction of travel. The agent has four movement actions and every step costs -1. Entering an arrow tile from the direction the arrow points earns the proxy reward of $+3$, which is the only positive term

the agent ever sees. The environment separately keeps a true return that the agent is not shown. This score rewards each clockwise move around the track and penalizes each counter-clockwise one, so it measures net progress rather than tile entries. The proxy reward admits the exploit described in Section 5: stepping on and off a single arrow tile from the correct side collects $+3$ repeatedly while the true return stays at zero.

Architecture The agent sees the board as a flattened one-hot map, one channel per cell type, giving 175 inputs. A two-layer network with 64 rectified-linear units in each layer feeds a policy head over the four actions and a value head, for 15,749 parameters in total. The walk moves the body and the policy head (15,684 parameters) and leaves the value head frozen, since it plays no role in the surrogate return or the behavior. The board is fully observed, so the policy needs no recurrence.

Training We train the ten anchors with PPO (Schulman et al., 2017) on the true return rather than the proxy reward, which is what makes them well behaved: an anchor trained on the proxy reward alone discovers the exploit by itself, leaving nothing for HNC to find. Each seed runs for 500,000 environment steps with the Adam optimizer at a learning rate of 3×10^{-4}, rollouts of 2,048 steps, four epochs per rollout, minibatches of 256, a clip range of 0.2, an entropy bonus of 0.01, and generalized advantage estimation with $\lambda = 0.95$. Episodes are capped at 200 steps. We set the discount to $\gamma = 0.9$, below the usual 0.99: the track takes several moves to traverse, and at a heavier discount the agent never credits the arrow tile it is heading for and fails to learn the loop at all. All ten anchors converge to the same behavior, circling the track and earning the maximum return under greedy evaluation over 100 episodes.

I.7 MuJoCo Ant locomotion

Besides plume tracking and boat race, we additionally applied HNC to the Ant-v3 task. In Ant-v3, a four-legged robot is rewarded for running forward as fast as possible while staying upright. The anchor is a feed-forward network with two layers of 256 tanh units that outputs the mean torques of the eight joints, trained with PPO (Schulman et al., 2017). The walk runs until the action divergence from the anchor reaches 1.0, which takes 19 steps, with the frozen buffer re-collected every 5 steps.

The walk moves the actions steadily away from the anchor's while the return stays at the anchor's level (Figure 35), and the average forward speed stays in the same range. Random directions in actor space, rescaled to the *same* action-KL, lose more than half of the return. The preserved return therefore comes from the choice of direction within the null space, not from a small step size.

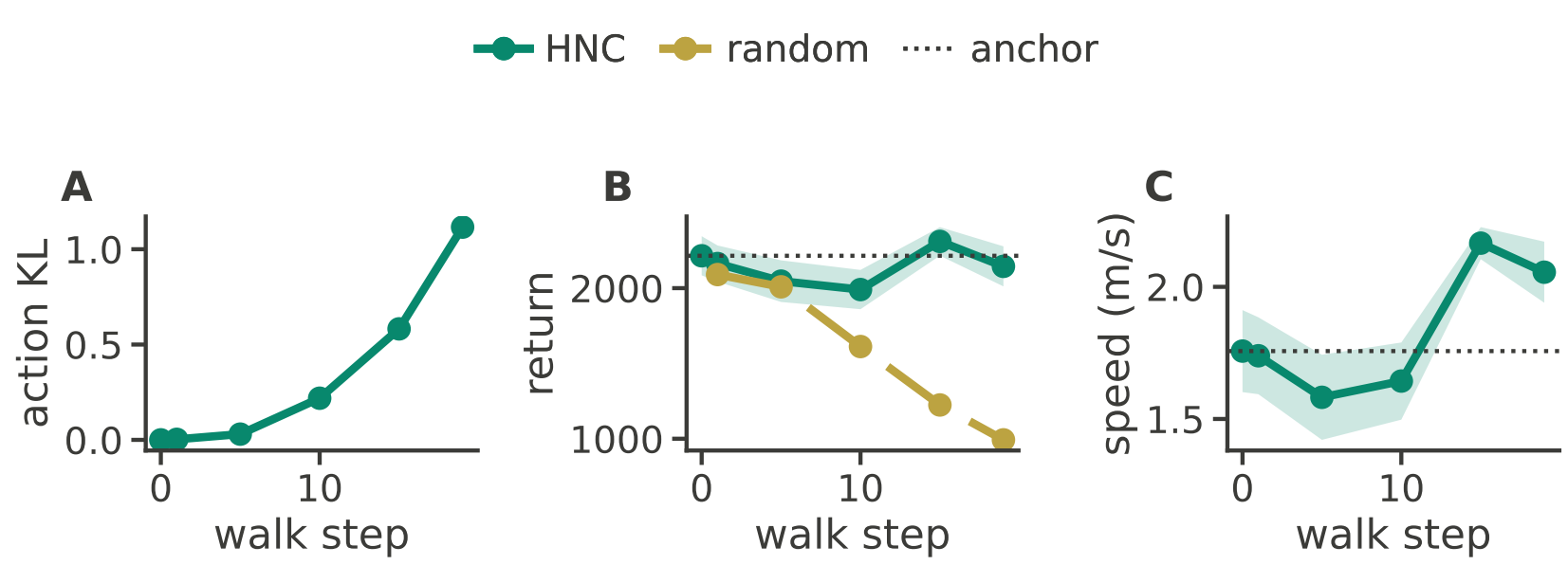

Figure 35: **On Ant-v3, the walk moves the actions while the return is unchanged. (A)** Action-KL from the anchor along the walk. **(B)** Return, mean ± SE over the 30 fixed initial states, against the anchor level (dotted) and against random actor-space directions rescaled to the same action-KL as the snapshot at that step. **(C)** Average forward speed over the same episodes.

What the walk does change is how the ant moves and how it fails (Figure 36). The four legs of Ant form two diagonal pairs. In both policies, the front-right and back-left diagonal swings once per gait cycle, where a cycle is one bounce of the torso. The other diagonal carries an extra beat, and the walk moves this beat from one end of the diagonal to the other. The anchor swings its front-left leg faster than once per cycle, while the alternative slows that leg and speeds up the back-right leg instead. The two policies also fail in different ways. The anchor never ends an episode early, but on a few initial states it stands upright and barely moves for the whole episode. The alternative gets stuck less often, but it sometimes ends an episode early by rearing above the healthy height

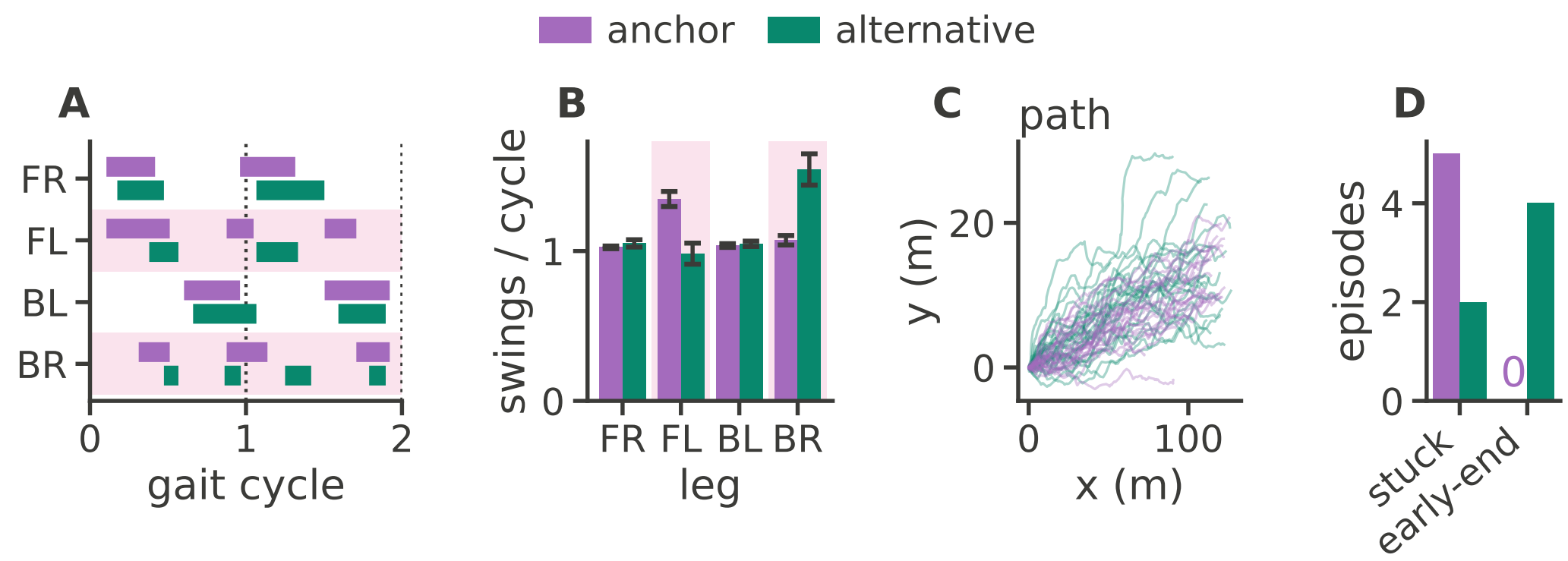

Figure 36: **The alternative policy walks with a different inter-leg pattern and fails in a different way.** Legs are named by position: front-right (FR), front-left (FL), back-left (BL), and back-right (BR). **(A)** Gait diagram from a shared initial state. A bar marks the swing of one leg, with the anchor above and the alternative below within each leg row, and time measured in gait cycles (one bounce of the torso, dotted lines) so that policies running at different tempos remain comparable. **(B)** The tempo of each leg in the same units, mean $\pm$ SE over the episodes in which the agent walks (25 of 30 for the anchor and 27 for the alternative; a stalled episode provides no gait to measure). The pink bands in **(A)** and **(B)** mark the FL-BR diagonal, the pair that is re-timed. **(C)** Torso paths seen from above, one line per episode. **(D)** Episodes that end badly, where *stall* denotes an episode that ran to the 1000-step limit at under 0.5 m/s and *early-end* an episode stopped by the simulator because the torso left the healthy height range.

range. Their per-episode returns are uncorrelated across initial states, so the equal mean return hides two different competence profiles. The reward of Ant sums forward velocity, an upright bonus, and control and contact costs. Leg timing and the set of initial states on which a policy performs well enter none of these terms, and those are precisely what the walk is free to change.

J Probing loss-landscape geometry with HNC

J.1 Architecture and training of the CNN sweep

Architecture Every network in the sweep is a small residual CNN with three stages of width w, $2w$, and $4w$. Each stage is one residual block of two 3×3 convolutions with GroupNorm and ReLU, and the last two stages halve the spatial resolution. Global average pooling gives the feature vector analyzed in subsection J.4, followed by a linear head with one output per class. We use GroupNorm rather than BatchNorm so that each image is processed independently of the rest of the batch. Table 12 lists the parameter counts.

Table 12: Parameter count P of the CNN grid.

Width w	P at $C = 7$	P at $C = 100$
16	75,703	81,748
24	169,039	178,060
32	299,367	311,364
48	670,999	688,948
96	2,673,703	2,709,508

Data A C-way task uses the first C classes of CIFAR-100, with 300 training images per class and no augmentation. The grid crosses widths $w \in \{16, 24, 32, 48, 96\}$ with $C \in \{7, 10, 20, 50, 100\}$ classes, and each cell is trained from three seeds, giving 75 anchors.

Training Networks are trained on the cross-entropy loss with Adam (learning rate 10^{-3}, batch size 256) and a cosine learning-rate schedule. Training stops as soon as the training accuracy reaches

100%, which every anchor does. Stopping at this point makes every anchor an exact zero-loss solution. It also treats the hard cells and the easy cells alike. Test accuracy falls with the number of classes and rises with width, so the grid covers a wide range of generalization while every anchor fits its training data exactly.

Probe set All measurements of Section 6 hold the function fixed on 512 of each anchor's own training images. This keeps the number of output constraints below the number of parameters in every cell.

J.2 Sweeping max loss drift to define the null-space

The null fraction of Section 6 depends on the flatness threshold ϵ. Figure 37 repeats the grid measurement over a wide range of thresholds around the main-text value. At every threshold the null fraction rises with width and falls with the number of classes, so the trends do not depend on the choice of ϵ.

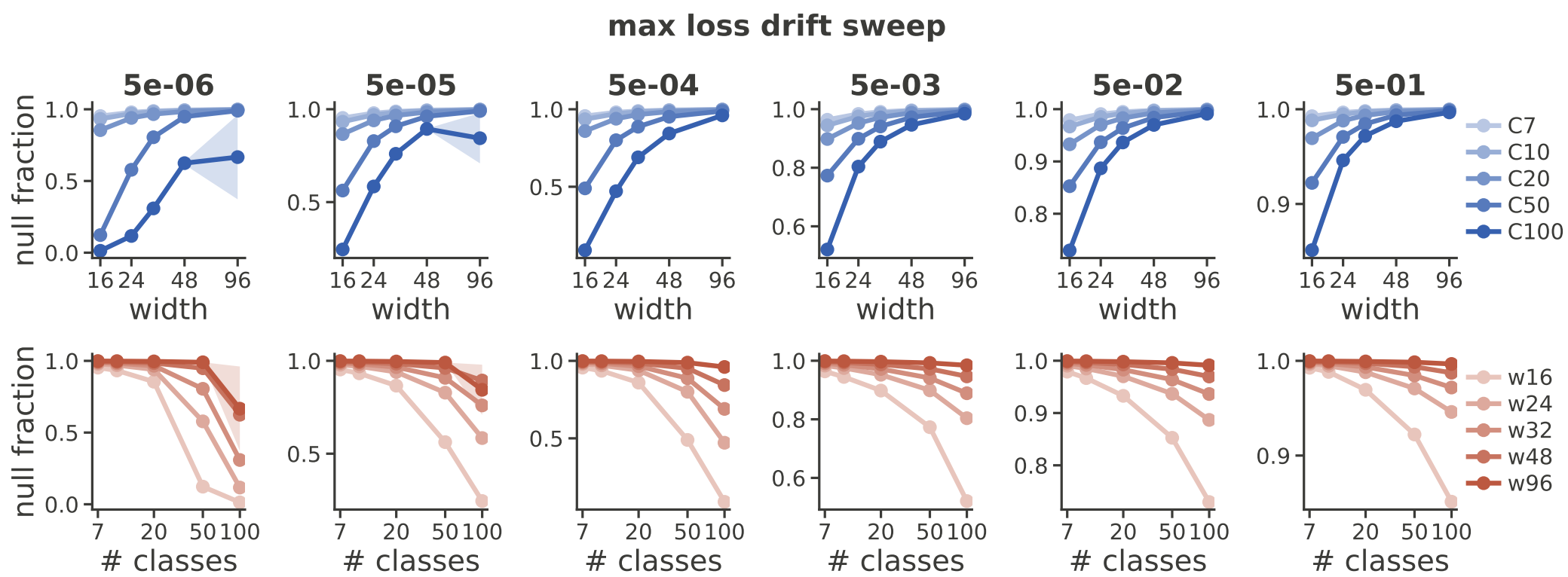

Figure 37: **Null fraction across the grid at every flatness threshold.** Each column is one threshold ϵ. Top: number of classes at each width. Bottom: width at each number of classes. Mean $\pm$ s.e.m. over 3 seeds.

J.3 Per-step curvature at different relative thresholds

The normalized curvature of Section 6 (Fig. 5B) is measured along walks that treat a direction as flat when its Hessian eigenvalue lies below a cutoff μ relative to the largest eigenvalue. Figure 38 repeats the measurement at four cutoffs, with the walks otherwise unchanged (loss ceiling 0.2, 600 steps, three seeds per cell). In every run the endpoint keeps the anchor's predictions on nearly all images. At every cutoff the curvature rises with the number of classes and falls with width. A looser cutoff raises the overall level because it admits stiffer directions, but the ordering of the cells stays the same.

J.4 Representation dimensionality across the sweep

Section 6 uses the number of classes as a proxy for task complexity. Here we check that harder tasks also change the representation itself. On the same grid, we measure the effective dimensionality of the pre-logits features on held-out test images. It is the participation ratio of the feature covariance, a soft count of how many directions carry variance.

Task complexity, not model size, sets the dimensionality (Figure 39A). At a fixed width it grows about fivefold from the easiest to the hardest task, while at a fixed task it barely changes across widths. The cumulative-variance curves show the same pattern (Figure 39B). More classes spread the variance over more directions, while extra width leaves the curves almost unchanged.

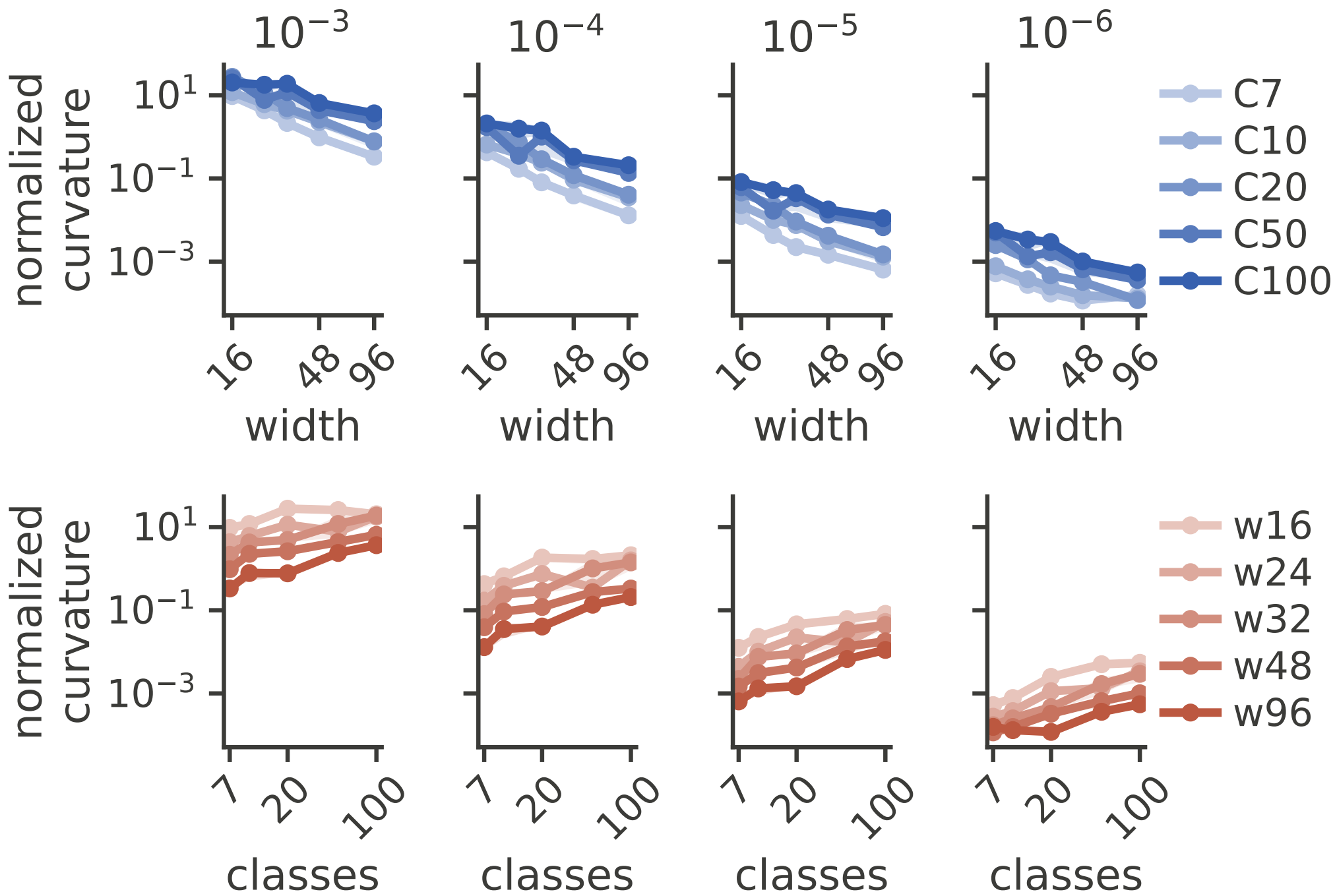

Figure 38: **Normalized curvature across the grid at four relative flatness thresholds.** Each column is one cutoff $\mu/\lambda_{\max}$. Top: number of classes at each width. Bottom: width at each number of classes. Gauss–Newton curvature $d^\top Gd/(nC)$ of each accepted unit step, median over steps, then median ± s.e.m. over 3 seeds.

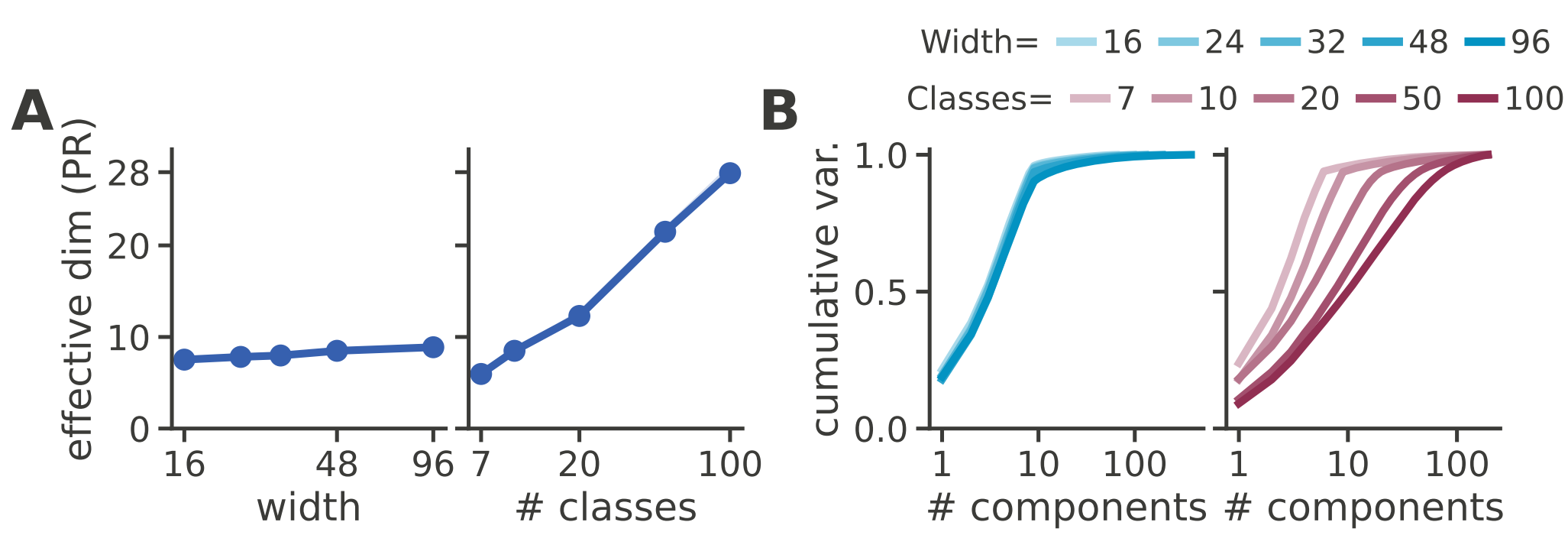

Figure 39: **Harder tasks push the representation into more dimensions; model size barely does.** **(A)** Effective dimensionality (participation ratio) of the held-out representation vs width (at C=10) and vs the number of classes (at w=48); band = min-max over 3 seeds. **(B)** PCA cumulative variance of the representation (mean and min-max band over 3 seeds): width leaves the curves nearly identical (left), task complexity fans them apart (right).
